# Bodipy–C_60_ triple hydrogen bonding assemblies as heavy atom-free triplet photosensitizers: preparation and study of the singlet/triplet energy transfer†
†Electronic supplementary information (ESI) available: Syntheses, structure characterization data, and UV/vis absorption and emission spectra. See DOI: 10.1039/c4sc03865g


**DOI:** 10.1039/c4sc03865g

**Published:** 2015-04-09

**Authors:** Song Guo, Liang Xu, Kejing Xu, Jianzhang Zhao, Betül Küçüköz, Ahmet Karatay, Halime Gul Yaglioglu, Mustafa Hayvali, Ayhan Elmali

**Affiliations:** a State Key Laboratory of Fine Chemicals , Dalian University of Technology , E-208, West Campus , Dalian 116024 , P. R. China . Email: zhaojzh@dlut.edu.cn ; http://finechem2.dlut.edu.cn/photochem ; Fax: +86 411 8498 6236; b School of Chemistry , Dalian University of Technology , Dalian 116024 , P. R. China; c Department of Engineering Physics , Faculty of Engineering , Ankara University , 06100 Beşevler , Ankara , Turkey; d Department of Chemistry , Faculty of Science , Ankara University , 06100 Beşevler , Ankara , Turkey

## Abstract

Supramolecular triplet photosensitizers based on hydrogen bonding-mediated molecular assemblies were prepared. Three thymine-containing visible light-harvesting Bodipy derivatives (**B-1**, **B-2** and **B-3**, which show absorption at 505 nm, 630 nm and 593 nm, respectively) were used as H-bonding modules, and 1,6-diaminopyridine-appended C_60_ was used as the complementary hydrogen bonding module (**C-1**), in which the C_60_ part acts as a spin converter for triplet formation. Visible light-harvesting antennae with *methylated* thymine were prepared as references (**B-1-Me**, **B-2-Me** and **B-3-Me**), which are unable to form strong H-bonds with **C-1**. Triple H-bonds are formed between each Bodipy antenna (**B-1**, **B-2** and **B-3**) and the C_60_ module (**C-1**). The photophysical properties of the H-bonding assemblies and the reference non-hydrogen bond-forming mixtures were studied using steady state UV/vis absorption spectroscopy, fluorescence emission spectroscopy, electrochemical characterization, and nanosecond transient absorption spectroscopy. Singlet energy transfer from the Bodipy antenna to the C_60_ module was confirmed by fluorescence quenching studies. The intersystem crossing of the latter produced the triplet excited state. The nanosecond transient absorption spectroscopy showed that the triplet state is either localized on the C_60_ module (for assembly **B-1·C-1**), or on the styryl-Bodipy antenna (for assemblies **B-2·C-1** and **B-3·C-1**). Intra-assembly forward–backward (ping-pong) singlet/triplet energy transfer was proposed. In contrast to the H-bonding assemblies, slow triplet energy transfer was observed for the non-hydrogen bonding mixtures. As a proof of concept, these supramolecular assemblies were used as triplet photosensitizers for triplet–triplet annihilation upconversion.

## Abstract 2

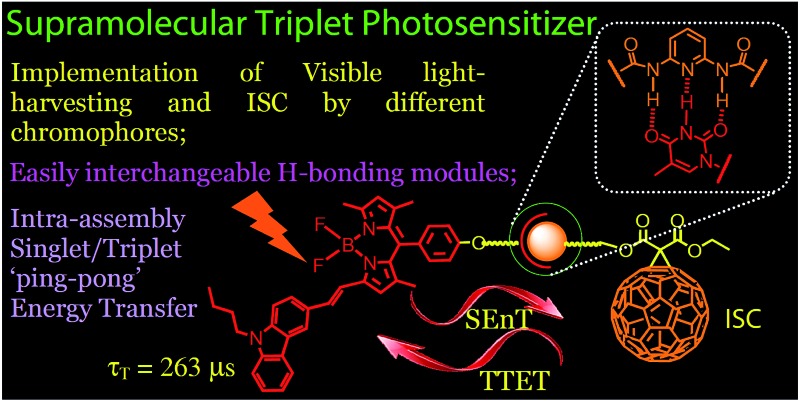
Hydrogen bonding-mediated supramolecular triplet photosensitizers with easily interchangeable visible light-harvesting Bodipy modules and the fullerene intersystem crossing module were devised.

## 1. Introduction

Triplet photosensitizers (PSs) have attracted much attention due to their applications in photocatalysis,^1^–^5^ photodynamic therapy,^6^–^8^ and more recently triplet–triplet annihilation upconversion.^9^–^12^ Conventional triplet PSs include porphyrins, halogenated xanthanes such as Rose Bengal and methylene blue, and transition metal complexes such as Pt(ii), Ru(ii) or Ir(iii) complexes, *etc.*^13^–^15^ Recently, halogenated Bodipys were developed as a new family of triplet PSs, due to their easily derivatized molecular structures, good photostability and strong absorption of visible light.^7^,^16^

There are a few challenges to be addressed in the molecular design of new triplet PSs. Firstly, most of the known triplet PSs contain heavy atoms to facilitate efficient intersystem crossing (ISC).^7^,^14^,^16^ However, for some chromophores, it is difficult to introduce heavy atoms into the molecular structure, and the heavy atom effect may become inefficient for chromophores with short S_1_ state lifetimes, or for bulky chromophores.13b,^14^ On the other hand, heavy atom-free organic triplet photosensitizers are difficult to ‘design’ because the ISC capability of such chromophores is almost unpredictable.^14^ Secondly, the known triplet PSs are based on an integrated molecular structure motif, *i.e.* the visible light-harvesting and the ISC are implemented by the same chromophore, or by covalently bonded chromophores in dyad/triad triplet PSs, for example the recently developed broadband visible light-absorbing organic triplet PSs.^7^,^14^ This approach is synthetically demanding if a library of chromophores is to be screened for the preparation of triplet PSs.^5^,^17^–^19^ Therefore, new methods are desired for the feasible preparation of organic triplet PSs.

Concerning this aspect, supramolecular assembly is particularly interesting due to the ability to fabricate molecular assemblies with different modules.^20^,^21^ For example, the supramolecular photochemistry of the hydrogen bonded C_60_–ferrocene dyad was studied.22*a* But the components showed weak absorption of visible light, thus the assembly was unsuitable for use as a triplet PS. Recently, a rotaxane was studied with fluorescence-resonance-energy-transfer (FRET), but the fluorophore present in the rotaxane was unable to produce any triplet excited states upon photoexcitation.22*b*

In order to address the above challenges in the area of triplet PSs, herein we propose using hydrogen bonded molecular assembly as a new approach for the design of organic triplet PSs.^23^–^25^ This method is also useful for designing heavy atom-free triplet PSs. One of the hydrogen bonding modules is used as a visible light-harvesting antenna and singlet energy donor, whereas the complementary H-bonding module is used as a singlet energy acceptor and a spin converter for triplet formation at the same time.^14^ The distance between the energy donor and acceptor can be controlled, and singlet energy transfer is assured. With this method, a wide variety of singlet energy donors can be feasibly screened, because the H-bonded molecular assembly is easy to obtain simply by mixing the H-bond donor and the H-bond acceptor in appropriate solvents, and the triplet formation upon photoexcitation can be feasibly evaluated by various spectroscopic methods. We confirmed this new molecular design concept with the preparation of H-bonded Bodipy–C_60_ assemblies. Efficient and fast intra-assembly singlet and triplet energy transfers were observed, based on steady state and nanosecond transient absorption spectroscopy. In contrast, much slower intermolecular triplet state energy transfer was observed for the reference modules with which no H-bonds could be formed. The application of the H-bond assemblies in singlet oxygen (^1^O_2_) photosensitization and triplet–triplet annihilation (TTA) upconversion was studied. This molecular design method will be useful for the study of organic triplet photosensitizers.

## 2. Results and discussions

### 2.1 Molecular design rationale

**Scheme 1:**
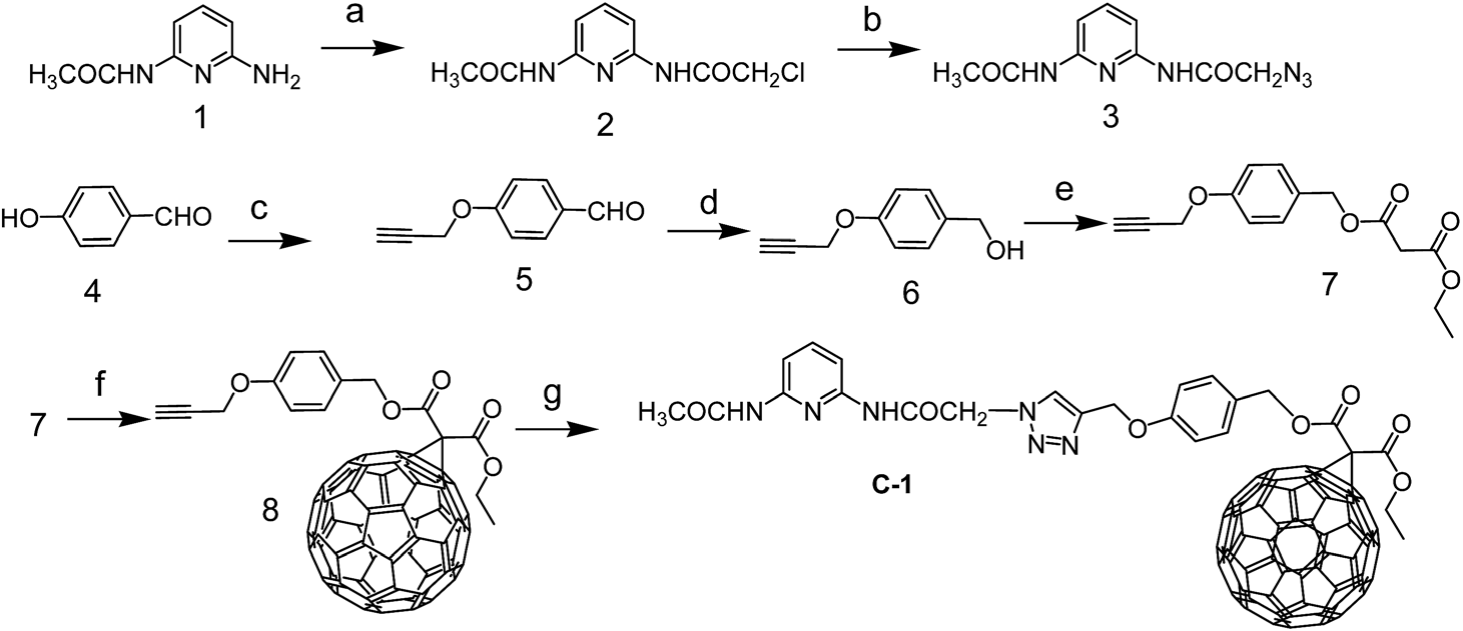
Preparation of the hydrogen bonding C_60_ module **C-1**. (a) 2-Chloro-acetyl chloride, dry CH_2_Cl_2_, Ar, 25 °C, 2 h; (b) NaN_3_, DMF, 70 °C, 5 h; (c) 3-bromo-1-propyne, potassium carbonate, ethanol, 79 °C, 6 h; (d) NaBH_4_, THF–CH_3_OH, rt, 5 min; (e) methyl malonyl chloride, CH_2_Cl_2_, Ar, 25 °C, 12 h; (f) iodine, C_60_, DBU, toluene, 10 h, Ar, 25 °C; (g) sodium ascorbate, CuSO_4_, anhydrous CHCl_3_–EtOH–water (12 : 1 : 1, v/v), rt, 12 h.

**Scheme 2:**
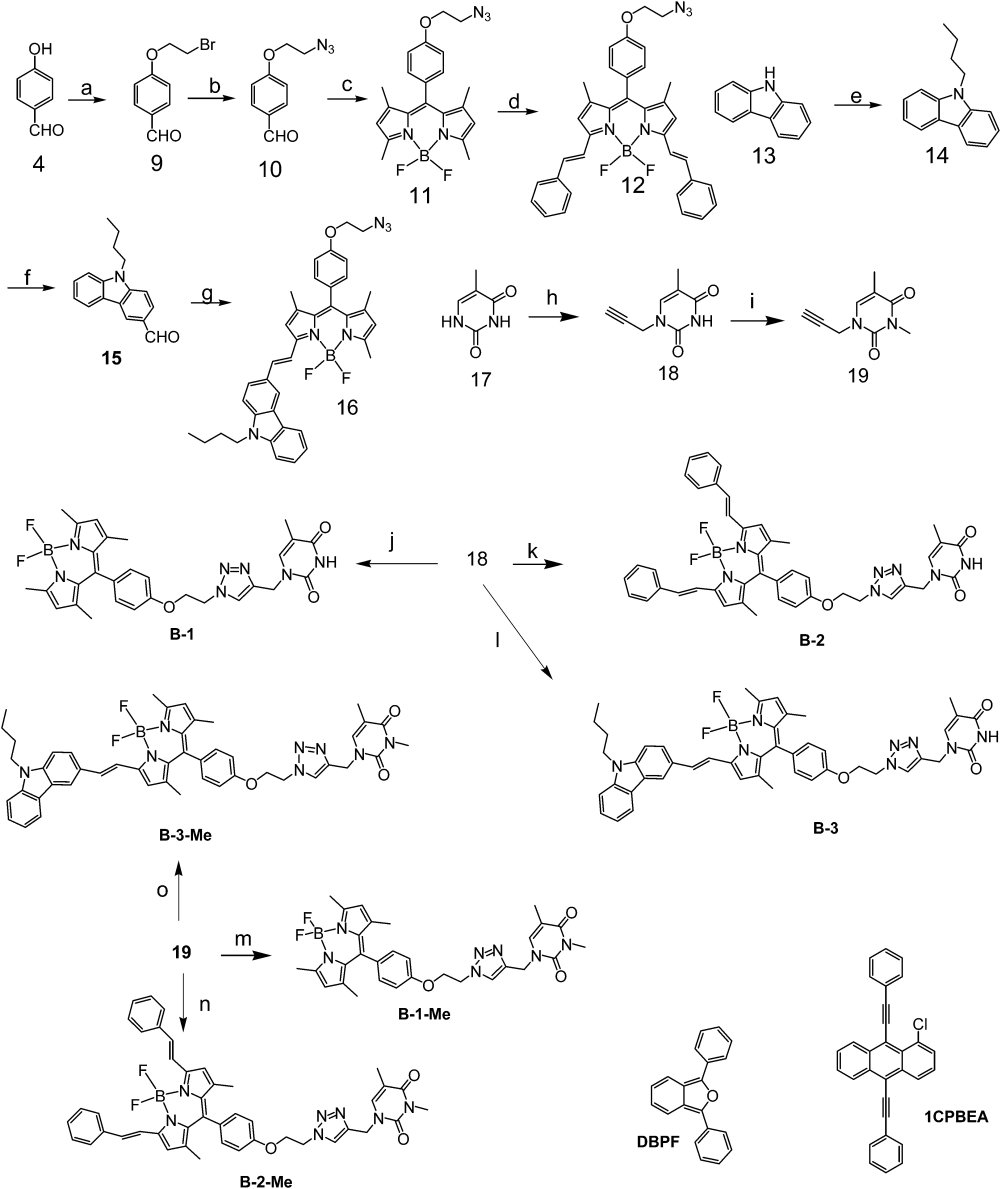
Preparation of the hydrogen bonding Bodipy modules **B-1**, **B-2**, and **B-3**, and the reference compounds which are unable to form strong hydrogen bonds with module **C-1**, *i.e.***B-1-Me**, **B-2-Me** and **B-3-Me**. The singlet oxygen (^1^O_2_) scavenger DBPF and the triplet acceptor used in TTA upconversion, 1CPBEA, were also presented. (a) Anhydrous K_2_CO_3_, 1,2-dibromoethane, dry ethanol, Ar, 70 °C; (b) NaN_3_, DMF, 100 °C, 10 h; (c) 2,4-dimethylpyrrole, TFA, anhydrous CH_2_Cl_2_, rt, 12 h; DDQ, BF_3_·Et_2_O, 12 h; (d) benzaldehyde, acetic acid, piperidine, microwave irradiation, 8 min; (e) sodium hydride, *n*-butyl bromide, DMF, rt, 2 h; (f) DMF, POCl_3_, rt, 3 h, then 60 °C, 6 h; (g) 9-butyl-9*H*-carbazole-3-carbaldehyde, acetic acid, piperidine, microwave irradiation, 5 min; (h) propargyl bromide, K_2_CO_3_, DMF, rt, Ar, 10 h; (i) NaH, methyl iodide, rt, Ar, 12 h; (j) **18**, sodium ascorbate, CuSO_4_, rt, Ar, 12 h; (k) **18**, sodium ascorbate, CuSO_4_, rt, Ar, 12 h; (l) **18**, sodium ascorbate, CuSO_4_, rt, Ar, 20 h; (m) **19**, sodium ascorbate, CuSO_4_, rt, Ar, 12 h; (n) **19**, sodium ascorbate, CuSO_4_, rt, Ar, 12 h; (o) **19**, sodium ascorbate, CuSO_4_, rt, Ar, 24 h.

**Scheme 3:**
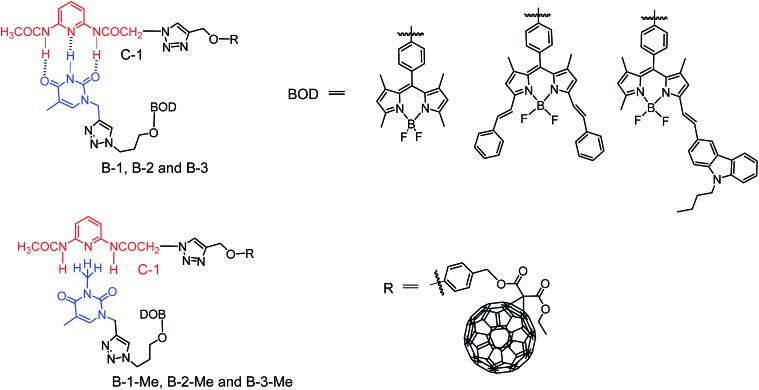
Structures of the hydrogen-bonding assemblies (**C-1**/**B-1**, **B-2** and **B-3**, upper row), and the modules which are unable to form strong hydrogen bonds (**C-1**/**B-1-Me**, **B-2-Me** and **B-3-Me**, lower row).

An *N*-acetyl-1,6-diaminopyridine-thymine based triple hydrogen bonding motif was used (Schemes 1–3). Thymine was connected to different Bodipy derivatives in order to act as the visible light-harvesting H-bonding module, *i.e.* the singlet energy donor. The *N*-acetyl-1,6-diaminopyridine unit was connected to C_60_ to form **C-1** (Scheme 1), and acted as the singlet energy acceptor and spin converter for triplet formation.22a,^26^–^29^ Note that the visible light-absorbing ability of C_60_ is very weak,^30^ but it is a good singlet energy acceptor because of the low S_1_ state energy level (1.76 eV).^31^–^37^

Bodipy was selected as the visible light-harvesting antenna, due to its satisfactory photophysical properties and easily derivatized molecular structures.^38^–^42^ The Bodipy unit in **B-1** shows absorption at *ca.* 505 nm, whereas in **B-2** and **B-3**, the styryl-Bodipy unit shows absorption at 627 nm and 592 nm, respectively (Scheme 2).^43^ H-bonded assemblies with different absorption wavelengths can be easily prepared with **B-1**, **B-2** or **B-3** as the visible light-harvesting H-bonding module, and with **C-1** as the singlet energy acceptor and spin converter (Scheme 3). In order to study the effect of H-bonding on the photophysical processes, singlet energy donors **B-1-Me**, **B-2-Me** and **B-3-Me** (Schemes 2 and 3), which are unable to form H-bonds with **C-1**, were prepared. These reference compounds contain a methyl group at the N-position of the thymine, and as a result the H-bonding with **C-1** is substantially inhibited.^27^–^29^ All the compounds were prepared using routine synthetic methods and obtained in moderate to satisfactory yields. The molecular structures were fully characterized with ^1^H NMR, ^13^C NMR and HRMS.

### 2.2 UV/vis absorption and fluorescence spectra

**Fig. 1:**
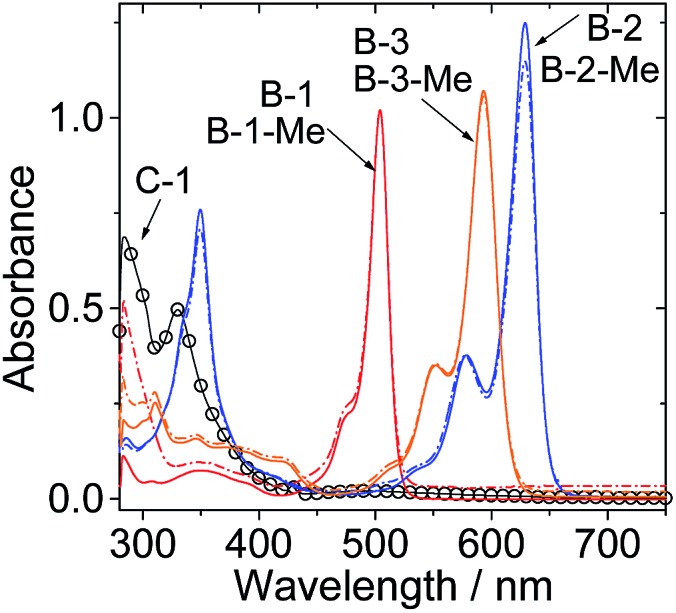
UV/vis absorption spectra of **C-1**, **B-1**, **B-1-Me**, **B-2**, **B-2-Me**, **B-3** and **B-3-Me** (note that the absorptions of the methylated Bodipy modules are identical to those of the non-methylated Bodipy modules). *c* = 1.0 × 10^–5^ M in toluene, 20 °C.

The UV/vis absorption spectra of the compounds were studied (Fig. 1 and Table 1). **C-1** shows the characteristic absorption of the C_60_ unit, which is centered at 331 nm. For the singlet energy donors, *i.e.* the Bodipy derivatives **B-1**, **B-2** and **B-3**, the absorption bands are located at 505 nm, 630 nm and 593 nm, respectively. The S_1_ state energy level of C_60_ is 1.72 eV,^30^,^34^ which is lower than those of the visible light-harvesting energy donors (**B-1**, **B-2** and **B-3**). The methylated visible light-harvesting compounds **B-1-Me**, **B-2-Me** and **B-3-Me** show similar absorption spectra to their analogues without methyl substitution on the N atom of thymine.

**Table 1:** Photophysical parameters of the Bodipy and C_60_ hydrogen bonding modules^*a*^

	*λ*_abs_	*ε* ^*b*^	*λ*_em_	*Φ*_F_ (%)	*τ*_F_ ^*f*^ (ns)
**B-1**	505	10.2	517	75.7^*c*^	3.5
**B-1-Me**	505	10.2	517	64.0^*c*^	3.6
**B-2**	630	12.4	642	73.5^*d*^	5.0
**B-2-Me**	630	11.5	642	65.4^*d*^	4.9
**B-3**	593	10.6	611	70.3^*e*^	3.7
**B-3-Me**	593	10.4	611	61.2^*e*^	3.9
**C-1**	331	5.1	—^*g*^	—^*g*^	—^*g*^

^*a*^In toluene (1.0 × 10^–5^ M).

^*b*^Molar extinction coefficient at the absorption maximum (10^4^ M^–1^ cm^–1^).

^*c*^With 2,6-diiodo-4,4-difluoro-1,3,5,7-tetramethyl-8-phenyl-4-bora-3*a*,4*a*-diaza-*s*-indacene as the standard (*Φ* = 71.2% in acetonitrile).

^*d*^With carbazole-styryl-Bodipy as the standard (*Φ* = 72.0% in toluene).

^*e*^With carbazole-styryl-Bodipy as the standard (*Φ* = 63.0% in acetonitrile).

^*f*^Fluorescence lifetimes.

^*g*^Not determined.

**Fig. 2:**
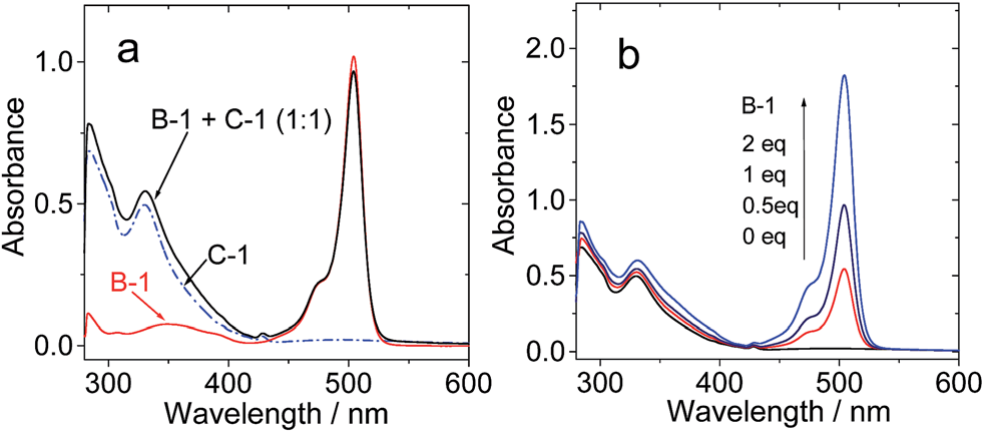
UV/vis absorption spectra of (a) **B-1**, **C-1** and **C-1**/**B-1** (1/1); (b) **C-1** (1.0 × 10^–5^ M) with increasing concentrations of **B-1**. *c* = 1.0 × 10^–5^ M in toluene, 20 °C.

The UV/vis absorption in toluene of **C-1**/**B-1**, in which H-bonds have presumably been formed, is the sum of the UV/vis absorptions of **B-1** and **C-1** (Fig. 2a). This result indicated that H-bonding does not induce any interaction between the Bodipy and C_60_ moieties in the ground state.^34^ Similar results were observed for the mixture of **B-1-Me** and **C-1**, in which no H-bonding is expected (see ESI, Fig. S45†).^27^ This conclusion was supported by the titration of **C-1** with higher **B-1** concentrations (2 eq. *vs.***C-1**, Fig. 2b).

**Fig. 3:**
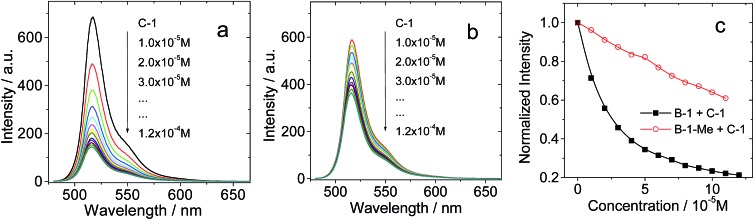
Quenching of the fluorescence of (a) **B-1** and (b) **B-1-Me** by **C-1**. *c* = 1.0 × 10^–5^ M in toluene (*λ*_ex_ = 475 nm). (c) Emission intensity of **B-1** and **B-1-Me** at 517 nm (*c* = 1.0 × 10^–5^ M in toluene) *versus* concentration of **C-1**, following excitation at 475 nm at 20 °C.

In order to verify the H-bonding between the Bodipy visible light-harvesting unit and **C-1**, fluorescent titration of **B-1** with **C-1** was carried out (Fig. 3). The rationale is that the fluorescence of **B-1** will be quenched due to energy transfer (such as through space energy transfer) to C_60_ if H-bonds are formed with **C-1**.^24^,^27^,^29^,42b **B-1** alone shows intense fluorescence at 517 nm (quantum yield *Φ*_F_ = 75.7%). Upon addition of **C-1** into a solution of **B-1**, however, the fluorescence intensity of **B-1** was substantially quenched (Fig. 3a), and saturation was established after addition of 12 eq. **C-1**. The fluorescence intensity was reduced by 80%. The formation of hydrogen bonds between **C-1** and **B-1** is an equilibrium process. In order for most of the **B-1** molecules to be H-bonded, *i.e.* in order to fully quench the fluorescence of **B-1**, excess **C-1** has to be used. Interestingly, for **B-1-Me**, which is unable to form strong H-bonds with **C-1** due to the methyl group on the N atom of the thymine moiety, the fluorescence was quenched to a much lesser extent, by 40% on addition of the same amount of **C-1** (Fig. 3b). Therefore, we propose that strong H-bonds formed for **C-1**/**B-1**, but not for **C-1**/**B-1-Me**.^27^

Similar fluorescence quenching profiles were observed for **B-2** and **B-3** (see ESI, Fig. S51 and S53†). The fluorescence emission wavelength of **B-3** is much longer than that of **B-1**. It should be pointed out that the S_1_ state energy level of **B-3** (approximated as 2.04 eV) is still higher than that of **C-1** (1.76 eV), thus intra-assembly singlet energy transfer is assured. For **B-3-Me**, no H-bonds can be formed, thus the quenching occurs to a much lesser extent (see ESI, Fig. S53b†).

The fluorescence lifetime of **B-1** (or **B-2**, or **B-3**) was measured upon titration with **C-1** (see ESI, Fig. S68†). The fluorescence lifetime is nearly constant (3.5–3.4 ns), despite the quenching of the fluorescence intensity with increasing **C-1** concentration. Thus, we conclude that the fluorescence quenching is due to a mechanism similar to static quenching, not dynamic quenching,^44^,^45^ upon formation of the hydrogen bonds. That is, the fluorescence of the H-bonded assembly is completely quenched, while the un-bonded Bodipy fluorophore shows residual fluorescence.^44^ In other words, the H-bonding assembly becomes non-fluorescent, and the residual fluorescence observed during the titration is due to the free Bodipy modules in the solution. In order to verify that the fluorescence quenching during the titration is due to H-bond mediated assembly, hexafluoroisopropanol was added in order to disrupt the H-bonds. As a result, fluorescence recovery was observed (see ESI, Fig. S54†), which indicated that the fluorescence quenching was due to the formation of a molecular assembly *via* H-bonding.

These studies, using the visible light-harvesting chromophores **B-1**, **B-2** and **B-3** to form molecular assemblies with **C-1**, demonstrate the advantage of using H-bonding to induce intra-assembly energy transfer, that is, different combinations can be easily accessed and screened for efficient intramolecular energy transfer and formation of triplet states.

The stability constants of the visible light-harvesting Bodipys and **C-1** were calculated based on the fluorescence titration experiments (Table 2).^46^,^47^ The stability constants for the H-bond forming combinations show that the bonding is strong. For the methylated Bodipy derivatives, however, the stability constants are 10-fold smaller.

**Table 2:** The stability constants for the interactions of the Bodipy H-bonding modules with the **C-1** H-bonding module

	**B-1**	**B-1-Me**	**B-2**	**B-2-Me**	**B-3**	**B-3-Me**
*K*_SV_ ^*a*^/M^–1^	4.08 × 10^4^	3.02 × 10^3^	3.67 × 10^4^	1.22 × 10^3^	5.14 × 10^4^	5.43 × 10^3^
*K*_b_ ^*b*^/M^–1^	3.6 × 10^5^	—^*c*^	1.38 × 10^6^	—^*c*^	1.15 × 10^6^	—^*c*^

^*a*^ *K*_SV_ is the linear Stern–Volmer quenching constant.

^*b*^ *K*_b_ represents the intrinsic binding constant of the compound with **C-1**.

^*c*^Not applicable.

### 2.3 Electrochemical studies and the free energy changes of the intramolecular electron transfers

**Fig. 4:**
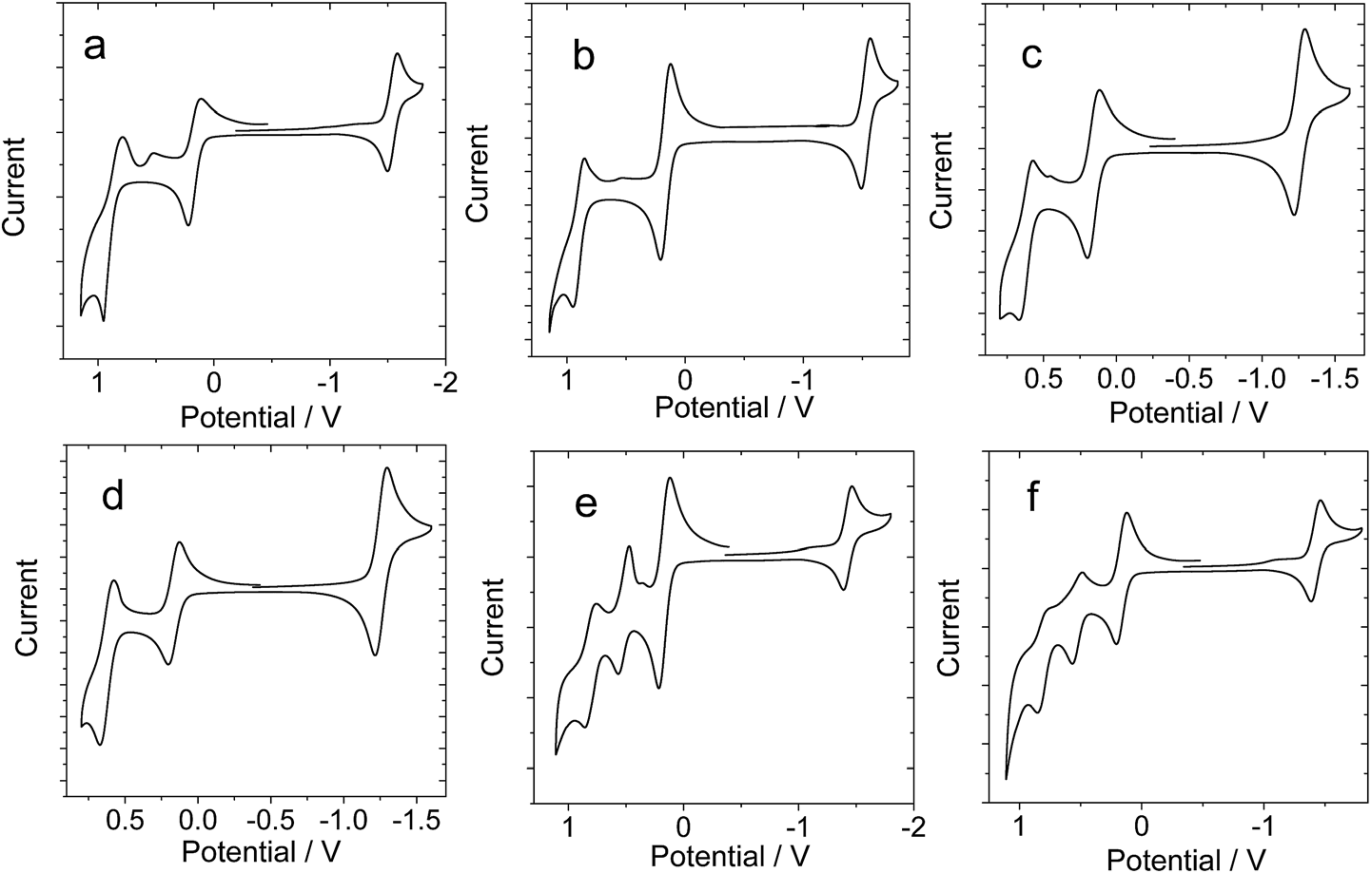
Cyclic voltammograms of the Bodipy H-bonding modules. Ferrocene (Fc) was used as an internal reference (*E*_1/2_ = +0.38 V (Fc^+^/Fc) *vs.* SCE). (a) **B-1**, (b) **B-1-Me**, (c) **B-2**, (d) **B-2-Me**, (e) **B-3**, and (f) **B-3-Me**. Experiments were carried out in deaerated DCM solutions containing 0.5 mM photosensitizer, alone or with ferrocene, 0.10 M Bu_4_NPF_6_ as the supporting electrolyte, and a Ag/AgNO_3_ reference electrode; scan rate: 0.05 V s^–1^.

**Fig. 5:**
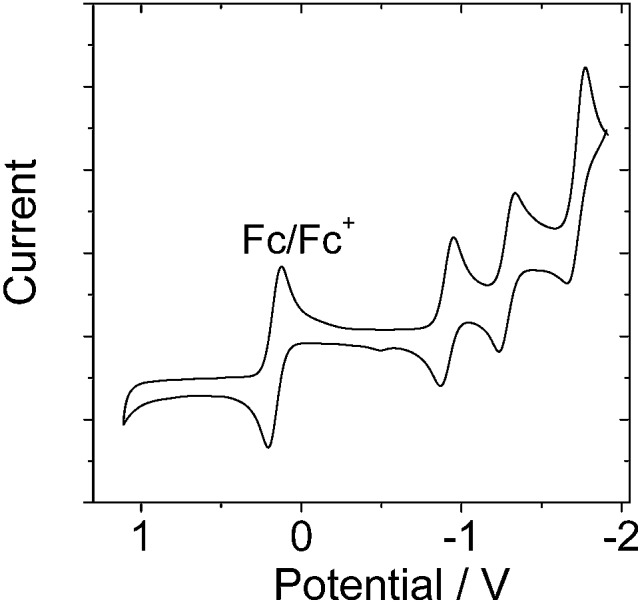
Cyclic voltammogram of **C-1**. Ferrocene (Fc) was used as an internal reference (*E*_1/2_ = +0.38 V (Fc^+^/Fc) *vs.* SCE). Experiments were carried out in deaerated DCM solutions containing 0.5 mM **C-1** alone, 0.10 M Bu_4_NPF_6_ as the supporting electrolyte, and a Ag/AgNO_3_ reference electrode; scan rate: 0.05 V s^–1^.

In order to study the intra-assembly photoinduced electron transfer, the electrochemical properties of the Bodipy and C_60_ modules were studied using cyclic voltammetry (Fig. 4).^34^,^48^,^49^ For **B-1**, a reversible reduction wave was observed at –1.32 V. A pseudo-reversible oxidation wave was observed at +1.11 V. Similar redox potentials were observed for **B-1-Me**. For **B-2**, a pseudo-reversible oxidation wave was observed at +0.84 V, which was shifted anodically as compared with **B-1**. For the carbazole-containing **B-3**, two reversible oxidation waves at +0.74 and +1.03 V were observed, which were different from **B-1** and **B-2**. The two reversible reduction waves of **B-3** and **B-3-Me** are due to the carbazole-styryl-Bodipy moiety, which was confirmed by studying a reference compound (see ESI, Fig. S67†). For the C_60_ module, *i.e.***C-1**, two reversible reduction waves at –0.7 V and –1.08 V were observed (Fig. 5), as well as a pseudo-reversible reduction wave at –1.53 V. These reduction waves are attributed to the C_60_ moiety.

The plausible photoinduced intra-assembly electron transfer between the Bodipy and the C_60_ module can be evaluated with the Gibbs free energy changes, which are calculated with the Rehm–Weller equation,^34^,^48^,^49^
1
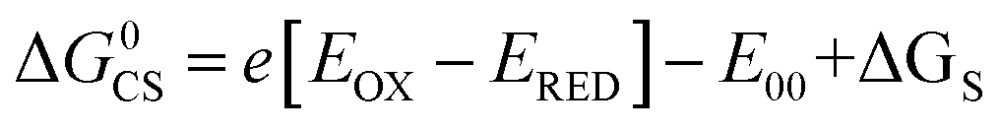

2
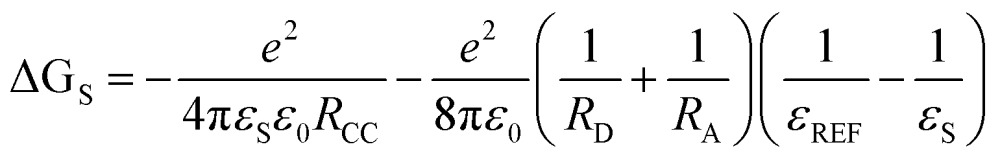
where Δ*G*_S_ is the static Coulombic energy, which is described by eqn (2). *e* is the electronic charge, *E*_OX_ is the half-wave potential for one-electron oxidation of the electron-donor unit, *E*_RED_ = half-wave potential for one-electron reduction of the electron acceptor unit, *E*_00_ is the approximate energy level obtained from the fluorescence emission (for the singlet excited state), *ε*_S_ = static dielectric constant of the solvent, and *R*_CC_ = center-to-center separation distance between the electron donor (Bodipy module) and the electron acceptor (**C-1** module). As determined by DFT optimization of the geometries, *R*_CC_ (**B-1·C-1**) = 40.0 Å, *R*_CC_ (**B-2·C-1**) = 43.9 Å and *R*_CC_ (**B-1·C-1**) = 49.5 Å. *R*_D_ is the radius of the electron donor, *R*_A_ is the radius of the electron acceptor, *ε*_REF_ is the static dielectric constant of the solvent used for the electrochemical studies, and *ε*_0_ is the permittivity of free space. The solvents used in the calculation of the free energy of the electron transfer are toluene (*ε*_S_ = 2.4) and CH_2_Cl_2_ (*ε*_S_ = 8.93). The electrochemical data and the free energy changes of the photoinduced electron transfers in the H-bonding assemblies are summarized in Table 3. The results show that intra-assembly photoinduced electron transfer is thermodynamically prohibited. Therefore, the fluorescence quenching effect observed during the H-bonding titration (Fig. 3) is due to intra-assembly energy transfer, not electron transfer.

**Table 3:** Redox potentials of the H-bond modules and the free energy changes (Δ*G*_CS_) for the intramolecular electron transfers (with the Bodipy unit as electron donor and the C_60_ unit as electron acceptor). Anodic and cathodic peak potentials are presented. The potential values of the compounds are *vs.* Ag/AgNO_3_ reference electrode, with Fc as internal reference (*E*_1/2_(Fc^+^/Fc) = +0.38 V)^*a*^

	*E*_OX_ (V)	*E*_RED_ (V)	Δ*G*_CS_ (eV)
**B-1**	+1.11	–1.32	+0.21,^*b*^ 1.17^*c*^
**B-1-Me**	+1.12	–1.31	+0.25,^*b*^ 1.33^*c*^
**B-2**	+0.84	–1.04	+0.28,^*b*^ 0.92^*c*^
**B-2-Me**	+0.84	–1.04	+0.32,^*b*^ 1.06^*c*^
**B-3**	+0.74/+1.03	–1.21	+0.36,^*b*^ 0.97^*c*^
**B-3-Me**	+0.74/+1.03	–1.22	+0.40,^*b*^ 1.14^*c*^
**C-1**	—^*d*^	–0.70/–1.08/–1.53	—^*e*^

^*a*^Cyclic voltammetry carried out in Ar saturated acetonitrile containing 0.10 M Bu_4_NPF_6_ supporting electrolyte; counter electrode was a Pt electrode; working electrode was a glassy carbon electrode; the Ag/AgNO_3_ couple was used as the reference electrode. *c*[Ag^+^] = 0.1 M. Conditions: 0.5 mM dyad photosensitizers and 0.5 mM ferrocene in CH_2_Cl_2_, 293 K.

^*b*^With reference **C-1** as electron acceptor, in CH_2_Cl_2_.

^*c*^With reference **C-1** as electron acceptor, in toluene.

^*d*^No reduction potentials were observed.

^*e*^No Δ*G*_cs_ values were calculated.

### 2.4 Nanosecond transient absorption spectroscopy: intra-assembly and intermolecular triplet state energy transfer

The formation of H-bonds between the Bodipy visible light-harvesting chromophores and the fullerene module **C-1** will induce singlet energy transfer from the Bodipy part to the C_60_ part.^34^,^49^ As a result, a triplet excited state will be produced *via* the inherent ISC of C_60_.^35^–^37^ Thus, nanosecond transient absorption spectroscopy was used to confirm the production of the triplet state *via* H-bonding mediated intra-assembly energy transfer.22*a* Indeed, we observed the triplet state of **C-1** upon photoexcitation (no signals could be detected in aerated solution, see ESI, Fig. S65†).

**Fig. 6:**
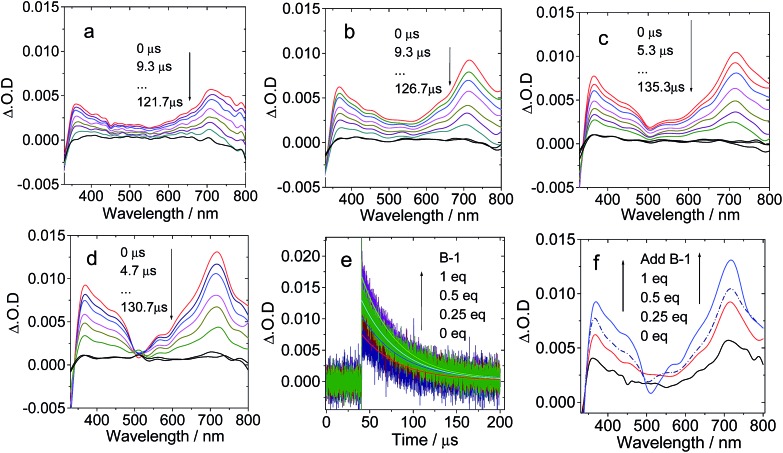
Nanosecond transient absorption spectra of **C-1** on addition of increasing amounts of compound **B-1**. The transient signal intensified with increasing **B-1** concentration: (a) 0 eq. **B-1**; (b) 0.25 eq. **B-1**; (c) 0.5 eq. **B-1**; and (d) 1 eq. **B-1**. (e) Decay trace at 720 nm; (f) comparison of the transient absorption spectra at different **B-1** concentrations with delay time of 0 μs. *c* = 1.0 × 10^–5^ M; spectra were recorded in toluene after pulsed excitation at 495 nm under N_2_ at 20 °C.

First, **C-1** was titrated with **B-1** (Fig. 6). The mixed solution was photoexcited at 495 nm with a nanosecond pulsed laser. The absorption of **C-1** at 495 nm is weak. Thus, selective excitation of **B-1** in the mixture is possible. On increasing the concentration of **B-1**, the excited state absorption (ESA) of the C_60_ part at 715 nm was enhanced (indicated by the ΔO.D. values, which are proportional to the population of the transient species, Fig. 6f), indicating enhanced singlet energy transfer and finally the production of the triplet state with increasing concentration of **B-1** (Fig. 6d). The presence of the triplet state was confirmed by measurement of the transient signal in aerated solution (no signal was observed, see ESI, Fig. S65 and S66†). It should be pointed out that the triplet excited state is localized on the C_60_ moiety, not on the Bodipy moiety, because the ground state bleaching band at 505 nm (at which the Bodipy unit shows steady state absorption) was not observed. This is in agreement with our previous studies of C_60_–Bodipy dyads.^35^ The triplet state lifetimes are in the range of 40.9–44.2 μs, which supports the localization of the triplet state on the C_60_ moiety.^30^ In comparison, excitation of **B-1** alone didn’t produce any triplet state (see ESI, Fig. S62a†); thus, the cascade singlet energy transfer and ISC only occur in the **B-1·C-1** assembly.

The triplet state quantum yields of **C-1** and the **B-1·C-1** hydrogen bonding assembly (1 : 1) were determined as 86.1% and 67.5%, respectively, using the triplet energy transfer method, β-carotene as triplet energy acceptor, and Ru(bpy)_3_Cl_2_ as a reference (see Experimental section for details).16b,c

The triplet state of **C-1** upon titration with **B-1-Me** was also studied (see ESI, Fig. S56†). In this situation, no strong H-bonding is expected.^27^ Intermolecular singlet energy transfer is unlikely for two compounds mechanically mixed in solution (the distance between the energy donor and the energy acceptor can be treated as infinite, well exceeding the distance required for the Förster energy transfer). We did not observe any increase in the transient absorption of the T_1_ state of C_60_ on increasing the concentration of **B-1-Me** (see ESI, Fig. S56†). Thus, the intra-assembly singlet energy transfer was confirmed by studying this reference compound.

Previously, we demonstrated that for a Bodipy–C_60_ covalent dyad, intramolecular ping-pong energy transfer is possible, *i.e.* forward singlet energy transfer to the C_60_ unit, and backward triplet energy transfer to the styryl-Bodipy visible light-harvesting chromophore.^36^,^37^ Herein, we studied this photophysical process in the H-bonding mediated supramolecular assemblies. To this end, **B-1** is not a good candidate because the T_1_ state energy level of C_60_ is lower than that of the Bodipy unit in **B-1**, therefore no backward triplet state energy transfer is expected, because the upward triplet energy transfer from C_60_ to the Bodipy unit in **B-1** is thermodynamically prohibited. Instead, we selected **B-3** for this study, because we have shown that the T_1_ state of the styryl-Bodipy–C_60_ dyad/triad is localized on the styryl-Bodipy moiety, not on the C_60_ unit. That is, the styryl-Bodipy antenna has a lower T_1_ state energy level than C_60_.

**Fig. 7:**
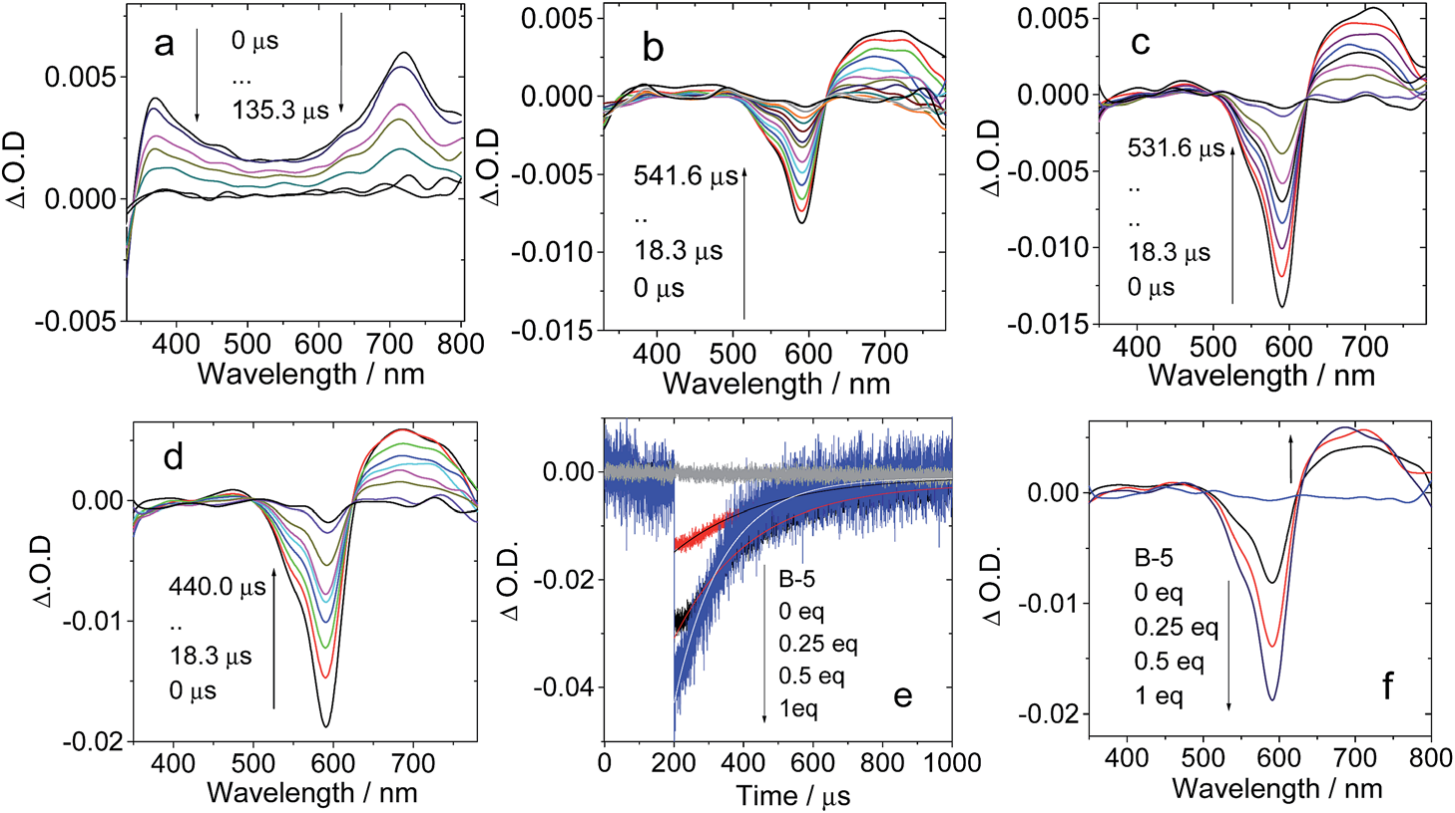
Nanosecond transient absorption spectra of the **C-1** H-bonding module upon titration with the complementary **B-3** H-bonding module. Transient absorption of **C-1** in the presence of (a) 0 eq. **B-3**, (b) 0.25 eq. **B-3**, (c) 0.5 eq. **B-3**, and (d) 1 eq. **B-3**. (e) Decay trace at 593 nm; (f) transient spectra extracted from (a)–(d) at delay times of 0 μs. *c* = 1.0 × 10^–5^ M; spectra were recorded in toluene after pulsed excitation at 590 nm under N_2_ at 20 °C.

For **B-3**, the excitation wavelength is 590 nm, at which the C_60_ unit shows weak absorption. On addition of **B-3** into a solution of **C-1**, a bleaching band at 590 nm was observed, which could be assigned to the depletion of the ground state of the carbazole-styryl-Bodipy in **B-3** (Fig. 7b–d). ESA of the T_1_ state (T_1_ → T_*n*_ transitions) of the styryl-Bodipy antenna was observed in the range of 600–750 nm.^35^ Thus, we propose that ping-pong energy transfer occurs in **B-3·C-1**. First, **B-3** is photoexcited with a 590 nm laser, and then intra-assembly singlet energy transfer from the carbazole-styryl-Bodipy to the H-bonded C_60_ moiety occurs. The ISC of C_60_ produces a triplet excited state, followed by the backward triplet energy transfer from C_60_ to the carbazole-styryl-Bodipy module; as a result, the ground state bleaching band and the ESA of the styryl-Bodipy part in **B-3** were observed.

With increasing **B-3** concentration, the ΔO.D. magnitude of **B-3** increased, indicating enhanced visible light-harvesting, ping-pong singlet–triplet energy transfer in the assembly, and finally the production of the triplet excited state of the antenna (Fig. 7f). When 0.25 eq. to 1 eq. **B-3** was added, the triplet state lifetime decreased from 263.2 μs to 138.9 μs. The self-quenching constant is 4.6 × 10^5^ M^–1^ s^–1^. This quenching effect is due to the TTA process, which is significant for transient species with long triplet state lifetimes. Similar fast intra-assembly triplet–triplet electron transfer (TTET) was observed for **B-2·C-1** (see ESI, Fig. S58†).

**Fig. 8:**
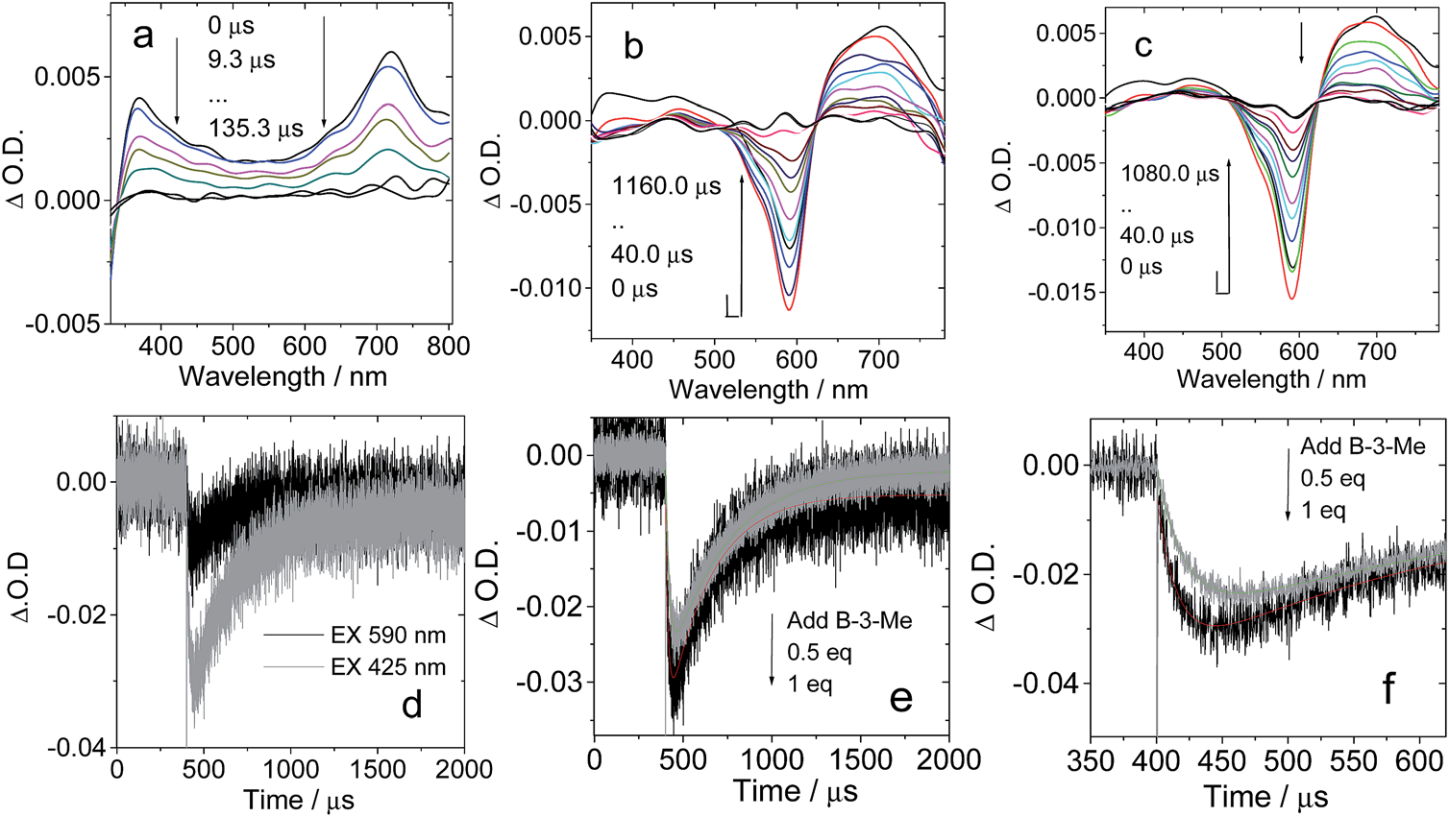
Nanosecond transient absorption spectra of H-bonding module **C-1** upon titration with the non-preferred H-bonding module **B-3-Me** (no strong H-bonds can be formed with **C-1**). Transient absorption spectra of **C-1** in the presence of (a) 0 eq. **B-3-Me**, (b) 0.5 eq. **B-3-Me** and (c) 1 eq. **B-3-Me**. (d) Decay traces at 630 nm on addition of **B-3-Me** with pulsed excitation at 425 nm (selective excitation of the C_60_ moiety) and 590 nm (selective excitation of the carbazole-styryl-Bodipy moiety). (e) Decay traces at 630 nm on addition of 0.5 eq. and 1 eq. **B-3-Me**. *c* = 1.0 × 10^–5^ M for **C-1** in toluene after pulsed excitation at 425 nm under N_2_ at 20 °C (note that for the sake of signal intensity, the excitation is at 425 nm instead of 590 nm, which was used for Fig. 7). (f) Magnification of the decay traces in (e).

No strong H-bonds should be formed in **B-3-Me**/**C-1**. The nanosecond transient absorption spectroscopy of **B-3-Me** in the presence of **C-1** was also studied (Fig. 8). Drastically different results were observed, as compared with **B-3**/**C-1**. The bleaching band of the carbazole-styryl-Bodipy moiety was observed at 593 nm (Fig. 8b). Since C_60_ was excited at 425 nm, and excitation of the solution of **B-3-Me** alone did not produce any triplet excited state (see ESI, Fig. S64b†), the population of the T_1_ state of the styryl-Bodipy moiety in **B-3-Me** is attributed to *intermolecular* triplet energy transfer, with C_60_ acting as the triplet energy donor.^50^

The evidence for this *intermolecular* energy transfer, rather than a fast *intra-assembly* energy transfer, is provided by the biphasic features of the decay traces of the transient absorption monitored at 630 nm (Fig. 8e and f), in which the first phase of the increase in intensity of the bleaching band at 593 nm is due to the intermolecular triplet energy transfer from the C_60_ moiety to the styryl-Bodipy moiety in **B-3-Me**, which takes *ca.* 15 μs. This process is the relatively slow intermolecular TTET as compared to the fast intra-assembly TTET (Fig. 7e), for which the TTET cannot be resolved with the transient absorption spectrometer (resolution of the spectrometer is 10 ns). The later recovery phase is due to decay of the T_1_ state of the styryl-Bodipy moiety. The lack of formation of an H-bonded **B-3-Me**–**C-1** molecular assembly was confirmed by the ΔO.D. values of the transient absorption upon 425 nm and 590 nm photoexcitation (Fig. 8d). The ΔO.D. value of the transient absorption upon excitation at 590 nm (where the styryl-Bodipy antenna gives strong absorption) is less than that observed with 425 nm excitation, and no slow increase in the intensity of the bleaching band was observed upon 590 nm photoexcitation. This result confirmed that there is no significant formation of a H-bond mediated molecular assembly.

For **B-2-Me**, a slow evolution of the transient bleaching was also observed (see ESI, Fig. S61†). The triplet state energy transfer takes about 16 μs. This slow process is in stark contrast to that observed for the H-bonding assemblies of **B-2·C-1** and **B-3·C-1**, for which no slowly increasing phases in the transient signals were observed. Thus, the intra-assembly (in other words the intramolecular) triplet energy transfer occurs with rate constant *k*_TTET_ > 10^8^ s^–1^ (the time-resolution of the spectrometer is 10 ns). This is an interesting result, since for the **C-1·B-2** and **C-1·B-3** assemblies, the distance between the C_60_ and the styryl-Bodipy part is *ca.* 5.0 nm, and the linker contains a saturated bond, yet the triplet energy transfer is fast.^33^,^51^ We propose movement of the Bodipy module and the C_60_ module; thus intramolecular collision causes the fast intra-assembly triplet state energy transfer to occur.

Previously, intra-assembly triplet energy transfer from C_60_ to ferrocene was observed in a hydrogen bonded C_60_ ferrocene dyad (*k* = 9.2 × 10^5^ s^–1^).22*a* A steroid-linked norbornadiene–carbazole dyad showed intramolecular triplet energy transfer with a rate constant of 3.3 × 10^5^ s^–1^ (from the carbazole to the norbornadiene moiety).^52^ For the hydrogen bonded assemblies **B-2·C-1** and **B-3·C-1**, the intramolecular triplet energy transfer from the C_60_ moiety to the styryl-Bodipy moiety is much faster (*k* > 10^8^ s^–1^). Triplet energy transfer is very often believed to occur *via* the Dexter electron exchange mechanism, which requires close contact between the energy donor and the energy acceptor. The triplet energy transfer will be negligible if the distance between the energy donor and acceptor exceeds the sum of their van der Waals radii. We propose that the flexible chains in the **B-2·C-1** and **B-3·C-1** assemblies allow direct contact of the styryl-Bodipy and the C_60_ moiety through foldamers; thus the triplet energy transfer is greatly accelerated. Previously, hydrogen bonding porphyrin–zinc assemblies were studied, and 50–80 times faster singlet–singlet energy transfer was observed for the assemblies with flexible linkers, as compared to those with rigid linkers, such as steroids.^53^

### 2.5 Molecular dynamics (MD) simulations: explanation of the fast intramolecular triplet energy transfer

**Fig. 9:**
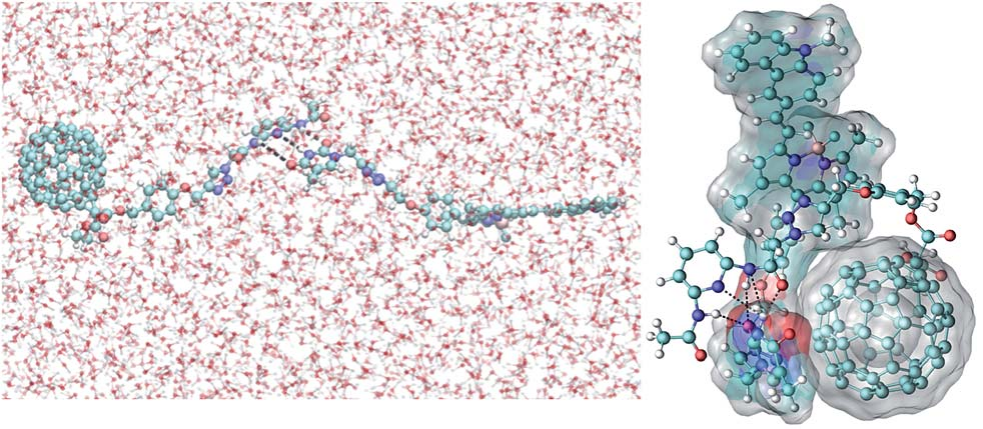
Right: conformation of the **B-3·C-1** complex in aqueous solution obtained from an MD simulation at 6.9 ns, showing the hydrogen bonds (<3 Å as indicated using black dotted lines) between **B-3** and the **C-1** motif. Left: the starting extended conformation used in the MD simulations. The white surfaces represent the solvent (water molecules) accessible surface areas of **B-3** and the fullerene motif of the **C-1** structure.

The possible conformations of the complexes containing a hydrogen acceptor and donor were explored by molecular dynamics (MD) simulations performed in aqueous solution (see ESI†).^53^ It was found that a relatively stable complex can be formed in less than 10 ns, indicating that recognition of the hydrogen donor and acceptor in aqueous solution is quite feasible. Note that the three pairs of hydrogen bonds in the initial conformation are largely preserved in the compact conformation, although **B-3** prefers to assume an extended conformation in solution. Weak π–π interactions between part of the **B-3** structure and the fullerene motifs of the **C-1** molecules may further contribute to the stability of this complex. These results indicate that fast intra-assembly TTET is possible, which can be used to rationalize the nanosecond transient absorption spectra of the **B-3·C-1** H-bond assembly (Fig. 9).

### 2.6 Hydrogen bonded Bodipy–C_60_ assemblies as singlet oxygen (^1^O_2_) photosensitizers

**Fig. 10:**
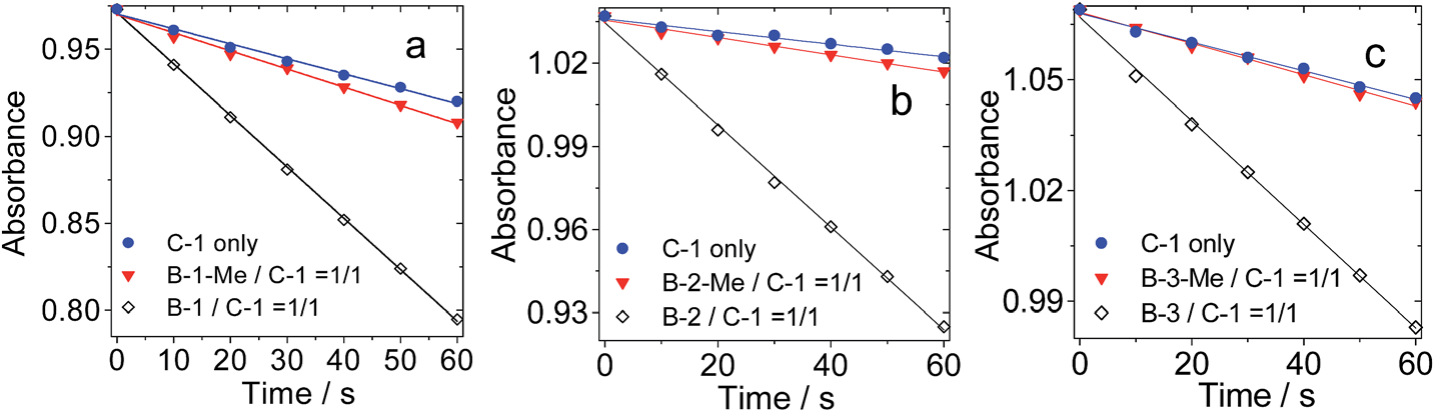
Singlet oxygen (^1^O_2_) generation by photoexcitation of **C-1**, **B-1**, **B-1-Me**, **B-2**, **B-2-Me**, **B-3** and **B-3-Me**. Hydrogen bonded pairs produced ^1^O_2_ more efficiently. Decrease in absorbance of DPBF *vs.* photoirradiation time in the presence of photosensitizers: (a) *λ*_ex_ = 495 nm, 1.1 mW m^–2^; (b) *λ*_ex_ = 630 nm, 1.3 mW m^–2^; (c) *λ*_ex_ = 588 nm, 1.3 mW m^–2^; *c* = 1.0 × 10^–5^ M in toluene, 20 °C.

The production of triplet excited states by the hydrogen bonded C_60_–Bodipy assemblies was also studied using singlet oxygen (^1^O_2_) photosensitization (Fig. 10). 1,3-Diphenylbenzofuran (DPBF) was used as ^1^O_2_ scavenger. The production of ^1^O_2_ could be monitored by following the absorbance changes of DPBF at 414 nm.

For all the hydrogen bond assemblies, *i.e.***B-1·C-1**, **B-2·C-1** and **B-3·C-1**, the ^1^O_2_ photosensitizing ability is higher than the combinations for which strong hydrogen bonds are unable to form, *i.e.***B-1-Me·C-1**, **B-2-Me·C-2**, and **B-3-Me·C-1**. These pairs show similar ^1^O_2_ photosensitizing ability to **C-1** alone. These results clearly demonstrate the effect of hydrogen bonding on intra-assembly singlet energy transfer.

### 2.7 Application in triplet–triplet annihilation upconversion

**Fig. 11:**
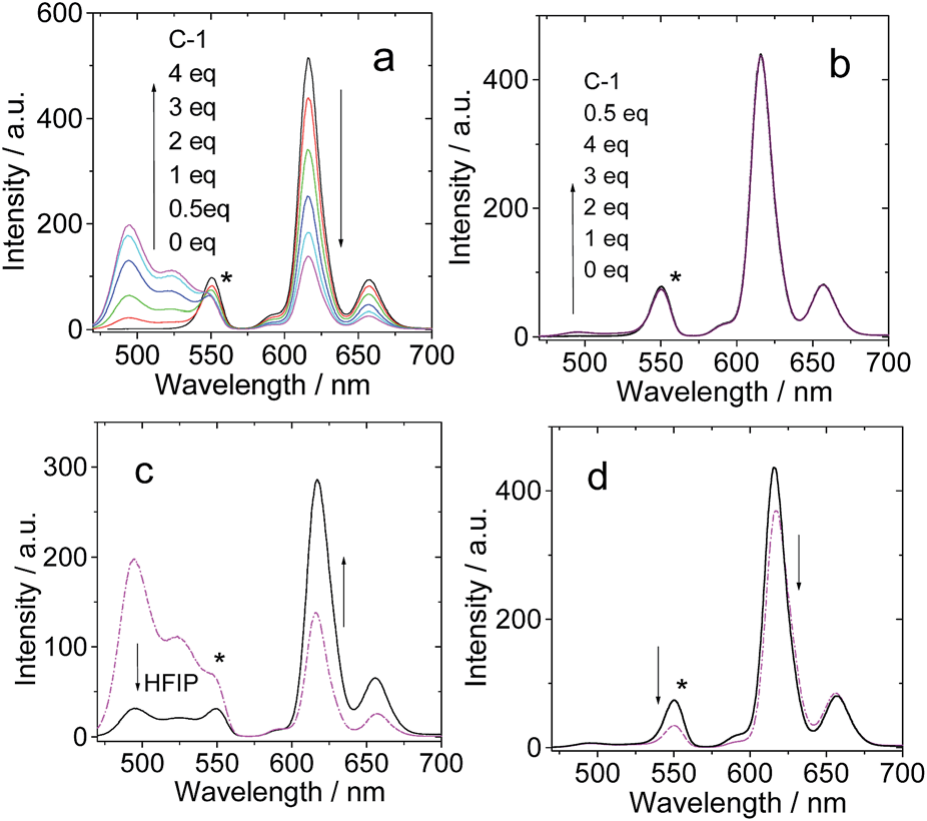
TTA upconversion of **C-1** with **B-3** and **B-3-Me** H-bonding modules. **1-CBPEA** was used as triplet acceptor. (a) Emission of **B-3** and **1-CBPEA** with increasing **C-1** concentration; (b) emission of **B-3-Me** and **1-CBPEA** with increasing **C-1** concentration; (c) emission observed when 0 μL and 100 μL of hexafluoroisopropanol (HFIP) were added to a mixed solution of **B-3**, **1-CBPEA** and 4 eq. **C-1** to disrupt the H-bonding; (d) emission observed when 0 μL and 100 μL of HFIP were added to a mixed solution of **B-3-Me**, **1-CBPEA** and 4 eq. **C-1**. Excitation was carried out with a 589 nm laser (4.4 mW, power density is 60 mW cm^–2^), and a band-pass filter (470–570 nm) was used to suppress the scattered laser. The asterisks indicate the residual emission of the antenna. *c*[photosensitizers] = 1.0 × 10^–5^ M. *c*[**1-CBPEA**] = 1.2 × 10^–4^ M in deaerated toluene, 20 °C.

Up until now, no supramolecular triplet photosensitizers for TTA upconversion have been reported. The H-bonding assemblies were used as supramolecular triplet photosensitizers for TTA upconversion, with 1-chloro-9,10-bisphenylethynylanthracene (**1-CBPEA**) as triplet acceptor/emitter (Fig. 11; note that for **B-3** and **B-3-Me**, the fluorescence emission shows vibrational progression, *i.e.* there are two emission bands at 616 nm and 657 nm. Actually, there should be no peak at 550 nm; this peak is due to the filter because the transmittance is greatly reduced beyond 570 nm; thus the tailing of the emission band gives a fake ‘peak’ at 550 nm). For **B-3·C-1**, upconverted emission in the 450–550 nm region was observed with increasing **C-1** concentration (Fig. 11a). For **B-3-Me**, which is unable to form a H-bonded molecular assembly, no TTA upconversion in the region of 450–550 nm was observed. Thus, we confirm that the triplet state production by **B-3·C-1** upon photoexcitation is sufficient for TTA upconversion. For the **B-3-Me**–**C-1** mixture, however, no upconversion was observed due to the lack of H-bonding. To the best of our knowledge, this is the first time that a supramolecular triplet photosensitizer was used for TTA upconversion. This result opens a new avenue for studies on TTA upconversion.

In order to prove that the production of the triplet excited state and the TTA upconversion are due to the formation of the H-bonded molecular assembly, hexafluoroisopropanol (HFIP) was added to disrupt the H-bonding (Fig. 11c). As a result, the TTA upconversion intensity decreased, and at the same time, the fluorescence of the antenna was enhanced. These changes are in agreement with the fact that the H-bonding was disrupted. Note that the upconversion is weak, but this proof-of-concept approach will be useful for designing supramolecular triplet photosensitizers and for the application of these compounds. For **B-3-Me**, no such enhancement of the fluorescence of the antenna was observed in the presence of HFIP (Fig. 11d).

## 3. Conclusion

In summary, hydrogen bond mediated supramolecular assemblies of Bodipy and C_60_ modules were prepared and acted as supramolecular triplet photosensitizers. Intra-assembly singlet and triplet state energy transfers were observed. The molecular assemblies were used as supramolecular triplet photosensitizers for triplet–triplet annihilation upconversion. Three different thymine-containing visible light-harvesting Bodipy derivatives (**B-1**, **B-2** and **B-3**, which show absorption at 505 nm, 630 nm and 593 nm, respectively) were used as the H-bonding modules, whereas 1,6-diaminopyridine appended C_60_ was used as the complementary hydrogen bonding module (**C-1**). Visible light-harvesting antennae with methylated thymine were prepared as reference compounds (**B-1-Me**, **B-2-Me** and **B-3-Me**), which are unable to form strong H-bonds with **C-1**. Triple H-bonds are formed between each Bodipy module (**B-1**, **B-2** and **B-3** visible light-harvesting antennae) and the bonding module (**C-1**). The photophysical properties of the assemblies were studied with steady state UV/vis absorption spectroscopy, fluorescence emission spectroscopy, electrochemical characterization, and nanosecond transient absorption spectroscopy. Singlet energy transfer from the Bodipy antenna to the C_60_ module was confirmed by the fluorescence quenching studies. The methylated modules were unable to form strong H-bonds with **C-1**, and fluorescence quenching was observed to a lesser extent. The nanosecond transient absorption spectroscopy showed that the triplet state is either localized on the C_60_ module (assembly **B-1·C-1**), or on the Bodipy antenna (for assemblies **B-2·C-1** and **B-3·C-1**). For assembly **B-1·C-1**, the photophysical process involves intra-assembly singlet energy transfer from the antenna to the C_60_ module, and then intersystem crossing of the latter produces the triplet excited state. The triplet state is localized on the C_60_ part in assembly **B-1·C-1**. For **B-2·C-1** and **B-3·C-1**, intra-assembly ping-pong singlet/triplet energy transfer was proposed, and as a result the triplet state is confined on the styryl-Bodipy part in **B-2·C-1** and **B-3·C-1**. No significant triplet state production was observed for the reference mixtures of **B-1-Me/C-1**, **B-2-Me/C-1** and **B-3-Me/C-1**, for which no strong H-bonds were formed. These supramolecular assemblies were used as triplet photosensitizers for triplet–triplet annihilation upconversion. These studies may offer a new approach for designing organic triplet photosensitizers.

## 4. Experimental section

### 4.1 Materials

UV/vis absorption spectra were measured with an Agilent 8453 spectrophotometer. Fluorescence spectra were recorded on a Shimadzu RF-5301 PC spectrofluorometer. Luminescence lifetimes were measured on an OB920 luminescence lifetime spectrometer (Edinburgh Instruments, UK).

### 4.2 Syntheses

#### Synthesis of **C-1**

**8** (0.043 mmol, 42.7 mg), sodium ascorbate (0.0237 mmol, 4.7 mg) and CuSO_4_ (0.0118 mmol, 2.9 mg) were added successively to a solution of **3** (0.04 mmol, 10 mg) in a mixed CHCl_3_–EtOH–water (12 : 1 : 1, v/v, 28 mL) solvent. The mixture was stirred at RT for 12 h. Then water was added, and the mixture was extracted with CH_2_Cl_2_. The organic phase was dried over Na_2_SO_4_. The solvent was evaporated under reduced pressure. The crude product was purified by column chromatography (silica gel, CH_2_Cl_2_–CH_3_OH, 50 : 1, v/v) to afford a gray solid. Yield: 26.5 mg (50.2%). ^1^H NMR (500 MHz, DMSO-d_6_): 10.59 (s, 1H), 10.13 (s, 1H), 8.24 (s, 1H), 7.76–7.65 (m, 3H), 7.49 (d, 2H, *J* = 8.3 Hz), 7.11 (d, 2H, *J* = 8.3 Hz), 5.50–5.46 (m, 4H), 5.17 (s, 2H), 4.50–4.44 (m, 2H), 2.12 (s, 3H), 1.29 (t, 3H, *J* = 7.0 Hz). ^13^C NMR (100 MHz, DMSO-d_6_): 162.5, 144.2, 144.1, 144.0, 143.8, 143.7, 143.2, 140.0, 138.4, 137.7, 131.1, 114.5, 71.1, 60.7, 52.1, 30.7, 23.9, 13.9. HRMS: *m*/*z* calcd for [C_75_H_14_O_5_]^+^: 994.0841; found: 994.0844.

#### Synthesis of **B-1**

**18** (0.18 mmol, 30 mg), sodium ascorbate (0.0237 mmol, 4.7 mg) and CuSO_4_ (0.0118 mmol, 2.9 mg) were added to a solution of **11** (0.18 mmol, 75 mg) in CHCl_3_–EtOH–water (12 : 1 : 1, v/v, 28 mL) and the mixture was stirred at RT for 12 h. In a typical work-up, water was added and the mixture was extracted with CH_2_Cl_2_. The organic phase was dried over sodium sulfate. After removal of the solvent, the product was purified by column chromatography (silica gel, CH_2_Cl_2_–CH_3_OH, 25 : 1, v/v) to afford an orange solid. Yield: 33.6 mg (32.6%). ^1^H NMR (500 MHz, DMSO-*d*_6_): 11.3 (s, 1H), 8.18 (s, 1H), 7.64 (s, 1H), 7.26 (d, 2H, *J* = 8.4 Hz), 7.09 (d, 2H, *J* = 8.5 Hz), 6.17 (s, 2H), 4.91 (s, 2H), 4.79 (t, 2H, *J* = 4.7 Hz), 4.46 (d, 2H, *J* = 4.6 Hz), 2.24 (s, 6H), 1.74 (s, 3H), 1.36 (s, 6H). ^13^C NMR (100 MHz, CDCl_3_): 163.8, 155.5, 150.6, 140.2, 128.2, 121.2, 114.9, 111.2, 66.3, 49.9, 42.9, 29.7, 14.6, 12.3. HRMS (MALDI): *m*/*z* calcd for [C_29_H_30_N_7_O_3_F_2_B]^+^: 573.2741; found: 573.2504.

#### Synthesis of **B-1-Me**

**19** (0.15 mmol, 26 mg), sodium ascorbate (0.0237 mmol, 4.7 mg) and CuSO_4_ (0.0118 mmol, 2.9 mg) were added to a solution of **11** (0.15 mmol, 65 mg) in CHCl_3_–EtOH–water (12 : 1 : 1, v/v, 14 mL) and the mixture was stirred at RT for 12 h. After the typical work-up, the crude product was purified by column chromatography (silica gel, CH_2_Cl_2_–CH_3_OH, 40 : 1, v/v) to afford an orange solid. Yield: 35.6 mg (40.2%). ^1^H NMR (500 MHz, CDCl_3_): 8.00 (s, 1H), 7.41 (s, 1H), 7.20 (d, 2H, *J* = 6.5 Hz), 6.99 (d, 2H, *J* = 8.1 Hz), 5.98 (s, 2H), 5.03 (s, 2H), 4.82 (s, 2H), 4.43 (s, 2H), 3.33 (s, 3H), 2.54 (s, 6H), 1.93 (s, 3H), 1.39 (s, 6H). ^13^C NMR (100 MHz, CDCl_3_): 164.1, 158.8, 155.6, 151.8, 140.8, 131.9, 128.5, 121.4, 115.3, 110.5, 66.2, 50.4, 44.1, 28.1, 13.2. HRMS (MALDI): *m*/*z* calcd for [C_30_H_32_N_7_O_3_F_2_B]^+^: 587.2628; found: 587.2614.

#### Synthesis of **B-2**

**18** (0.07 mmol, 11.2 mg), sodium ascorbate (0.047 mmol, 9.4 mg) and CuSO_4_ (0.0236 mmol, 5.8 mg) were added to a solution of **12** (0.07 mmol, 40 mg) in CHCl_3_–EtOH–water (12 : 1 : 1, v/v, 14 mL) and the mixture was stirred at RT for 12 h. After the work-up, the crude product was purified by column chromatography (silica gel, CH_2_Cl_2_–CH_3_OH, 25 : 1, v/v) to afford a blue solid. Yield: 33 mg (62.9%). ^1^H NMR (500 MHz, CDCl_3_): 8.64 (s, 1H), 7.94 (s, 1H), 7.75–7.84 (m, 6H), 7.41–7.26 (m, 11H), 7.00 (s, 2H), 6.64 (s, 2H), 4.96 (s, 2H), 4.80 (s, 2H), 4.41 (s, 2H), 1.88 (s, 3H), 1.46 (s, 6H). ^13^C NMR (100 MHz, CDCl_3_): 169.3, 163.5, 157.4, 156.0, 147.8, 145.4, 143.6, 141.2, 138.6, 134.9, 132.4, 124.1, 122.7, 120.3, 115.7, 71.5, 54.9, 48.0, 34.7, 19.7, 17.0. HRMS (MALDI): *m*/*z* calcd for [C_43_H_38_N_7_O_3_F_2_B]^+^: 749.3097; found: 749.3092.

#### Synthesis of **B-2-Me**

**19** (0.05 mmol, 8.5 mg), sodium ascorbate (0.047 mmol, 9.4 mg) and CuSO_4_ (0.0236 mmol, 5.8 mg) were added to a solution of **12** (0.05 mmol, 28 mg) in CHCl_3_–EtOH–water (12 : 1 : 1, v/v, 14 mL) and the mixture was stirred at RT for 12 h. After the work-up, the crude product was purified by column chromatography (silica gel, CH_2_Cl_2_–CH_3_OH, 40 : 1, v/v) to afford a blue solid. Yield: 33 mg (68.1%). ^1^H NMR (400 MHz, CDCl_3_): 7.91 (s, 1H), 7.77–7.63 (m, 6H), 7.39–7.68 (m, 13H), 7.64 (s, 2H), 6.64 (s, 2H), 5.00 (s, 2H), 4.77 (s, 2H), 4.40 (s, 2H), 3.34 (s, 3H), 1.93 (s, 3H), 1.46 (s, 6H). ^13^C NMR (100 MHz, CDCl_3_): 164.1, 158.5, 152.8, 152.0, 151.7, 148.0, 142.2, 138.7, 137.7, 136.4, 133.8, 130.1, 129.0, 127.7, 125.0, 119.4, 118.0, 115.2, 110.4, 66.4, 50.0, 44.1, 28.1, 15.1, 13.2. HRMS (MALDI): *m*/*z* calcd for [C_44_H_40_N_7_O_3_F_2_B]^+^: 763.3254; found: 763.3294.

#### Synthesis of **B-3**

**18** (0.05 mmol, 8.2 mg), sodium ascorbate (0.047 mmol, 9.4 mg) and CuSO_4_ (0.0236 mmol, 5.8 mg) were added to a solution of **16** (0.055 mmol, 35 mg) in CHCl_3_–EtOH–water (12 : 1 : 1, v/v, 14 mL) and the mixture was stirred at RT for 24 h. After the work-up, the crude product was purified by column chromatography (silica gel, CH_2_Cl_2_–CH_3_OH, 25 : 1, v/v) to afford a blue solid. Yield: 22.3 mg (50.2%). ^1^H NMR (400 MHz, CDCl_3_): 8.99 (s, 1H), 8.28 (s, 1H), 8.17 (d, 2H, *J* = 7.6 Hz), 7.79–7.69 (m, 2H), 7.50–7.38 (m, 6H), 7.24–7.22 (m, 3H), 7.00 (s, 2H), 6.66 (s, 1H), 5.99 (s, 1H), 5.00 (s, 2H), 4.81 (s, 2H), 4.41 (s, 3H), 4.31 (t, 2H, *J* = 6.7 Hz), 2.62 (s, 3H), 1.88 (s, 3H), 1.44–1.40 (m, 8H), 0.96 (t, 3H, *J* = 6.7 Hz). ^13^C NMR (100 MHz, CDCl_3_): 164.4, 154.1, 151.2, 141.6, 140.8, 138.2, 133.3, 130.0, 123.4, 120.8, 119.5, 117.6, 116.2, 115.1, 111.3, 109.2, 66.1, 43.1, 31.1, 30.1, 20.6, 13.9, 12.3. HRMS (MALDI): *m*/*z* calcd for [C_46_H_45_N_8_O_3_F_2_B]^+^: 806.3676; found: 806.3685.

#### Synthesis of **B-3-Me**

**19** (0.04 mmol, 7.1 mg), sodium ascorbate (0.047 mmol, 9.4 mg), CuSO_4_ (0.0236 mmol, 5.8 mg) were added to a solution of **16** (0.04 mmol, 25.6 mg) in a mixture of CHCl_3_–EtOH–water (14 mL, 12 : 1 : 1, v/v) and the mixture was stirred at RT for 24 h. After the typical work-up, the crude product was purified by column chromatography (silica gel, CH_2_Cl_2_–CH_3_OH, 40 : 1, v/v) to afford a blue solid. Yield: 19.6 mg (59.7%). ^1^H NMR (400 MHz, CDCl_3_): 8.28 (s, 1H), 8.17 (d, 2H, *J* = 9.5 Hz), 7.78–7.69 (m, 2H), 7.50–7.38 (m, 5H), 7.25–7.22 (m, 4H), 7.00 (d, 2H, *J* = 7.4 Hz), 6.67 (s, 1H), 5.99 (s, 1H), 5.02 (s, 2H), 4.80 (s, 2H), 4.42 (s, 2H), 4.31 (t, 2H, *J* = 7.5 Hz), 3.34 (s, 3H), 2.62 (s, 3H), 1.93 (s, 3H), 1.47–1.38 (m, 8H), 0.96 (t, 3H, *J* = 6.4 Hz). ^13^C NMR (100 MHz, CDCl_3_): 164.1, 158.1, 154.3, 152.0, 143.0, 141.1, 138.3, 133.4, 128.8, 125.8, 123.5, 119.5, 117.6, 114.5, 110.5, 109.5, 66.2, 43.2, 29.5, 28.1, 20.92, 14.1, 12.9. HRMS (MALDI): *m*/*z* calcd for [C_47_H_47_N_8_O_3_F_2_B]^+^: 820.3832; found: 820.3850.

### 4.3 Cyclic voltammetry

Cyclic voltammetry was performed using a CHI610D electrochemical workstation (Shanghai, China). Cyclic voltammograms were recorded at scan rates of 0.05 V s^–1^. The electrolytic cell used was a three electrode cell. Electrochemical measurements were performed at RT using 0.1 M tetrabutylammonium hexafluorophosphate (TBAP) as supporting electrolyte, after purging with N_2_. The working electrode was a glassy carbon electrode, and the counter electrode was a platinum electrode. A non-aqueous Ag/AgNO_3_ (0.1 M in acetonitrile) reference electrode was contained in a separate compartment connected to the solution *via* a semipermeable membrane. DCM was used as the solvent. Ferrocene was added as the internal reference.

### 4.4 Nanosecond transient absorption spectra

The spectra were measured on an LP920 laser flash photolysis spectrometer (Edinburgh Instruments, UK). The lifetimes (obtained by monitoring the decay traces) were calculated with the LP900 software. All the solutions were purged with N_2_ for 15 min before measurement and the gas flow was maintained during the measurements. The samples were excited with a nanosecond pulsed laser (Opolette™ 355II + UV nanosecond pulsed laser, typical pulse length: 7 ns. Pulse repetition: 20 Hz. Peak OPO energy: 4 mJ. The wavelength is tunable in the range of 410–2200 nm. OPOTEK, USA) and the transient signals were recorded on a Tektronix TDS 3012B oscilloscope.

The quantum yield of the triplet formation (*Φ*_T_) was determined using the sensitizing method, which was described in detail previously.16b,c,^54^ The method is based on the use of a triplet energy acceptor (β-carotene) and a standard compound (Ru(bpy)_3_Cl_2_, bpy = 2,2′-bipyridine, *Φ*_T_ = 1). Using optically matched solutions of the reference and the **C-1·B-1** hydrogen bonding assembly upon excitation at the same wavelength, the ΔO.D. value, the triplet–triplet energy transfer to β-carotene (*K*_TTET_, monitored for the T_1_ → T_*n*_ absorption at 515 nm), and the decay of the triplet energy donor in the absence of β-carotene (*k*_0_) were used for calculation of the *Φ*_T_ values.

### 4.5 Computational simulations of the **B-1** and **C-1** complex

#### Force field parameters of **B-1** and **C-1** structures

The structures of the **B-1** and **C-1** motifs without fullerene were first energy minimized using density functional theory at the B3LYP/6-31G* level. Frequency analyses were performed on the optimized structures in order to obtain the Hessian matrix, from which the corresponding force constants for all the bonds were calculated, as well as the equilibrium bond lengths. The electrostatic potential was calculated based on the same theory, using Mulliken population analysis. The restrained electrostatic potential (RESP) method was used to fit the electrostatic potential in order to obtain the partial atomic charges.^55^ All quantum chemical calculations were carried out with the Gaussian 09 software package.^56^ The PARATOOL plug-in of VMD was used to derive the corresponding force constants.^57^ For the fullerene motif of **C-1**, the partial charges of all carbon atoms were set to zero, as reported for previous molecular dynamics simulations of fullerene derivatives.^58^,^59^ Force constants obtained from the CHARMM force field parameters^60^ were applied for the bonding and non-bonding interactions within the fullerene motif of **C-1**.

#### Molecular dynamics simulations

The starting complex of **B-1** and **C-1** is extended, with three pairs of hydrogen bonds between **B-1** and **C-1**. This initial structure was solvated in a TIP3P water box (85 Å × 55 Å × 48 Å), resulting in a system containing 6970 water molecules. The whole system was minimized first, and the **B-1·C-1** complex was fixed for 5000 steps to remove unfavorable van der Waals contacts between solvent and solute molecules. Then the system was gradually heated to 300 K, while the **B-1·C-1** complex was constrained with harmonic restraints. After a 50 ps equilibration at the desired temperature, the whole system was simulated for another 30 ns without restraints under the *NPT* ensemble (constant number of atoms, pressure and temperature). The temperature was maintained at 310 K using Langevin dynamics, and the pressure was kept at 1 atm using the Langevin piston method. An integration time step of 2 fs was used. The long-range electrostatic interactions were treated using the PME method. The trajectory was saved every 500 steps (1 ps). A hydrogen bond is formed if the donor atoms (H atoms) are within 3 Å of the acceptor atoms (N or O atoms). All molecular dynamics simulations were performed using the NAMD 2.9 software.^61^ The non-bonded interaction parameters were taken from the CHARMM 27 force field parameters.^60^

## Supplementary Material

Supplementary information
