# Supplementary material for: Bodipy–C60 triple hydrogen bonding assemblies as heavy atom-free triplet photosensitizers: preparation and study of the singlet/triplet energy transfer
Source: Chem Sci. 2015 Apr 9;6(7):3724–37. doi: 10.1039/c4sc03865g (PMC5707450; doi:10.1039/c4sc03865g)
Supplement: Supplementary file 1 [file SC-006-C4SC03865G-s001.pdf]

Electronic Supplementary Material (ESI) for Chemical Science.
This journal is © The Royal Society of Chemistry 2015

Electronic Supplementary Information for:

Bodipy-C_{60} Triple Hydrogen Bonding Assembly as Heavy atom-free Triplet Photosensitizers: Preparation and Study of the Singlet/triplet Energy Transfer

Song Guo,[a] Liang Xu,[b] Kejing Xu,[b] Jianzhang Zhao,*[a] Betül Küçüköz,[c] Ahmet Karatay,[c] Halime Gul Yaglioglu,[c] Mustafa Hayvali,[d] Ayhan Elmali [c]

[a] State Key Laboratory of Fine Chemicals, School of Chemical Engineering, Dalian University of Technology, E-208 Western Campus, 2 Ling Gong Road, Dalian 116024, P. R. China.
E-mail: zhaojzh@dlut.edu.cn Web: http://finechem2.dlut.edu.cn/photochem;

[b] School of Chemistry, Dalian University of Technology, Dalian 116024, P. R. China;

[c] Department of Engineering Physics, Faculty of Engineering, Ankara University, 06100 Beşevler, Ankara, Turkey;

[d] Department of Chemistry, Faculty of Science, Ankara University, 06100 Beşevler, Ankara, Turkey.

Table of Contents

1.0 NMR and HRMS spectra……S2-23

2.0 Synthetic details……S24-29

3.0 Abosption and Emssion details……S29-33

4.0 Transient absorption details……S34-39

5.0 The detailed data of electrochemistry……S40-44

6.0 The detailed data of fluorenscence lifetime……S44-45

7.0 Molecular dynamics (MD) simulations……S45-46

8.0 The detailed data of Triplet-Triplet annihilation upconversion……S46-47

1.0 NMR and HR-MS spectra

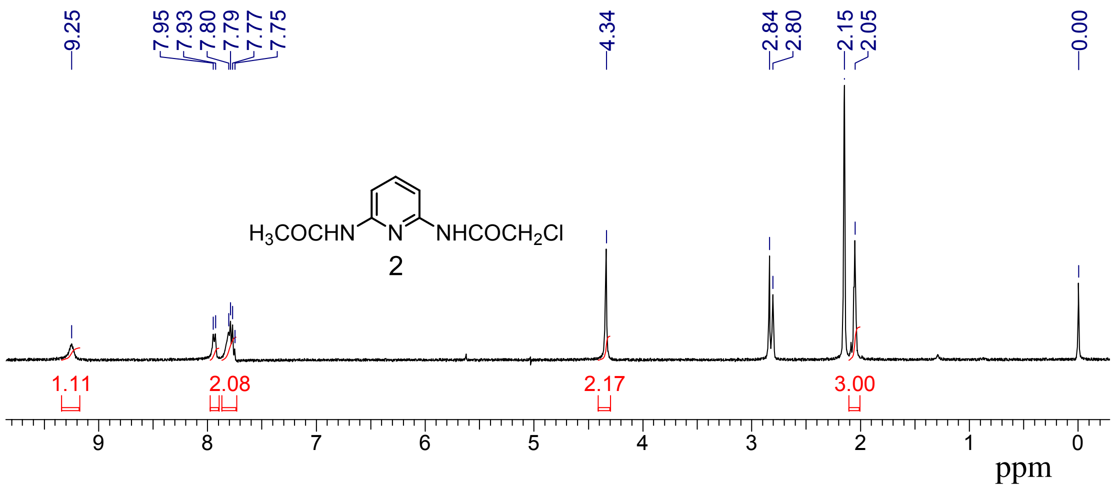

Fig. S1 ^{1}H NMR of **2** (400 MHz, Acetone-d_6).

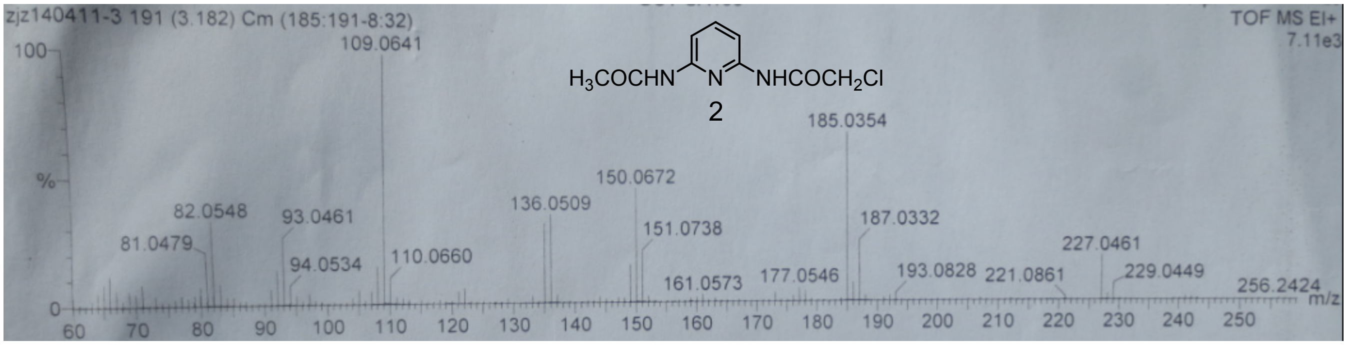

Fig. S2 TOF MS EI+ of **2.**

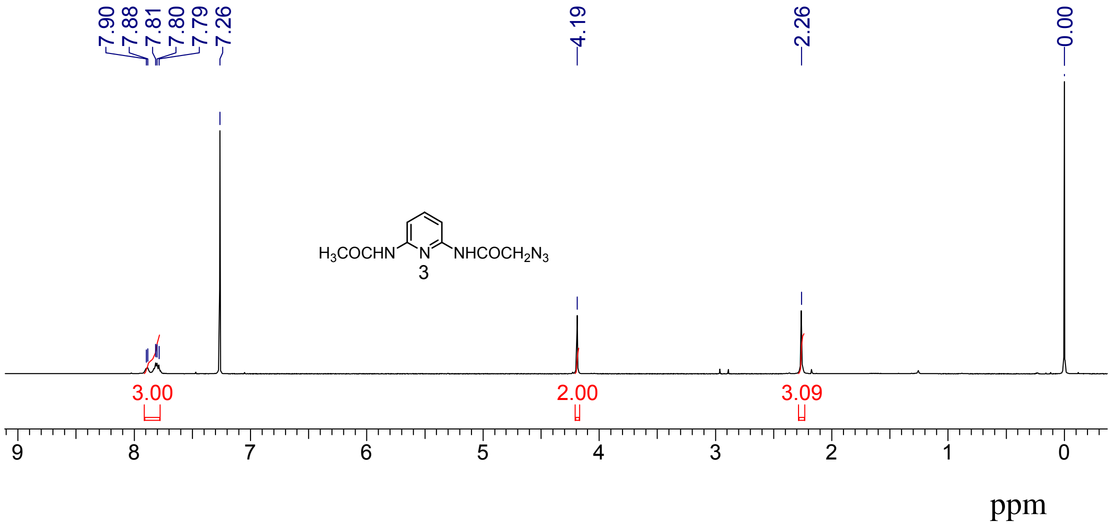

Fig. S3 ^{1}H NMR of **3** (500 MHz, $CDCl_3$).

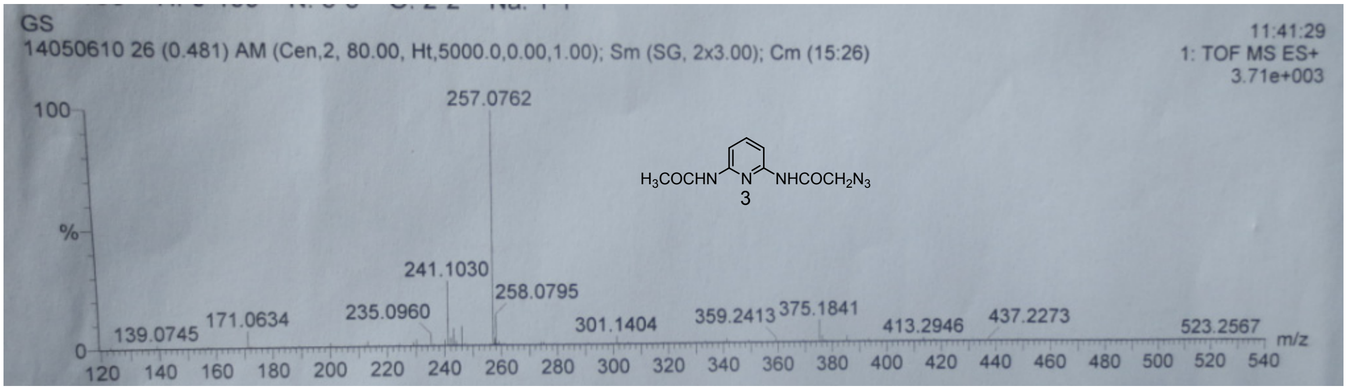

Fig. S4 TOF MS EI^+ of **3.**

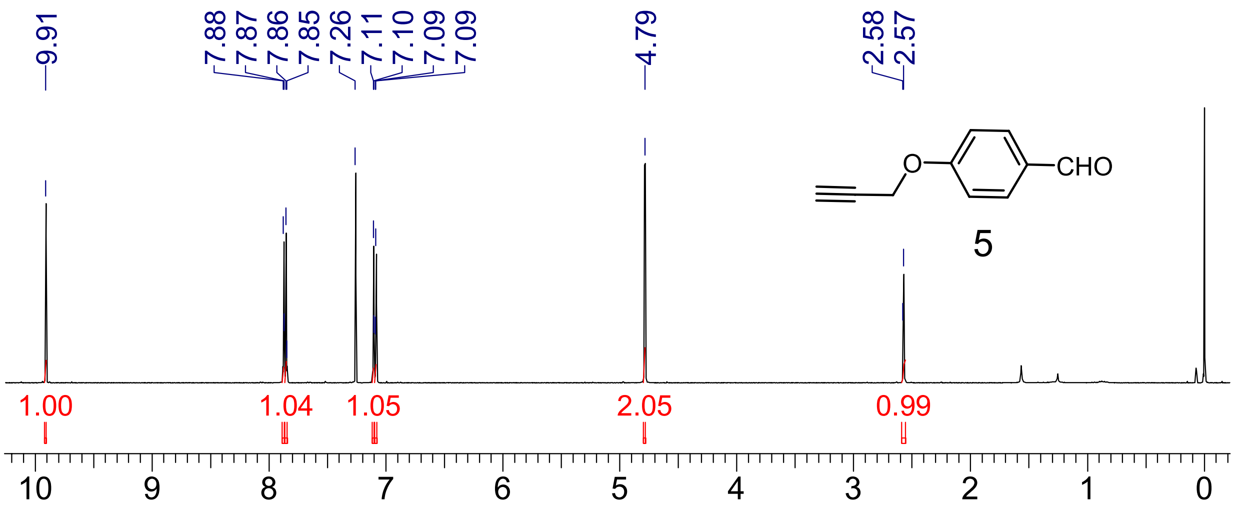

Fig. S5 ^{1}H NMR of **5** (500 MHz, $CDCl_3$).

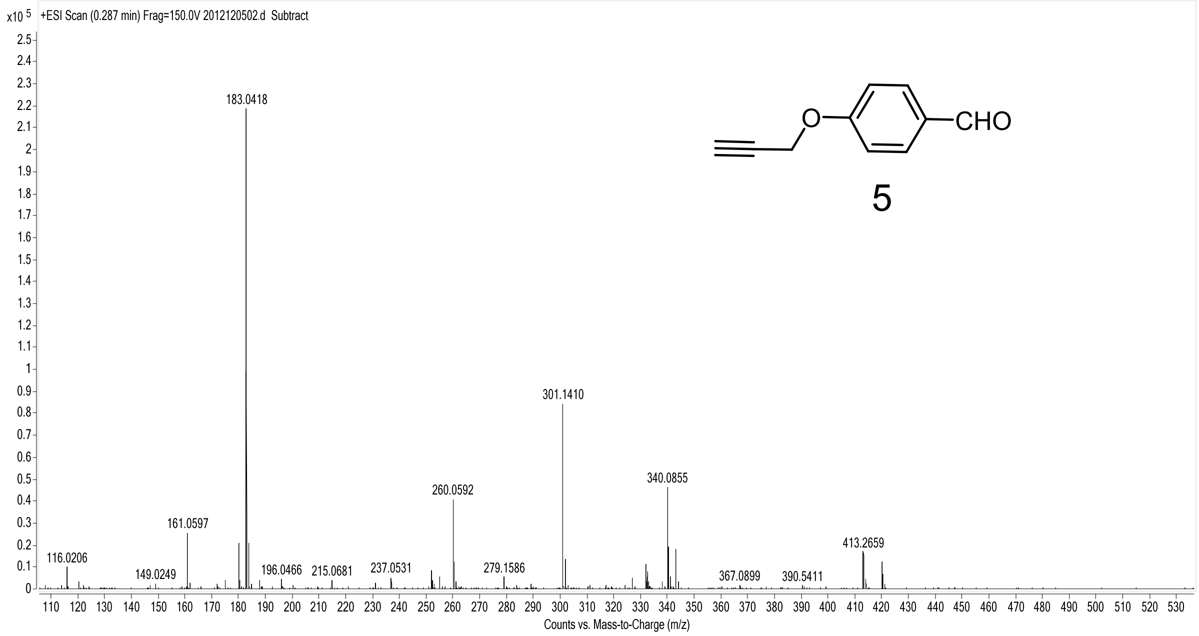

Fig. S6 TOF MS EI^{+} of **5**.

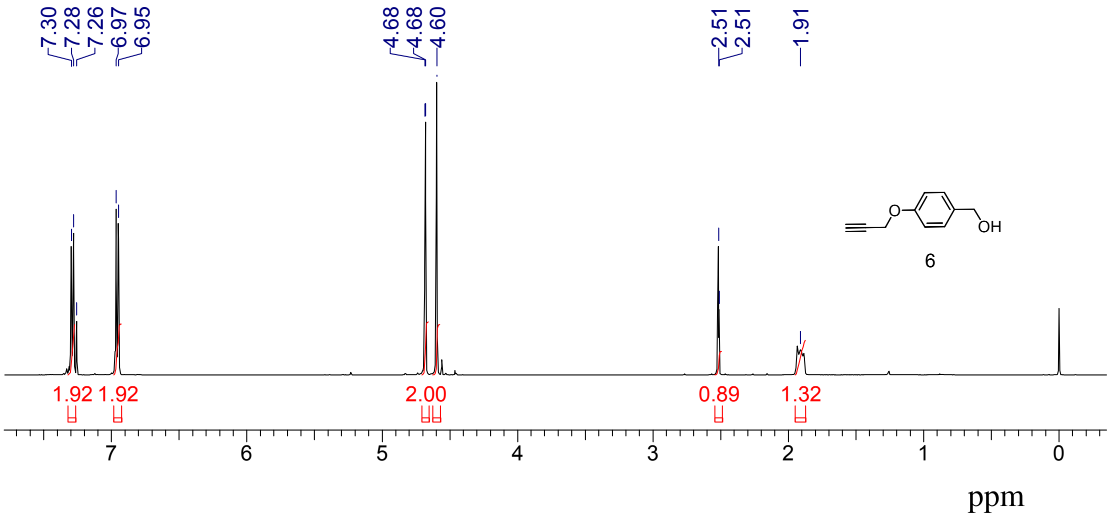

Fig. S7 ^{1}H NMR of **6** (500 MHz, $CDCl_3$).

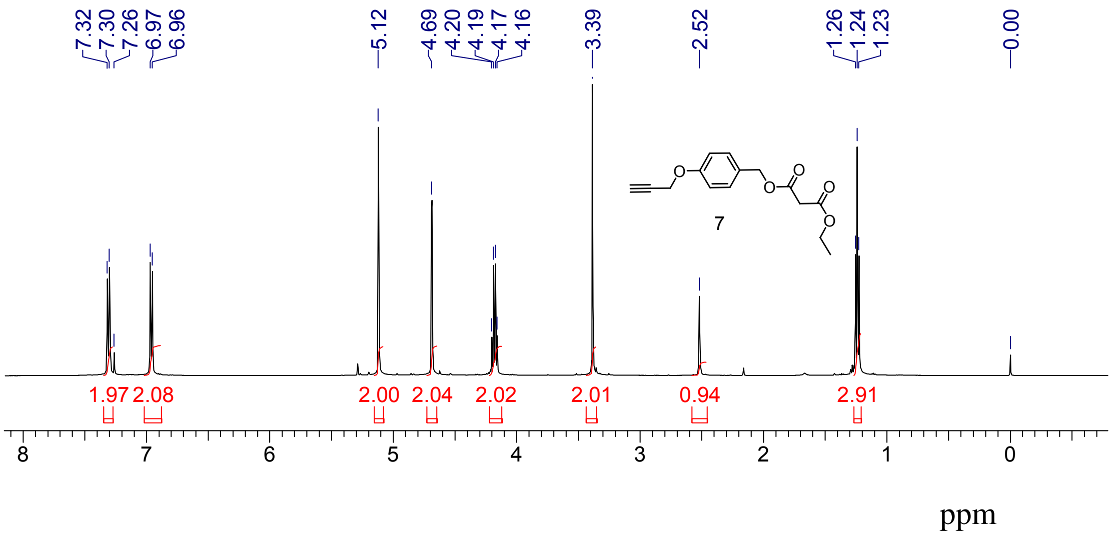

Fig. S8 ^{1}H NMR of **7** (400 MHz, $CDCl_3$).

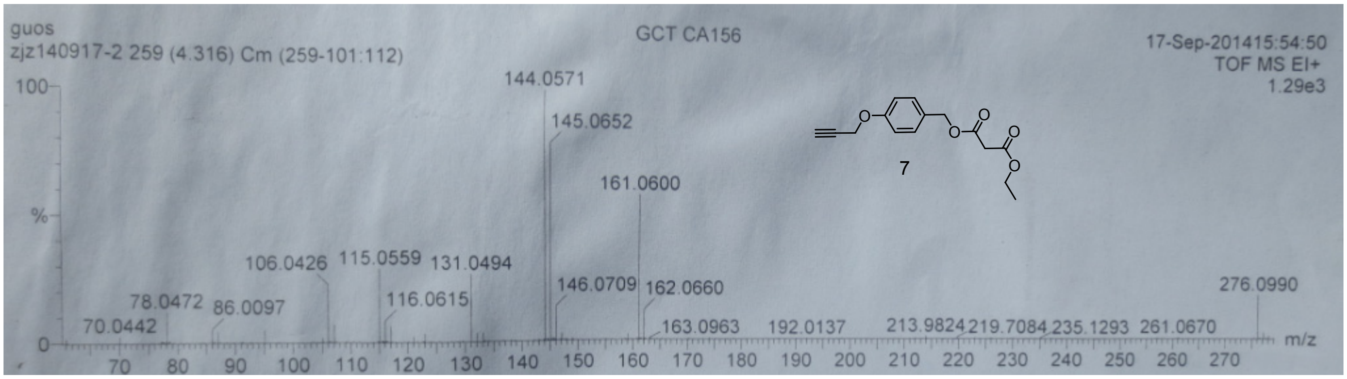

Fig. S9 TOF MS EI$^+$ of **7**.

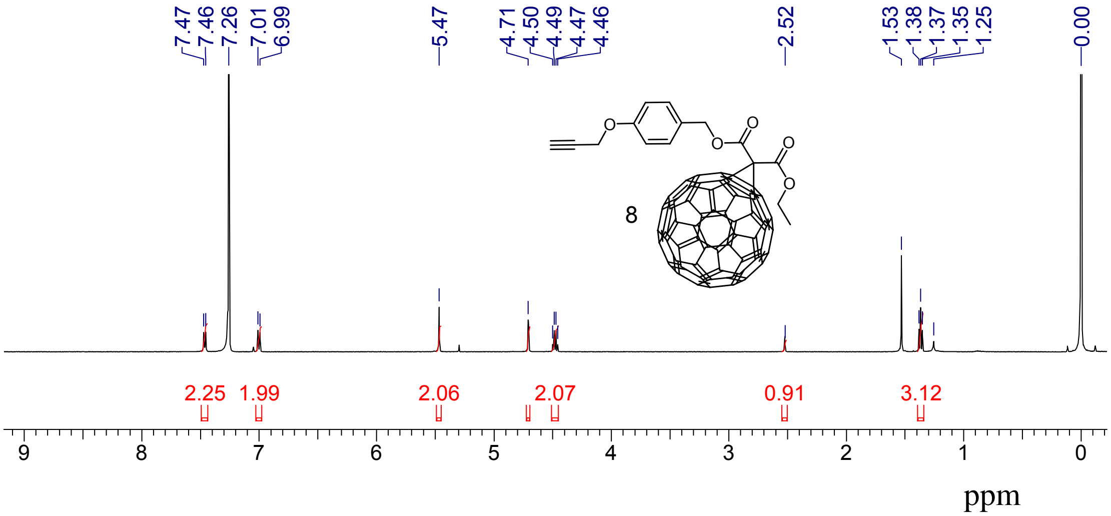

Fig. S10 ^{1}H NMR of **8** (400 MHz, $CDCl_3$).

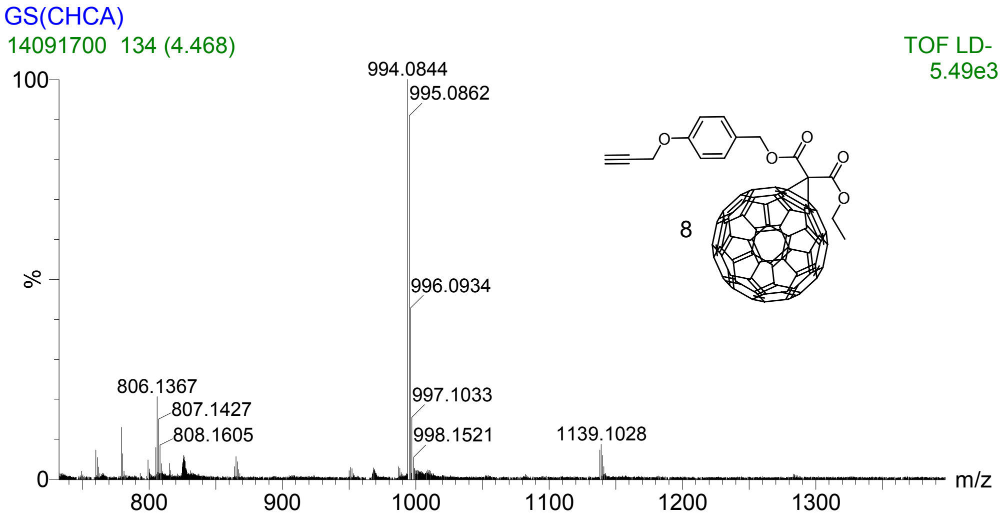

Fig. S11 TOF HRMS (MALDI) ESI of **8**.

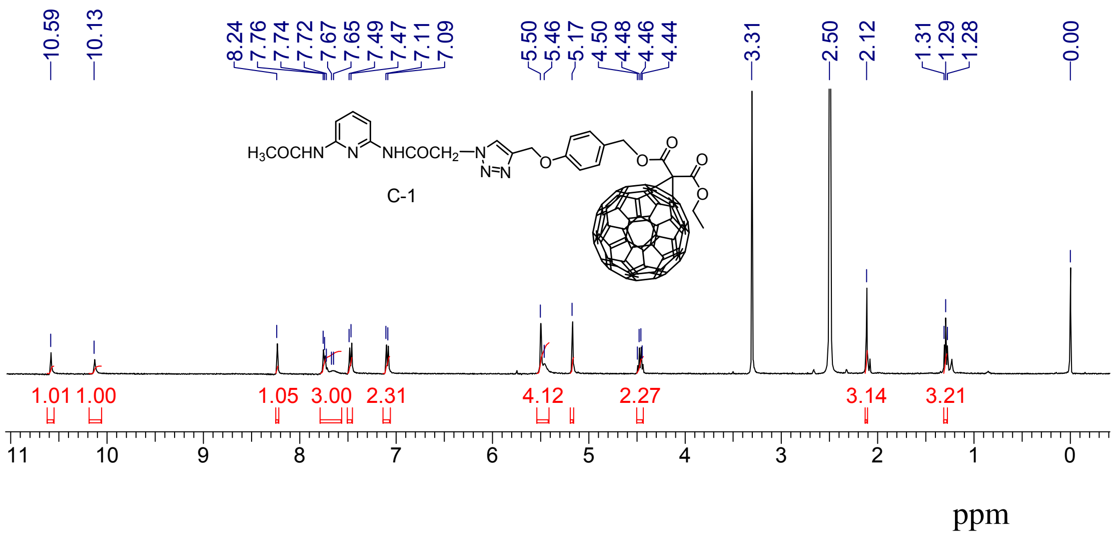

Fig. S12 ^{1}H NMR of **C-1** (500 MHz, DMSO-d_6).

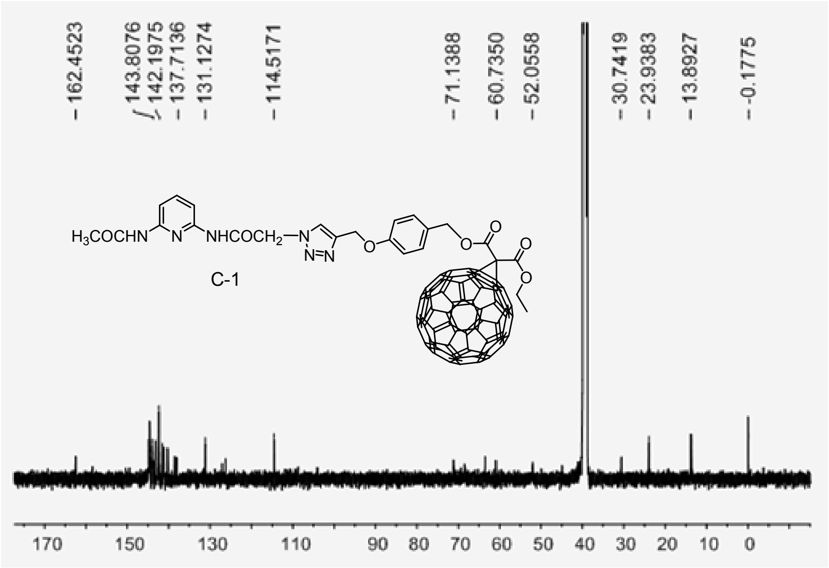

Fig. S13 [1]C NMR of **C-1** (100 MHz, DMSO-d$_6$).

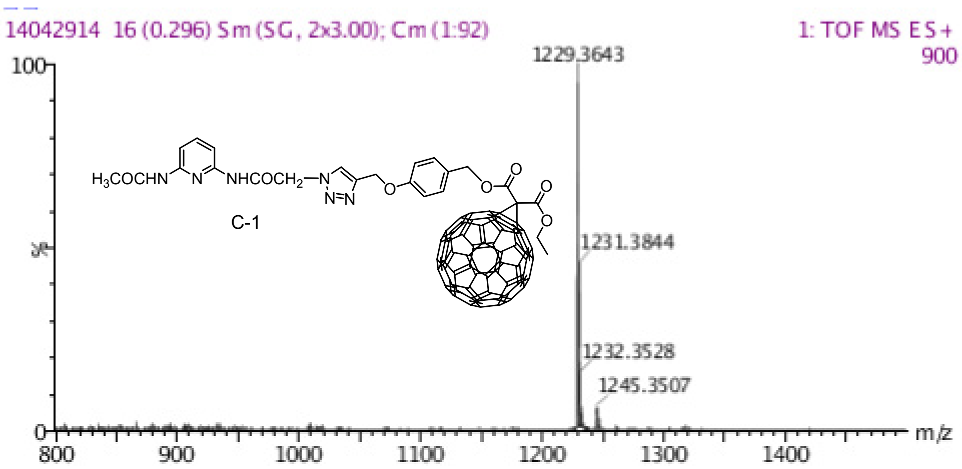

Fig. S14 TOF HRMS ESI of **C-1**.

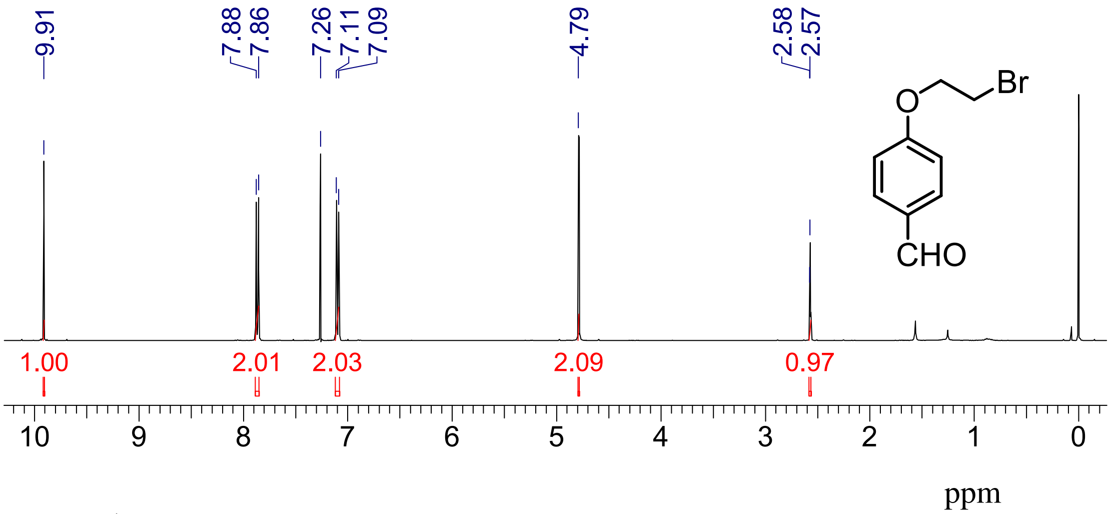

Fig. S15 ^{1}H NMR of **9** (400 MHz, $CDCl_3$).

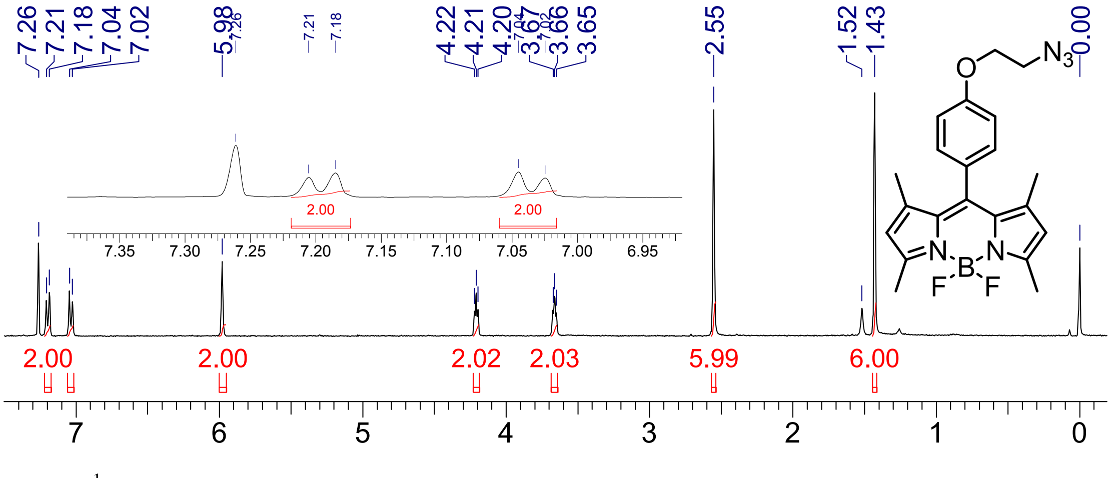

Fig. S16 ^{1}H NMR of **11** (400 MHz, $CDCl_3$).

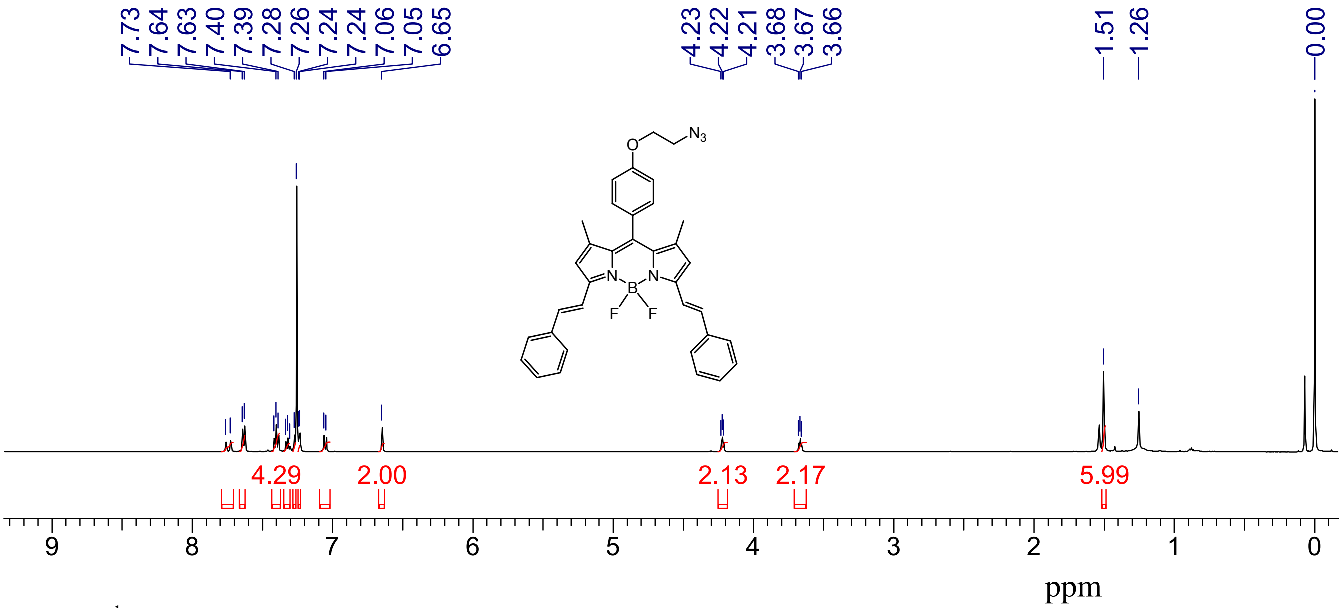

Fig. S17 ^{1}H NMR of **12** (500 MHz, $CDCl_3$).

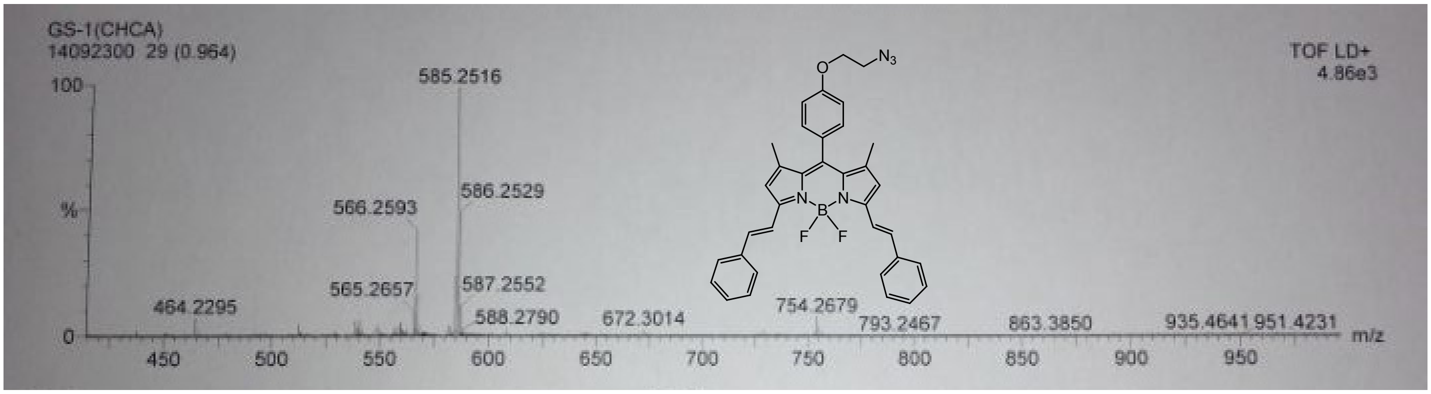

Fig. S18 TOF HRMS (MALDI) ESI of **12**.

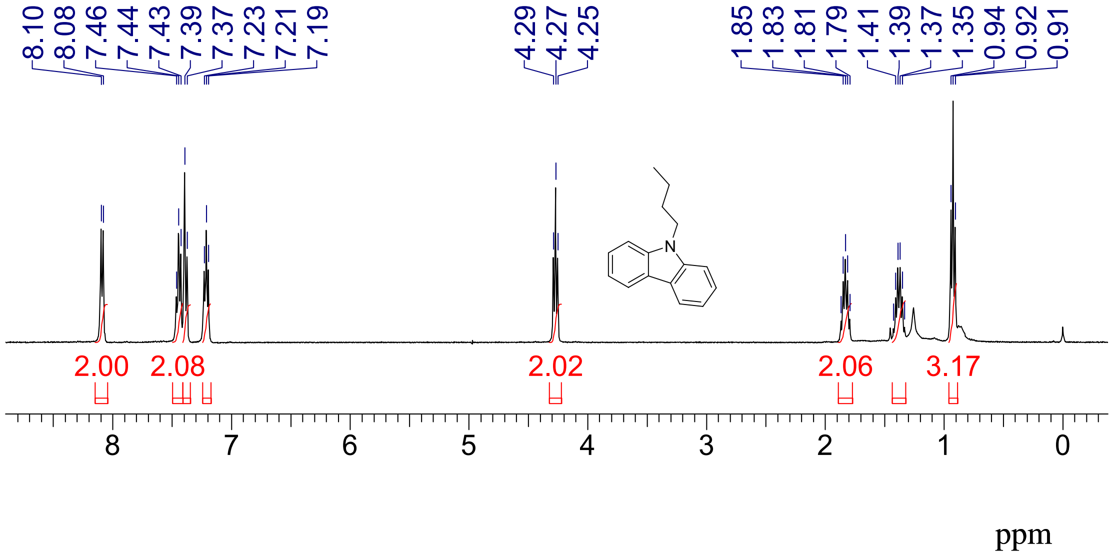

Fig. S19 ¹H NMR of **14** (400 MHz, $CDCl_3$).

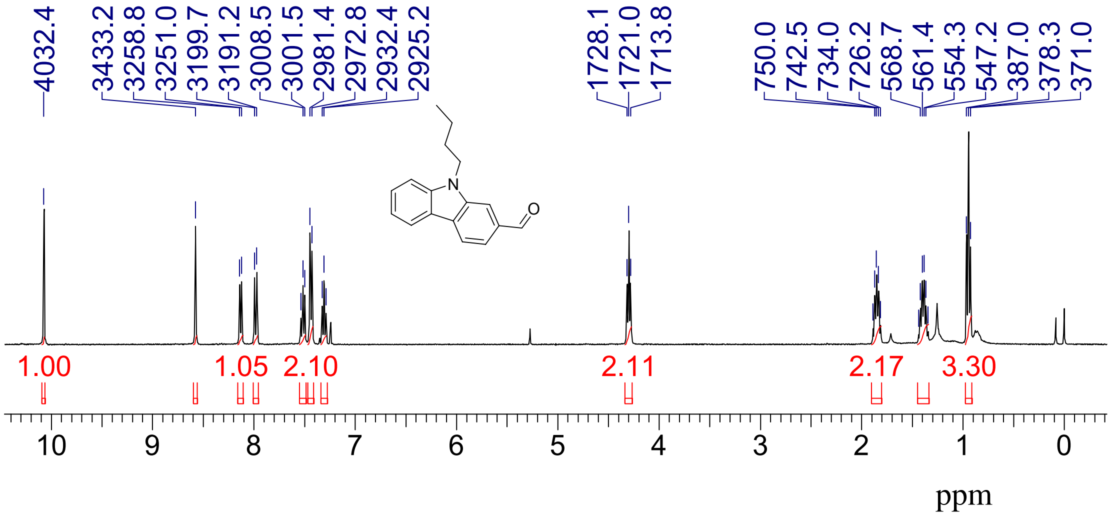

Fig. S20 ¹H NMR of **15** (400 MHz, $CDCl_3$).

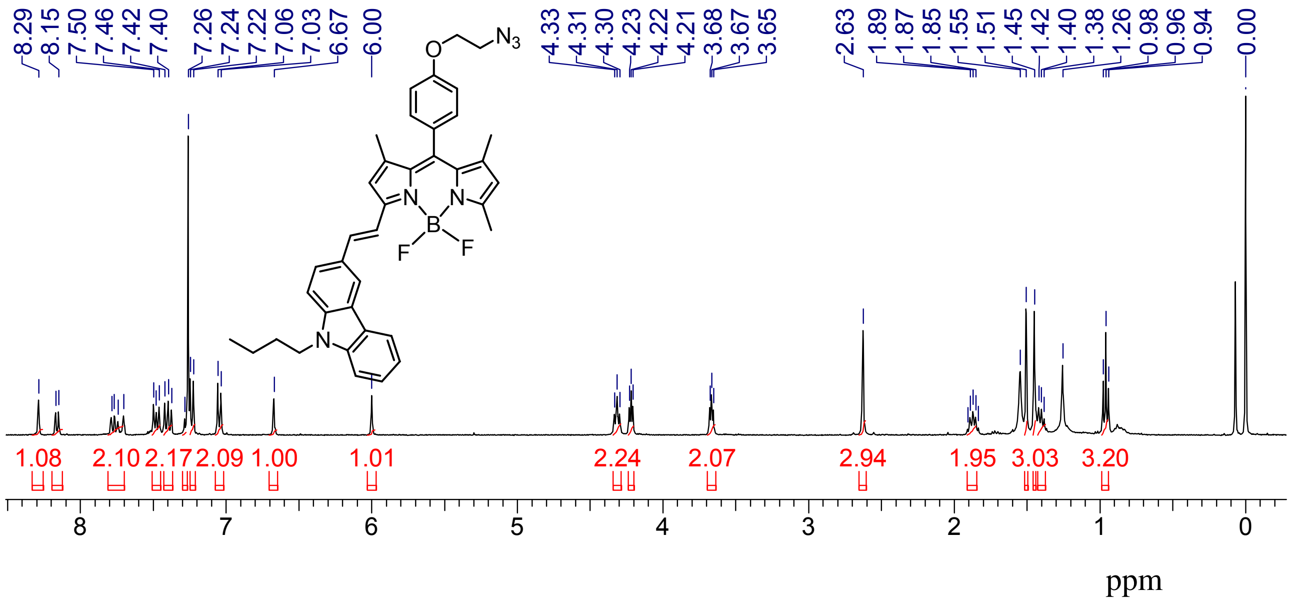

Fig. S21 ^{1}H NMR of **16** (400 MHz, $CDCl_3$).

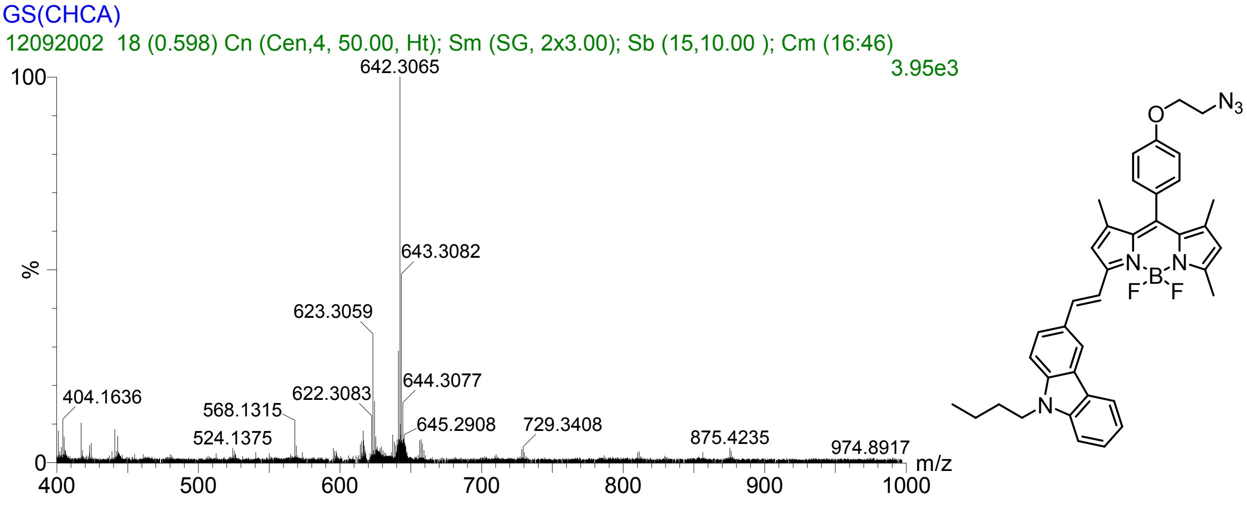

Fig. S22 TOF HRMS (MALDI) ESI of **16**.

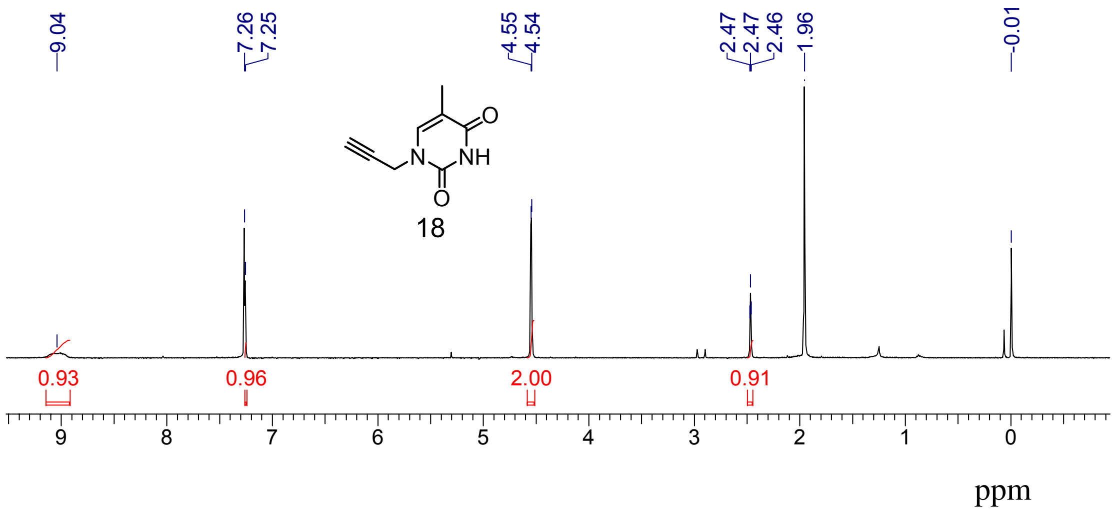

Fig. S23 ^{1}H NMR of **18** (400 MHz, $CDCl_3$).

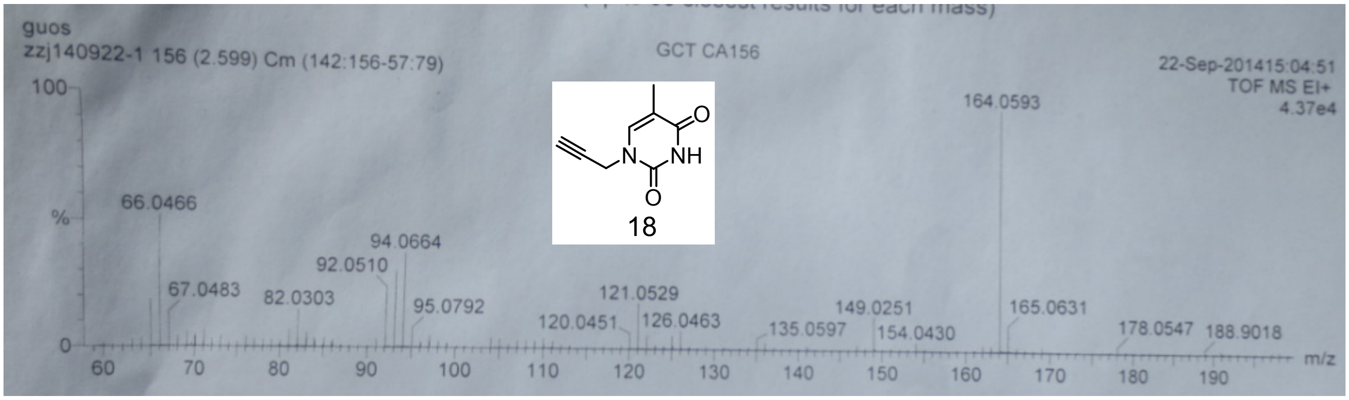

Fig. S24. TOF MS EI$^+$ of **18**.

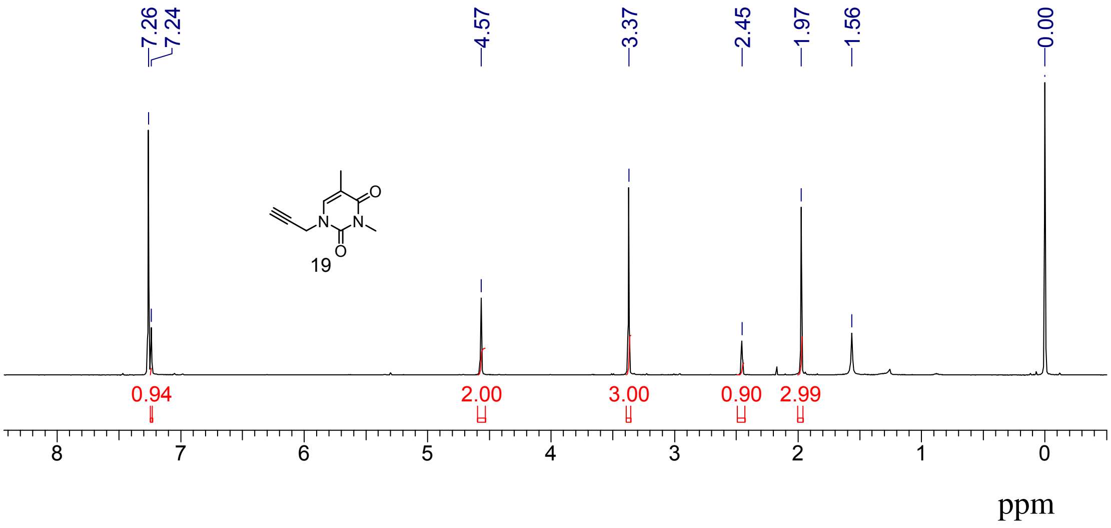

Fig. S25. ^{1}H NMR of **19** (500 MHz, $CDCl_3$).

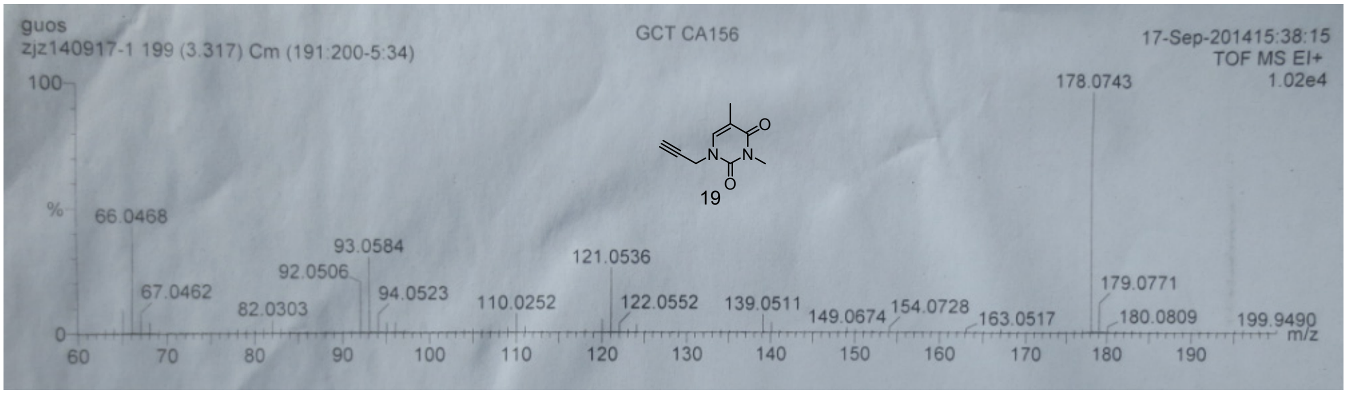

Fig. S26. TOF MS EI+ of **19**.

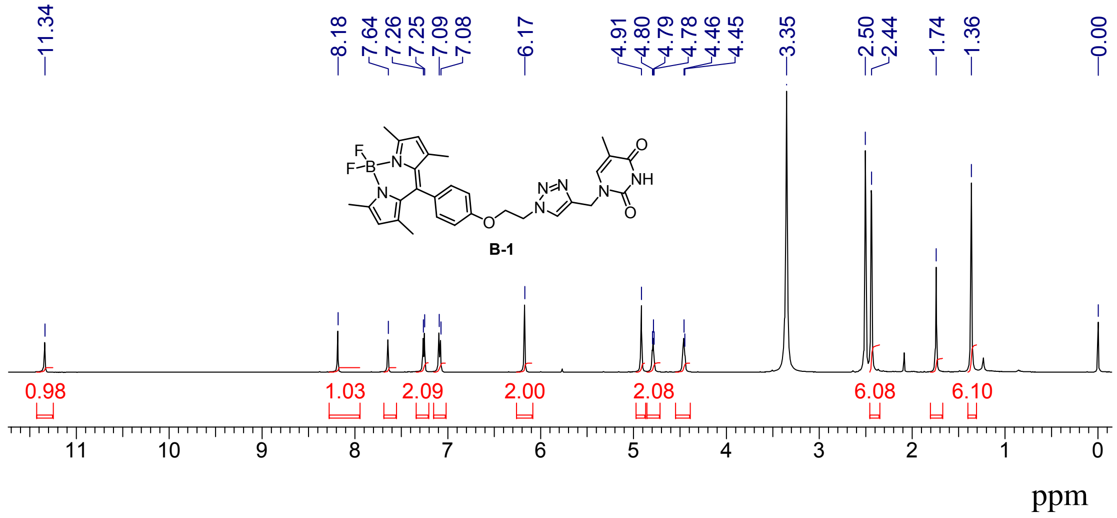

Fig. S27 ^{1}H NMR of **B-1** (500 MHz, DMSO-d$_6$).

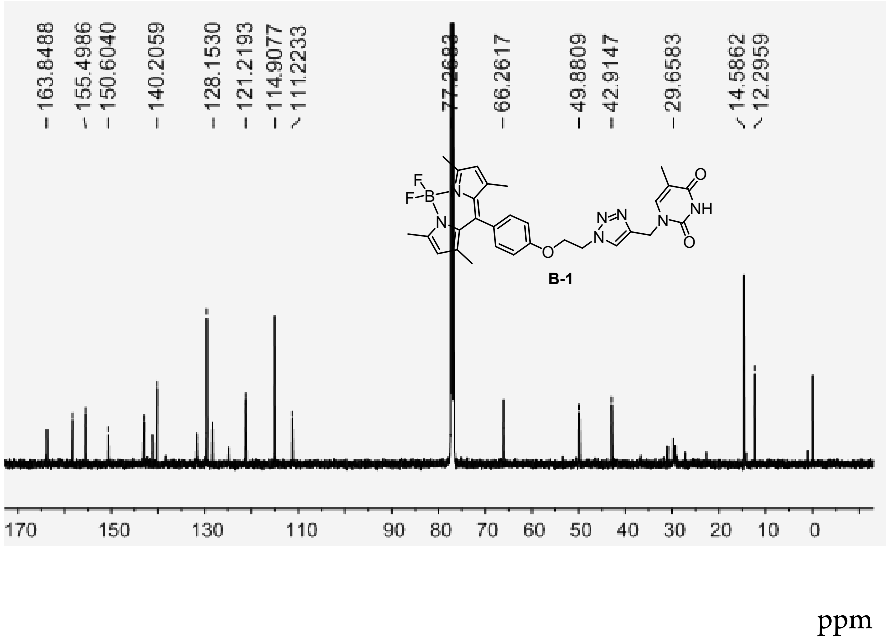

Fig. S28 ^{13}C NMR of **B-1** (100 MHz, $CDCl_3$).

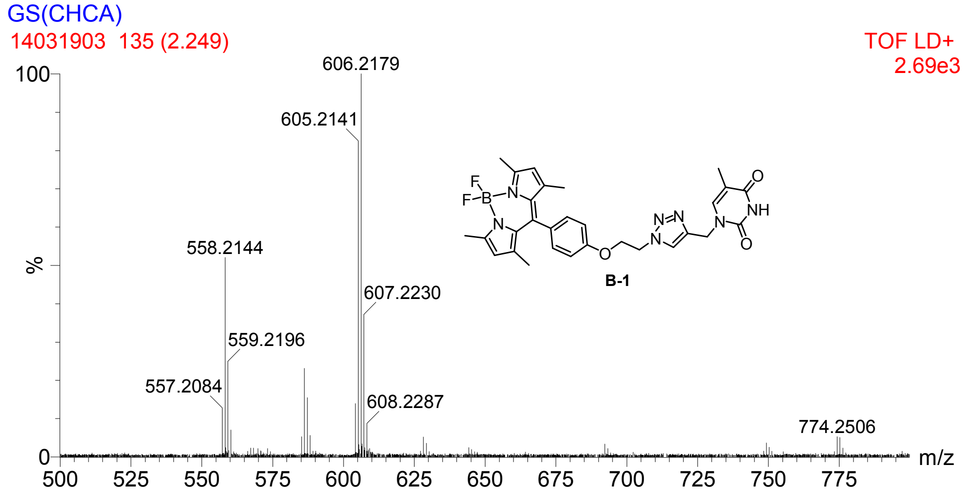

Fig. S29 TOF HRMS MALDI of **B-1**.

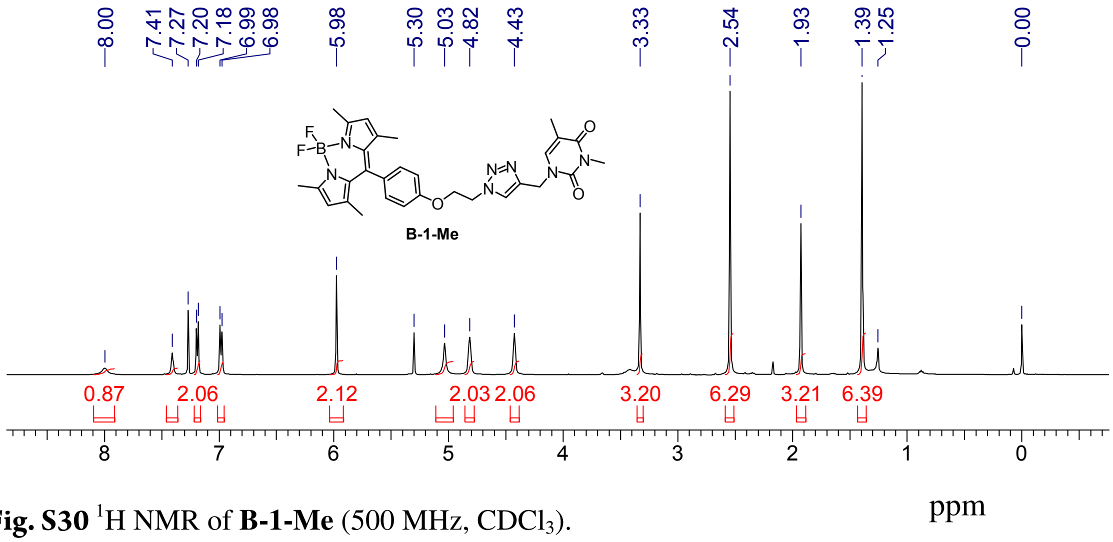

Fig. S30 ^{1}H NMR of **B-1-Me** (500 MHz, $CDCl_3$).

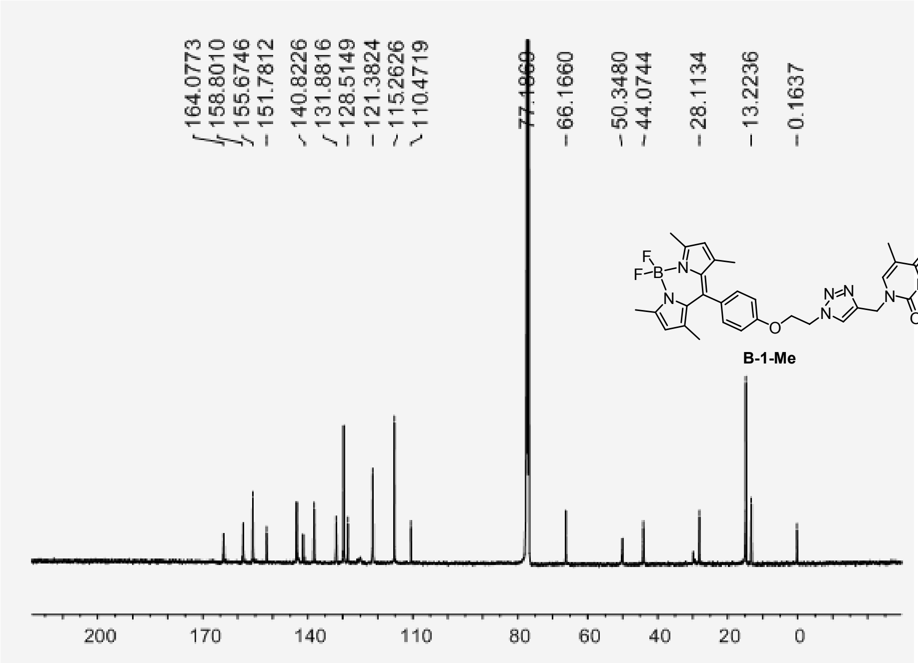

ppm

Fig. S31 ^{13}C NMR of **B-1-Me** (100 MHz, $CDCl_3$).

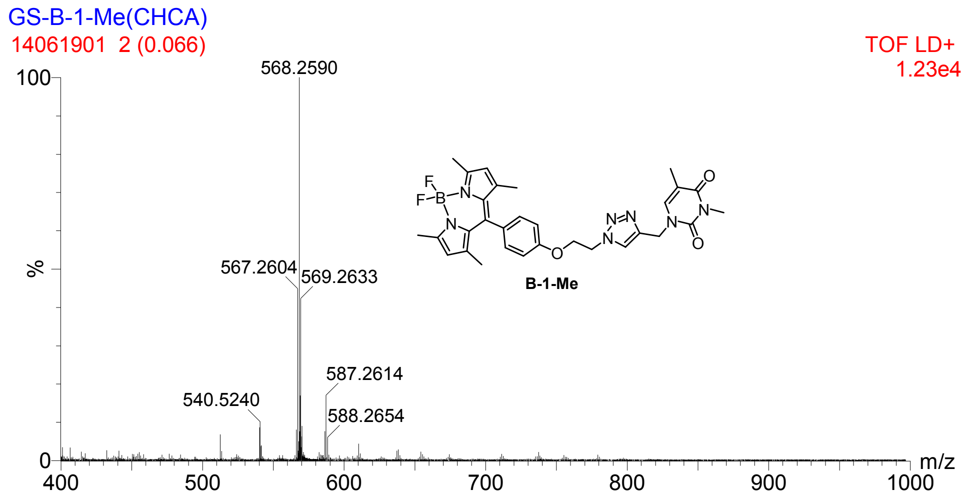

Fig. S32 TOF HRMS MALDI of **B-1-Me**.

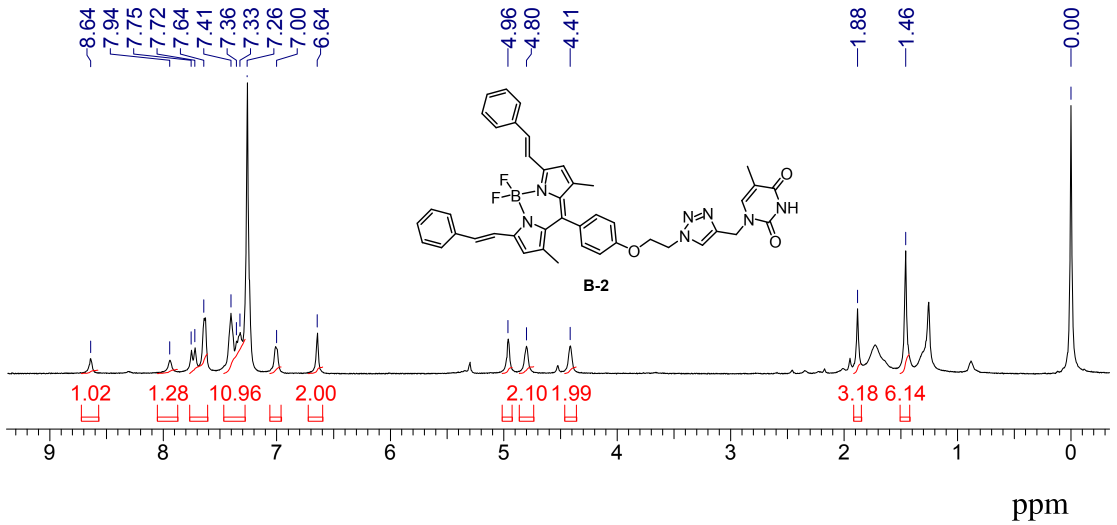

Fig. S33 ^{1}H NMR of **B-2** (500 MHz, $CDCl_3$).

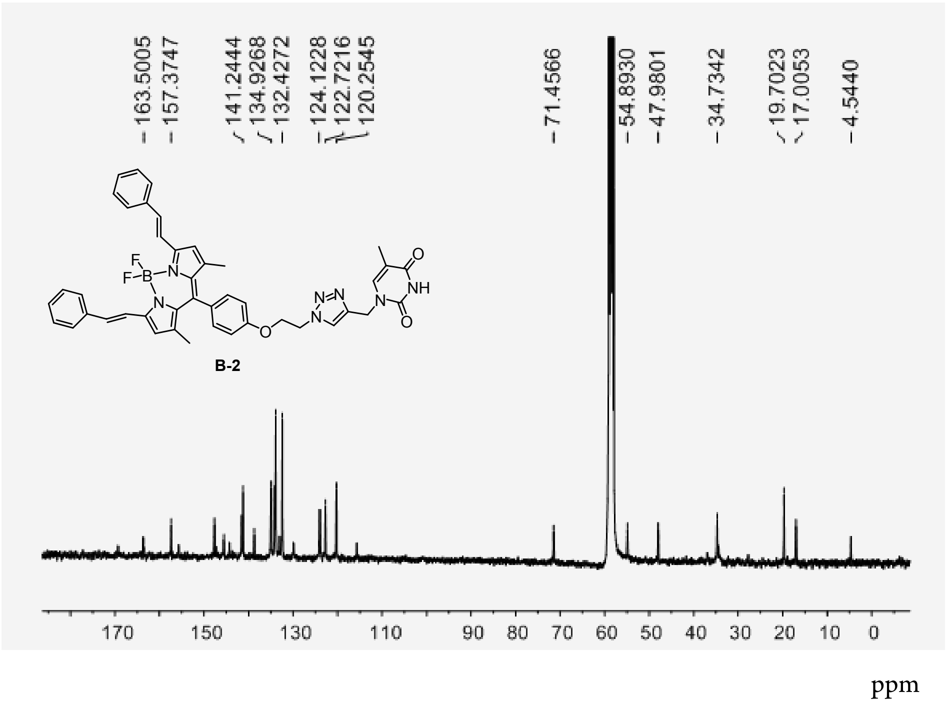

Fig. S34 ^{13}C NMR of **B-2** (100 MHz, $CDCl_3$).

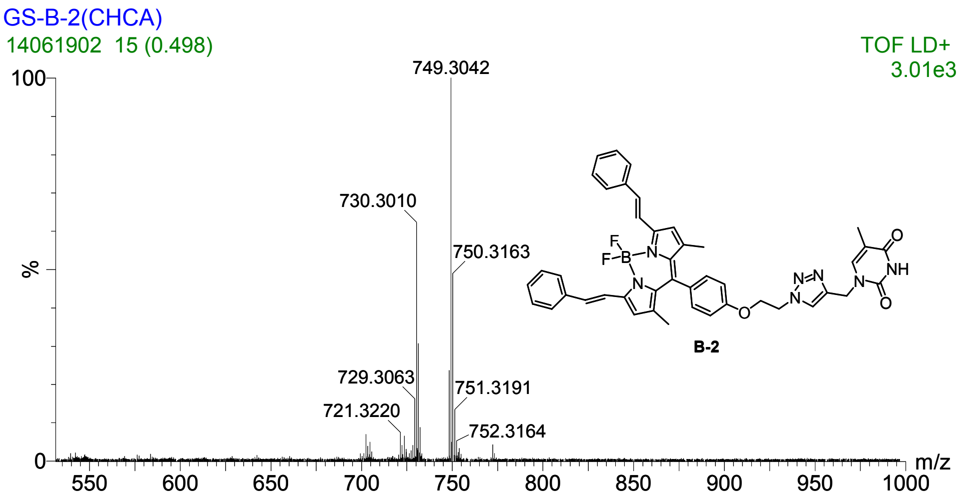

Fig. S35 TOF HRMS MALDI of **B-2**.

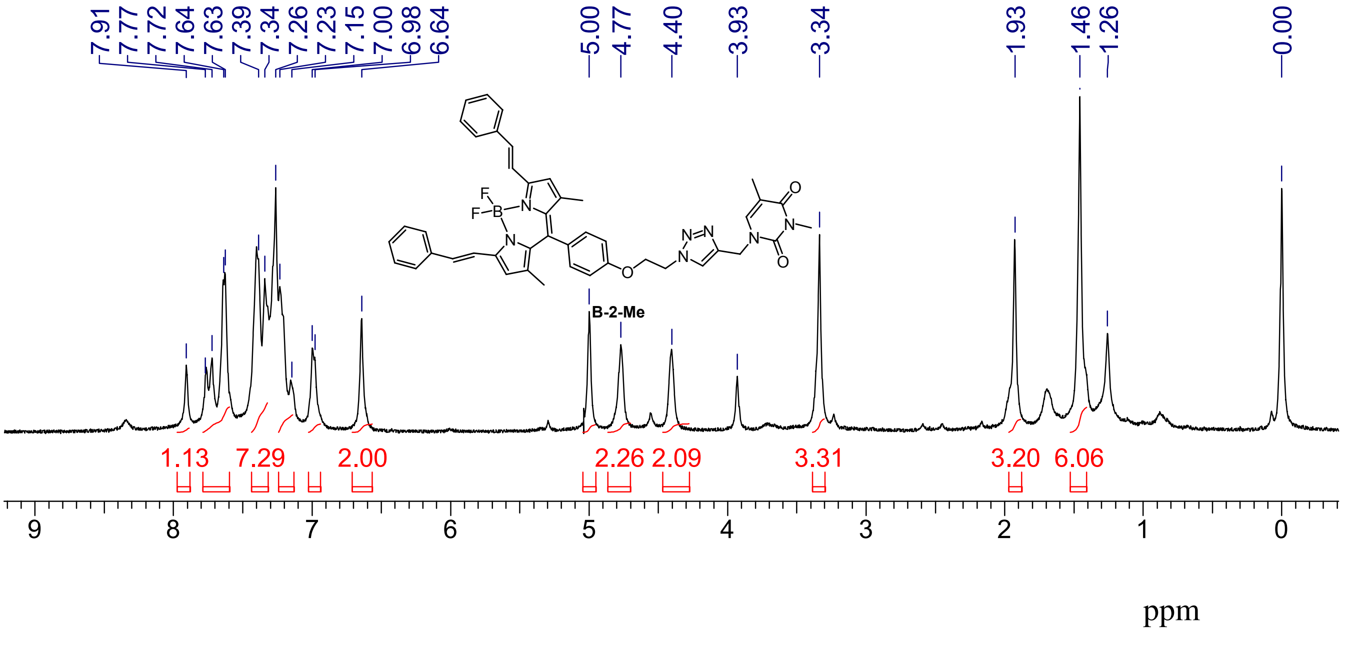

Fig. S36 ^{1}H NMR of **B-2-Me** (400 MHz, $CDCl_3$).

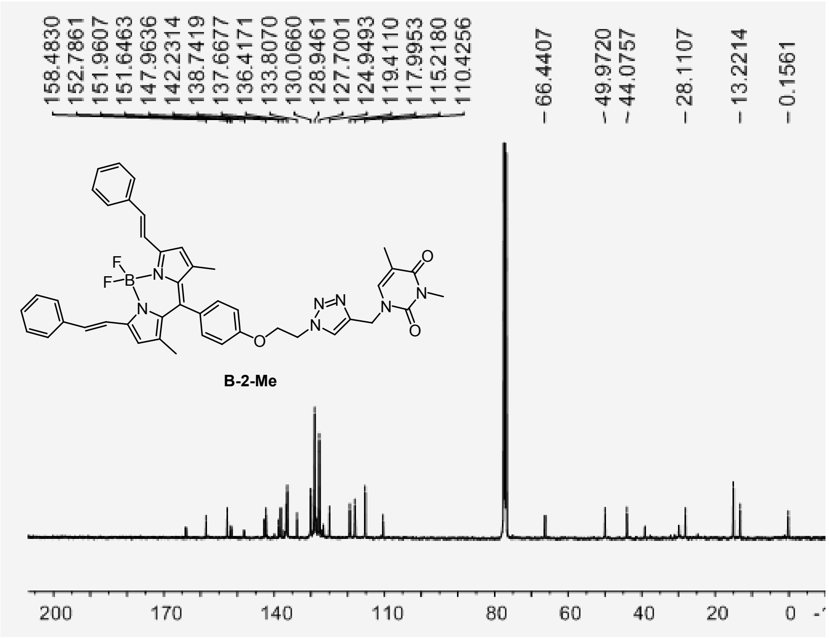

ppm

Fig. S37 ^{13}C NMR of **B-2-Me** (100 MHz, $CDCl_3$).

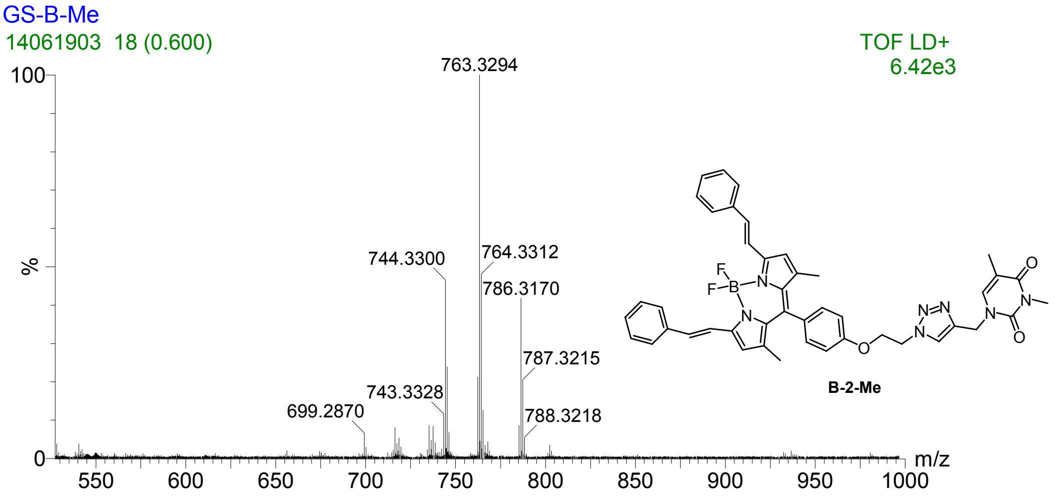

Fig. S38 TOF HRMS MALDI of **B-2-Me**.

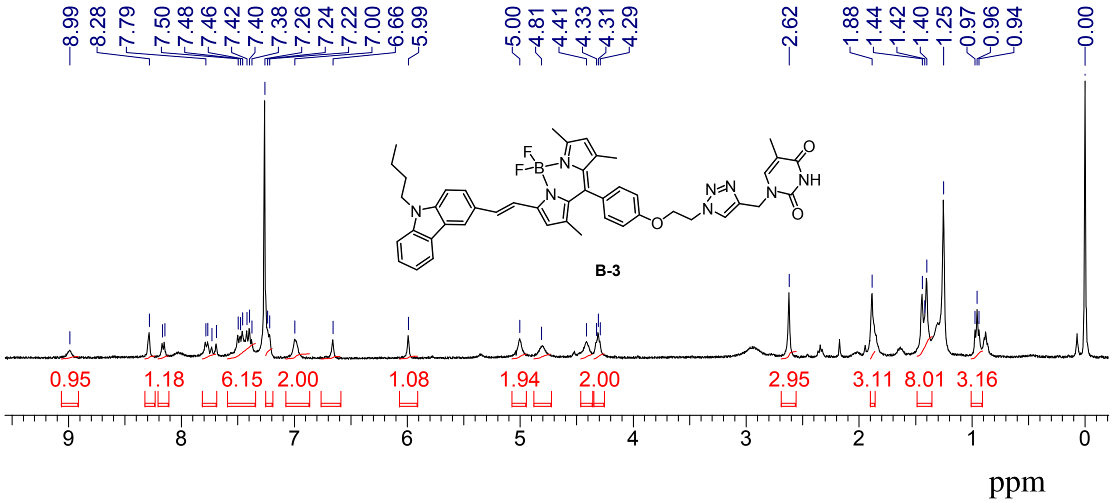

Fig. S39 ^{1}H NMR of **B-3** (500 MHz, $CDCl_3$).

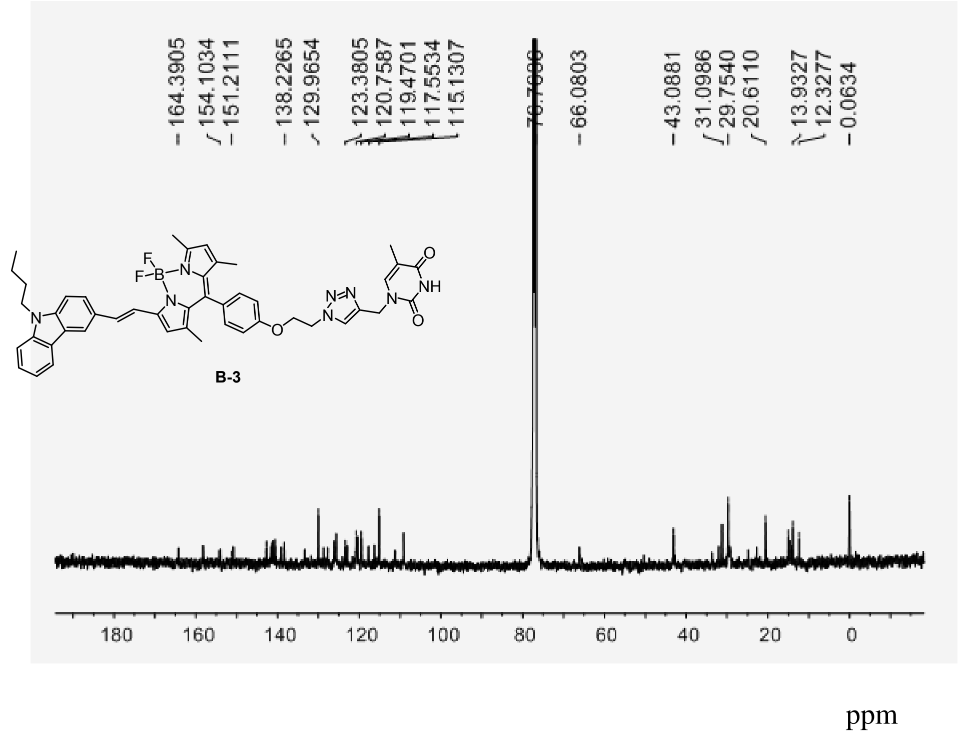

Fig. S40. ^{13}C NMR of **B-3** (100 MHz, $CDCl_3$).

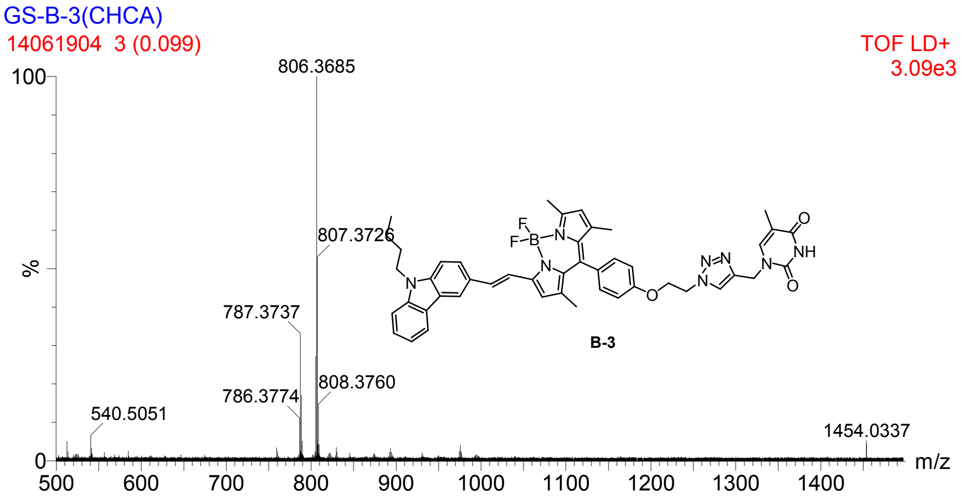

Fig. S41 TOF HRMS MALDI of **B-3**.

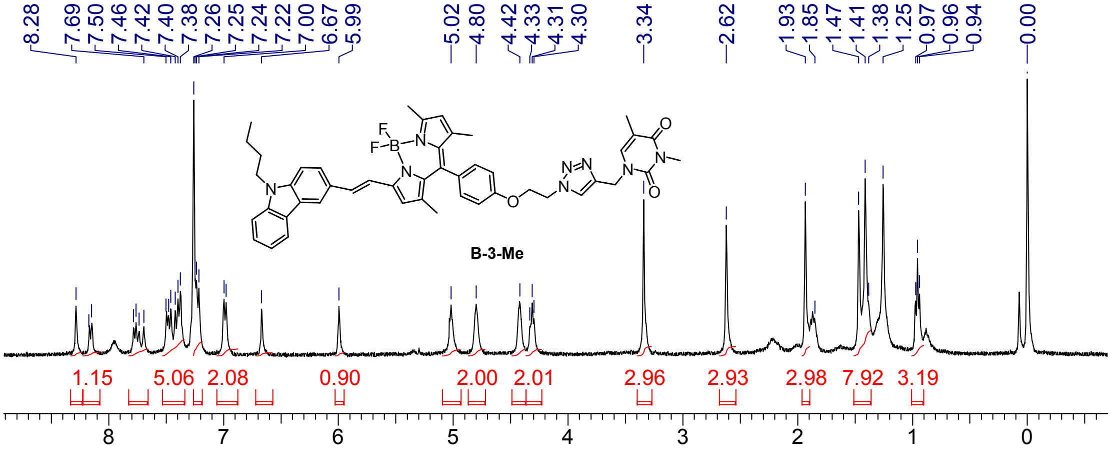

Fig. S42 ^{1}H NMR of **B-3-Me** (500 MHz, $CDCl_3$).

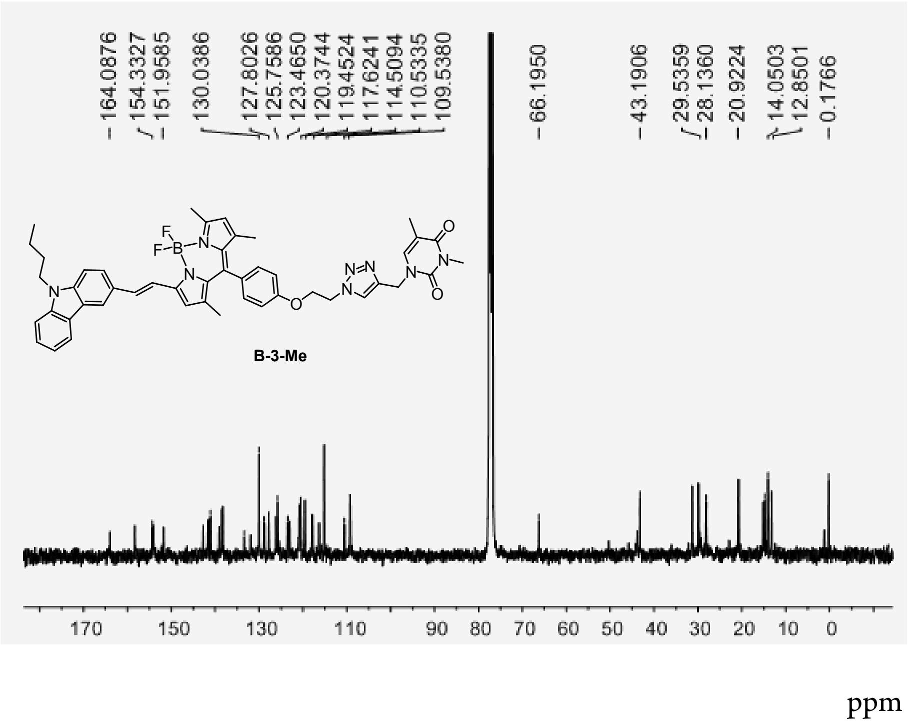

Fig. S43. ^{13}C NMR of **B-3-Me** (100 MHz, $CDCl_3$).

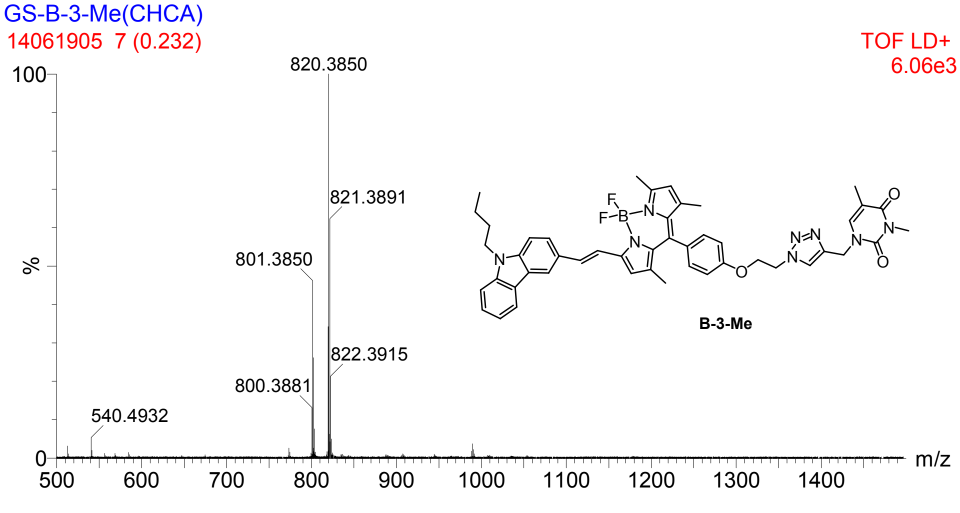

Fig. S44. TOF HRMS MALDI of **B-3-Me**.

2.0 Synthetic details

Synthesis of Compound 2. 2-Chloro-acetyl chloride (0.36 mL, 4.6 mmol) was added dropwise into the solution of 2-amino-6-acetylamidopyridine (0.7 g, 4.6 mmol) in dry CH_2Cl_2 (30 mL) during 10 min, and then the mixture was kept at room temperature (RT) for 2 h. The resulted solution was poured into saturated aqueous Na_2CO_3 solution (30 mL). The CH_2Cl_2 layer was separated and washed with saturated aqueous NaCl solution. The organic layer was dried over Na_2SO_4. Solvent was removed under reduced pressure. A white solid was obtained. Yield:). Yeild: 492.0 mg (46.8%). ^{1}H NMR (400 MHz, CD_3COCD_3): 9.25 (s, 1H), 7.95−7.75 (m, 3H), 4.34 (s, 2H), 2.05 (s, 3H). TOF HRMS ESI$^+$: *m*/*z* calcd for $[C_9H_{10}N_3O_2Cl]^+$: 227.0462; found: 227.0461.

Synthesis of Compound 3. NaN_3 (45.5 mg, 0.7 mmol) and compound **2** (113.5 mg, 0.5 mmol) was dissolved in DMF (15 mL). The mixture was stirred at 70 °C for 5 h. Then the reaction mixture was washed with water (3× 50 mL). The mixture solution was extracted with dichloromethane (2× 30 mL). The organic layer was dried over Na_2SO_4. Solvent was removed under reduced pressure. White solid was obtained. Yeild: 103.2 mg (88.2%). ^{1}H NMR (500 MHz, $CDCl_3$): 7.90−7.79 (m, 3H), 4.19 (s, 2H), 2.26 (s, 3H). TOF HRMS ESI$^+$: *m*/*z* calcd for $[C_9H_{10}N_6O_2+Na]^+$:257.0763; found: 257.0762.

Synthesis of Compound 5. A mixture of *p*-hydroxybenzaldehyde (2.44 g, 20 mmol), 3-bromo-1-propyne (3.54 g, 30 mmol) and potassium carbonate (4.15 g, 30 mmol) were dissolved in ethanol (150 mL), then the reaction mixture was refluxed for 6 h. The mixture was concentrated under reduced pressure. The residul was purified by column chromatography (silica gel, CH_2Cl_2). Yield: 2.88 g (60.0 %). ^{1}H NMR (400 MHz, $CDCl_3$): 9.91 (s, 1H), 7.88−7.85 (m, 2H), 7.12−7.08 (m, 2H), 4.79 (d, 2H), 2.58 (t, 1H, J = 4.0 Hz). Mp 76.6−77.8 ºC. TOF HRMS ES$^+$: *m*/*z* calcd ($[C_{10}H_8O_2+H]^+$): 161.0597; found: 161.0594.

Synthesis of Compound 6. $NaBH_4$ (304 mg, 8 mmol) and compound **5** (500 mg, 3.1 mmol) were mixed in THF/CH_3OH(V/V=1/1, 50 mL). The mixture was stirred at RT for 5 min. Then the solvent was evaporated under reduced pressure. The crude product was purified by column chromatography (silica gel, CH_2Cl_2). Colourless oil was obtained. Yield: 0.48g (81.8 %). ^{1}H NMR (500 MHz, $CDCl_3$): 7.30 (d, 2H, J = 8.5 Hz), 6.97 (d, 2H, J = 8.7 Hz), 4.68 (s, 2H), 4.60 (s, 2H), 2.51 (s, 1H), 1.91 (s, 2H).

Synthesis of Compound 7. Compound **6** (0.21 mg, 1.3 mmol) was dissolved in CH_2Cl_2 (20 mL) and pyridine (270 μL, 2.6 mmol) was added under Ar atmosphere. The mixture was cooled on an ice bath and a solution of methyl malonyl chloride (270 μL, 2.6 mmol) in CH_2Cl_2 (10 mL) was added dropwise. The mixture was stirred for 12 h at RT. The crude product was purified by column chromatography (silica gel, CH_2Cl_2) to afford of compound 6 as colourless oil. Yield: 303.7 mg (84.6 %). ^{1}H NMR (500 MHz, $CDCl_3$): 7.32 (d, 2H, J = 8.5 Hz), 6.97 (d, 2H, J = 8.7 Hz), 5.12 (s, 2H), 4.69 (s, 2H), 4.20−4.16 (m, 2H), 3.39 (s, 2H,), 2.52 (s, 1H), 1.24 (t, 2H, J = 7.0 Hz). TOF HRMS EI$^+$: m/z calcd ($[C_{15}H_{16}O_5]^+$): 276.0998; found: 276.0990.

Synthesis of Compound 8. Iodine (62 mg，0.25 mmol) were added to a solution of C_{60} (182.6 mg, 0.27 mmol) in toluene (60 mL). A solution of compound **7** in toluene (150 mL) was added and the mixture was degassed with Ar. A solution of 1,8-diazabicyclo[5.4.0]undec-7-ene (DBU) (72 μL, 0.27 mmol) in toluene (20 mL) was added dropwise within one hour and the reaction mixture was stirred over night. The crude product was purified with column chromatography (silica gel, DCM) to yield 126.6 mg of gray soild, Yield: 51 %. ^{1}H NMR (500 MHz, $CDCl_3$): 7.47 (d, 2H, J = 8.6 Hz), 7.01 (d, 2H, J = 8.7 Hz), 5.47 (s, 2H), 4.71 (s, 2H), 4.50−4.46 (m, 2H), 2.52 (s, 1H), 1.38 (t, 2H, J = 7.1 Hz). HRMS (MALDI): m/z calcd for $[C_{75}H_{14}O_5]^+$: 994.0841; found: 994.0844.

Synthesis of Compound 9. Under Ar atmosphere, *p*-hydroxybenzaldehyde (4.88 g, 40 mmol) and anhydrous K_2CO_3 (11.06 g, 80 mmol) were mixed in dry ethanol (150 mL). The mixture was stirred at 70 ºC for 0.5 h. The mixture was then cooled to RT and 1,2-dibromoethane (14.9 g, 80 mmol) was added using syringe. The mixture was stirred at 79 °C for 8 h. The mixture was then cooled to RT. K_2CO_3 was removed by filtration and the filtrate was evaporated to dryness under reduced pressure. The product was purified by column chromatography (silica gel, CH_2Cl_2) to afford colourless oil. Yield: 4.32 g (47.2%). ^{1}H NMR (400 MHz, $CDCl_3$): 9.90 (s, 1H), 7.87−7.85 (m, 2H), 7.04−7.01 (m, 2H), 4.40 (t, 2H, J = 12.0 Hz), 3.68 (t, 2H, J = 12.0 Hz). TOF HRMS ESI$^+$: m/z calcd for $[C_9H_9BrO_2+H]^+$: 228.9859; found: 228.9688.

Synthesis of Compound 10. A mixture of compound **8** (1.65g, 7.19 mmol) and NaN_3 (0.6 g, 9.46 mmol) in DMF (20 mL) was heated at 100 ºC. After 10 h, the mixture was poured to water . The aqueous phased was extracted with CH_2Cl_2. The solvent was evaporated under reduced pressure and the azide compound was obtained as colourless oil. Yield: 1.36 g (99 %). The compound was used without any further purification.

Synthesis of Compound 11. Under N_2 atmosphere, a mixture of compound **10** (859.5 mg, 4.5 mmol) and 2,4-dimethylpyrrole (2.0 mL, 945 mg, 9.0 mmol) in dry CH_2Cl_2 (100 mL) was stirred at RT. Under ice-cold condition, trifluoracetic acid (TFA. 0.1 mL) was added. The mixture was stirred at RT over night. A solution of DDQ (1.02 g, 4.5 mmol) in THF (40 mL) was added, then the mixture was stirred at RT for 7 h. Triethylamine (13.5 mL) was added into the mixture dropwise with ice bath cooling, and the mixture was stirred for 0.5 h. $BF_3{\bullet}Et_2O$ (13.5 mL) was added dropwise via syringe. The reaction mixture was stirred at RT over night. The solution was concentrated under reduced pressure and the residual was poured into water (200 mL), the mixture was stirred for 24 h. The mixture was extract with CH_2Cl_2 and the organic layer was dried over anhydrous $MgSO_4$. The solvent was evaporated under reduced pressure. The crude product was purified using column chromatography (silica gel, CH_2Cl_2 : hexane = 1:1, v/v) to give **11** as red power . Yield: 214.5 mg (11.6%). ^{1}H NMR (400 MHz, $CDCl_3$): 7.21 (d, 2H, J = 12.0 Hz), 7.04 (d, 2H, J = 8.0 Hz), 5.98 (s, 2H,), 4.21 (t, 2H, J = 8.0 Hz), 3.66 (t, 2H, J = 8.0 Hz), 2.55 (s, 6H), 1.43 (s, 6H). TOF HRMS ESI$^+$: m/z calcd for $[C_{21}H_{22}BF_2N_5O+H]^+$: 410.2073; found: 410.1938.

Synthesis of Compound 12. Compound **11** (40 mg, 0.1 mmol) was dissolved in dry DMF (5 mL). Then benzaldehyde (42.4 mg 0.4 mmol) was added, followed by acetic acid (3 drops) and piperidine (3 drops). Under Ar atmosphere, the mixture was subjected to microwave irradiation (8 min, 150 °C). Then the solution was poured into ice water, and the mixture was extracted with CH_2Cl_2. The organic layer was dried over Na_2SO_4 and evaporated to dryness. The residule was purified by column chromatography (silica gel, ethyl acetate/hexanes, 1/4, v/v) to give a blue solid. Yield: 12.3 mg (21.0 %). ^{1}H NMR (500 MHz, $CDCl_3$): 7.76 (s, 1H), 7.73 (s, 1H), 7.64 (d, 4H, J = 7.4 Hz), 7.42–7.39 (m, 4H),

7.34–7.24 (m, 6H), 7.06 (d, 2H, *J* = 7.6 Hz), 6.65 (s, 2H), 4.23 (t, 2H, *J* = 4.9 Hz), 3.68 (t, 2H, *J* = 4.8 Hz), 1.51 (m, 5H). TOF HRMS (MALDI) ESI$^+$: *m/z* calcd for $[C_{35}H_{30}N_5OBF_2]^+$: 585.2511; found: 585.2516.

Synthesis of Compound 14. A mixture of carbazole (1.161 g, 6.95 mmol) and NaH (0.695 g, 17.35 mmol, 60% oil dispersion) in dry DMF (10 mL) was stirred at RT for 1 h. n-Butyl bromide (0.472 g, 3.47 mmol) was added, and the mixture was stirred for 1 h. The mixture was then poured into water (150 mL), and the precipitate was filtered, dissolved in dichloromethane (50 mL), dried over Na_2SO_4, and evaporated to dryness. The crude product was purified by column chromatography (silica gel, petroleum ether), to give **14** as white solid. Yield: 1.47 g (95%). ^{1}H NMR (400 MHz, $CDCl_3$): 8.10 (d, 2H, *J* = 8 Hz), 7.44 (d, 1H, *J* = 7.2 Hz), 7.39 (d, 2H, *J* = 8 Hz), 7.21 (t, 2H, *J* = 7.2 Hz), 4.27 (t, 2H, J = 7.2 Hz), 1.87−1.79 (m, 2H), 1.43−1.33 (m, 2H), 0.92–0.90 (m, 2H). TOF HRMS ESI$^+$: *m/z* calcd for $[C_{16}H_{17}N+H]^+$: 224.1434; found: 224.1435.

Synthesis of Compound 15. $POCl_3$ (4.2 mL) was added dropwise into DMF (2 mL) while the mixture was cooled with ice-bath. Then the temperature of the mixture was warmed to RT. The reaction mixture was stirred at RT for 2 h. 9-Butyl-9*H*-carbazole (2.00 g, 8.96 mmol) was added into the reaction mixture at 60 ℃. After 6 h, the reaction was quenched with saturated $NaHCO_3$ solution (100 mL). Then the mixture was stirred for 1 h. The precipitate was collected with filtration and the solid was washed with cold ethanol (50 mL). The crude product was purified by column chromatography (silica gel, CH_2Cl_2 : petroleum ether = 1:1, v/v) to give the 9-butyl-9H-carbazole-3-carbaldehyde as colourless oil. Yield: 1.58 g (70%). ^{1}H NMR (400 MHz, $CDCl_3$): 10.08 (s, 1H), 8.58 (s, 1H), 8.14 (d, 2H, *J* = 7.8 Hz), 8.00 (d, 2H, *J* = 8.5 Hz), 7.52 (t, 1H, *J* = 8.3 Hz), 7.45 (d, 2H, *J* = 8.6 Hz), 7.31 (t, 1H, *J* = 7.2 Hz), 4.30 (t, 2H, *J* = 7.1 Hz), 1.89–1.81 (m, 2H), 1.44–1.35 (m, 2H), 0.95 (t, 3H, *J* = 8.7 Hz). TOF HRMS ESI$^+$: *m/z* calcd for $[C_{17}H_{17}NO+H]^+$: 252.1383; found: 252.1381.

Synthesis of Compound 16. Compound **15** (69 mg, 0.3 mmol) was dissolved in dry DMF (5 mL). Then compound **11** (109.8 mg 0.3 mmol) was added, followed by acetic acid (10 drops) and piperidine (10 drops). The mixture was put under an Ar atmosphere before it was subjected to microwave

irradiation (5 min, 150 °C). After the microwave irradiation, the solution was poured into ice water and the mixture was extracted with CH_2Cl_2 (3×30 mL). The organic layer was dried over Na_2SO_4 and evaporated to dryness, the mixture was purified by column chromatography (silica gel, ethyl acetate/hexanes, 1/15, v/v) to give blue solid. Yield: 42 mg (22.1 %). ^{1}H NMR (400 MHz, $CDCl_3$): 8.29 (s, 1H), 8.17 (d, 1H, *J* = 7.8 Hz), 7.78−7.70 (m, 2H), 7.48 (t, 2H, *J* = 8.3 Hz), 7.40 (t, 2H, *J* = 8.8 Hz), 7.28−7.22 (m, 3H), 7.06 (d, 1H, *J* = 8.7 Hz), 6.67 (s, 1H), 6.00 (s, 1H), 4.31 (t, 2H, *J* = 7.1 Hz), 4.22 (t, 2H, *J* = 5.0 Hz), 3.67 (t, 2H, *J* = 5.0 Hz), 2.63 (s, 3H), 1.91−1.83 (m, 2H), 1.51 (s, 3H), 1.45 (s, 3H), 1.42−1.38 (m, 2H), 0.96 (t, 3H, *J* = 7.4 Hz). HRMS (MALDI): *m/z* calcd for $[C_{38}H_{37}N_6OF_2B]^+$: 642.3065; found: 642.3090.

Synthesis of Compound 17. Under Ar atmosphere, thymine (500 mg, 4 mmol) and K_2CO_3 (828 mg, 6 mmol) were dissolved in dry DMF (6 mL). Then a solution of propargyl bromide (1.19 g 10 mmol) in *n*-hexane (0.8 mL) was added dropwise. The reaction mixture was stirred over night. The mixture was poured into ice water. The mixture was extracted with CH_2Cl_2, dried over Na_2SO_4 and evaporated to dryness. The mixture was purified by column chromatography (silica gel, ethyl acetate/hexanes, 1/2, v/v) to give a white solid. Yield: 123.3 mg (18.8 %). ^{1}H NMR (400 MHz, $CDCl_3$): 9.04 (s, 1H), 7.25 (s, 1H), 4.55 (d, 2H, *J* = 29.5 Hz), 2.47 (t, 1H, *J* = 2.5 Hz), 1.96 (s, 3H). TOF HRMS ESI$^+$: *m/z* calcd for $[C_8H_8N_2O]^+$:164.0586; found: 164.0593.

Synthesis of Compound 19. Compound **18** (100 mg, 0.61 mmol) was dissolved in dry DMF (5 mL). Then NaH (152 mg 1.3 mmol) was added under ice-bath cooling, followed by lithium bromide (156.6 mg, 1.8 mmol). The mixture was kepted in ice-bath for 20 min. Then methyl iodide (260 mg, 1.8 mmol) was added and the mixture was stirred at RT overnight. The solution was poured into ice water. The mixture was extracted with CH_2Cl_2, the organic layer was dried over Na_2SO_4 and evaporated to dryness. The crude product was purified by column chromatography (silica gel, dichloromethane/methnol, 40/1, v/v) to give white solid. Yield: 50.8 mg (46.7 %). ^{1}H NMR (500 MHz, $CDCl_3$): 7.24 (s, 1H), 4.57 (s, 2H), 3.37 (s, 3H), 2.45 (s, 1H), 1.97 (s, 3H,). TOF HRMS ESI$^+$: *m/z* calcd for $[C_9H_{10}N_2O_2]^+$: 178.0472; found: 178.0473.

3.0 Abosption and Emssion details

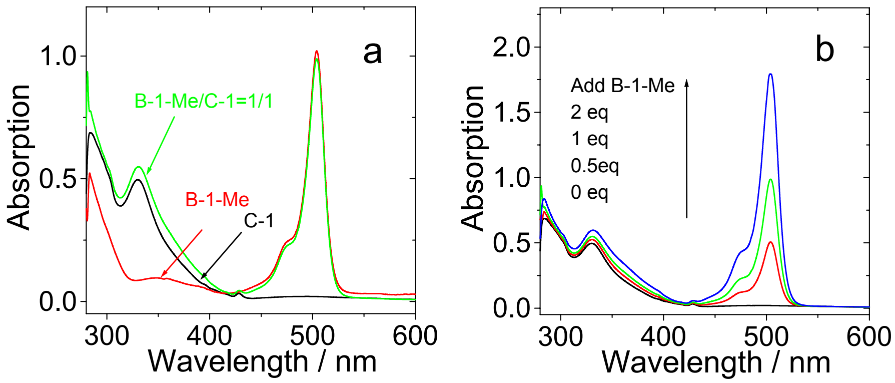

Fig. S45 UV-vis absorption spectra of (a) **B-1-Me**, **C-1** and **C-1/B-1-Me**=1/1, (b) **C-1** solution (1.0×10^{-5} M) in toulene with increasing of **B-1-Me**. c = 1.0×10^{-5} M in toluene. 20 °C.

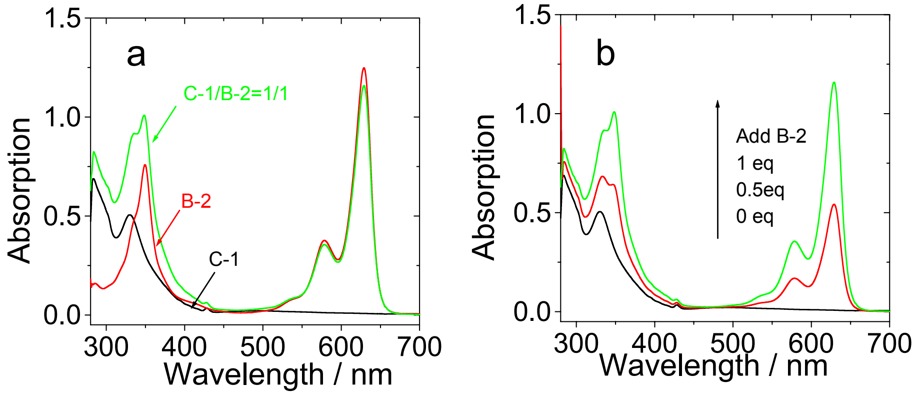

Fig. S46 UV-vis absorption spectra of (a) **B-2**, **C-1** and **C-1/B-2**=1/1, (b) **C-1** solution (1.0×10^{-5} M) in toulene with increasing of **B-2**. c = 1.0×10^{-5} M in toluene. 20 °C.

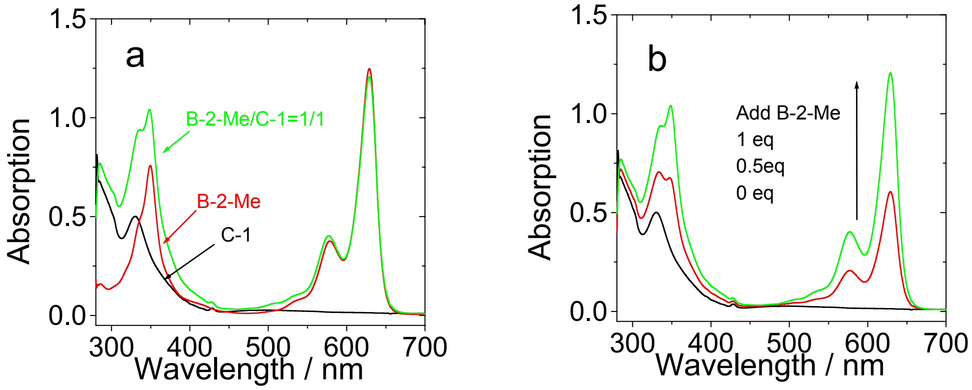

Fig. S47 UV-vis absorption spectra of (a) **B-2-Me**, **C-1** and **C-1/B-2-Me**=1/1, (b) **C-1** solution (1.0 × 10^{-5}M) in toulene with increasing of **B-2-Me**. c = 1.0×10^{-5} M in toluene. 20 °C.

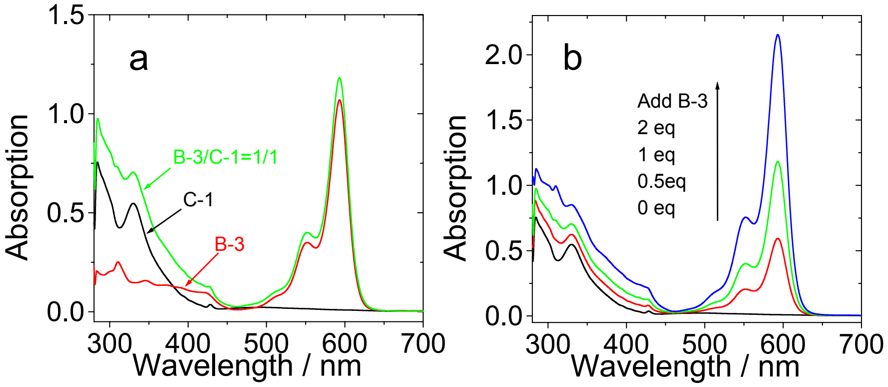

Fig. S48 UV-vis absorption spectra of (a) **B-3**, **C-1** and **C-1/B-3** = 1/1, (b) **C-1** solution (1.0 × 10^{-5} M) in toulene with increasing of **B-3**. c = 1.0 × 10^{-5} M in toluene. 20 °C.

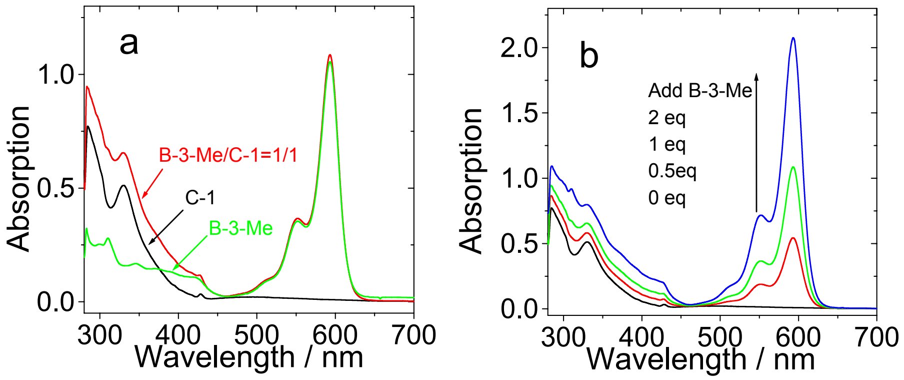

Fig. S49 UV-vis absorption spectra of (a) **B-3-Me**, **C-1** and **C-1/ B-3-Me** =1/1, (b) **C-1** solution (1.0 × 10^{-5}M) in toulene with different concentration **B-3-Me** solution. c = 1.0×10^{-5} M in toluene. 20 °C.

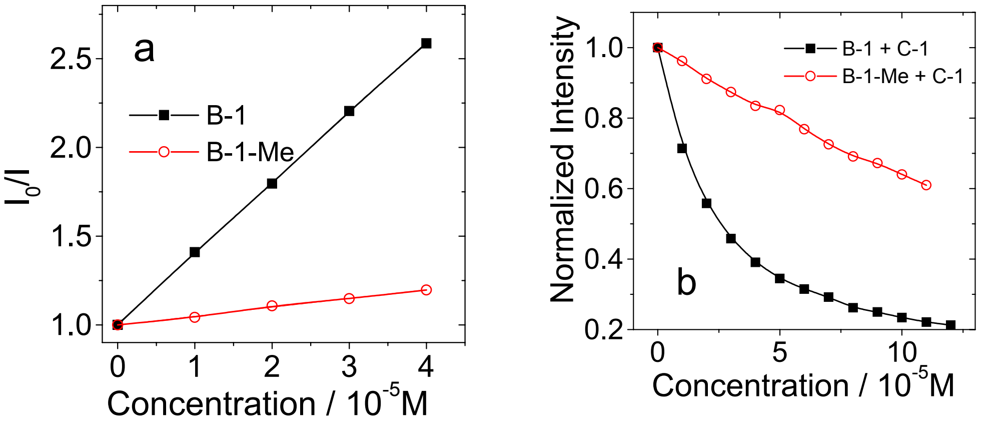

Fig. S50 (a) **B-1** and **B-1-Me** dependence of I_0/I on the concentration of **C-1**; (b) Emission intensity of **B-1** and **B-1-Me** at constant concentration of 10^{-5}M in toluene, *versus* increasing concentration of [**C-1**] at 517 nm following excitation at 475 nm.

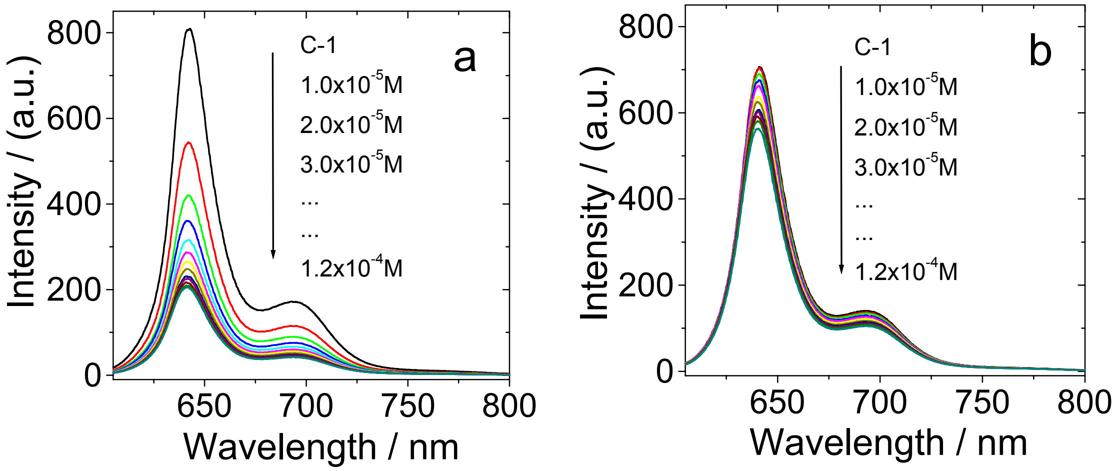

Fig. S51 Fluorescence of (a) **B-2** and (b) **B-2-Me** solution (1.0×10^{-5}M) in toluene quenched by **C-1** solution in toluene (λ_{ex} = 600 nm).

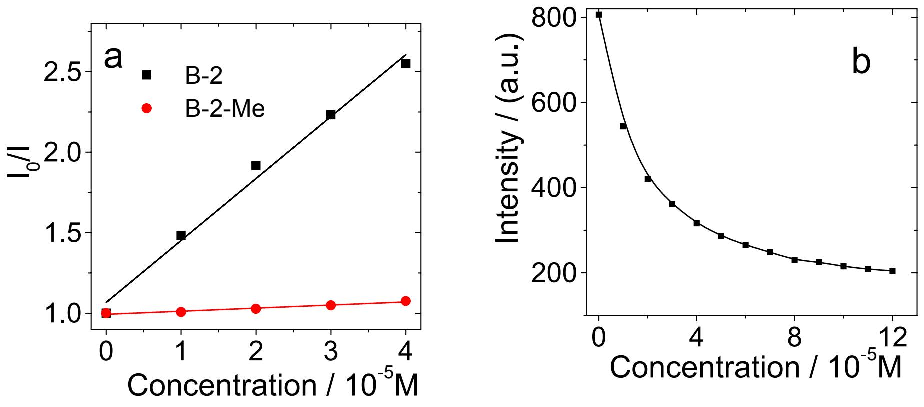

Fig. S52 (a) **B-2** and **B-2-Me** dependence of I_0/I on the concentration of **C-1**, (b) Emission intensity of **B-2** at constant concentration of 10^{-5}M in toluene, *versus* increasing concentration of [C-1] at 642 nm following excitation at 600 nm.

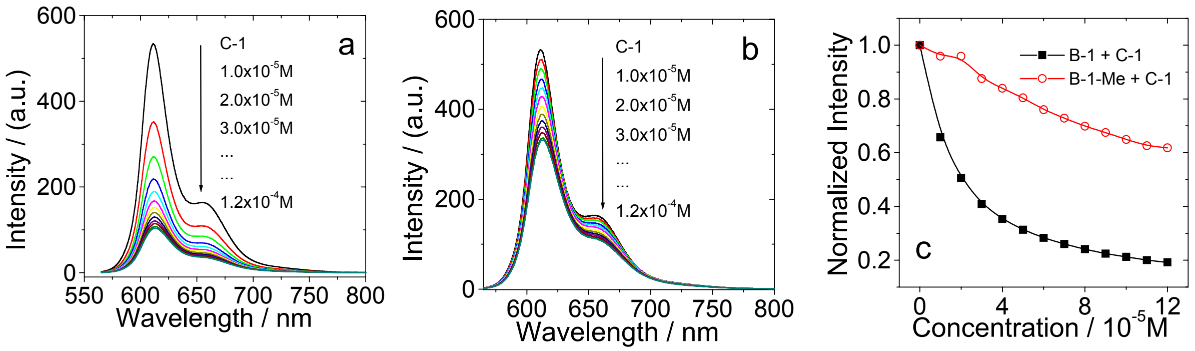

Fig. 53 Fluorescence titration (a) **B-3** and (b) **B-3-Me** solution (1.0×10^{-5}M) in toulene quenched by **C-1** solution in toluene (λ_{ex} = 560 nm). (b) Emission intensity of **B-3** at constant concentration of 10^{-5}M in toluene, *versus* increasing concentration of [**C-1**] at 611 nm following excitation at 560 nm.

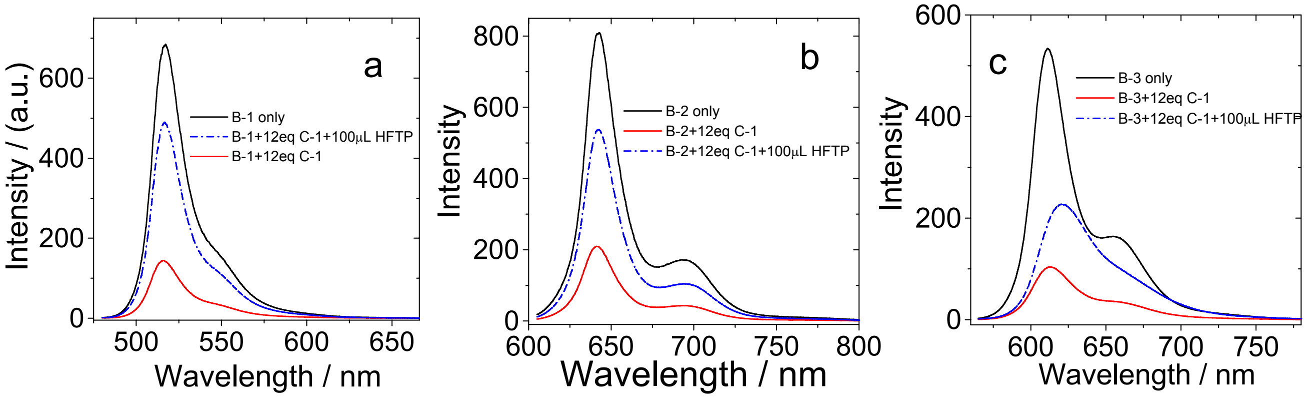

Fig. S54 Fluorescence of (a) **B-1**, **B-1** + 12eq **C-1** + 100 μL HFTP and **B-1** + 12eq **C-1** (λ_{ex} = 475 nm), (b) **B-2**, **B-2** + 12eq **C-1** + 100 μL HFTP and **B-2** + 12eq **C-1** (λ_{ex} = 600 nm), (c) **B-3**, **B-3** + 12eq **C-1** + 100 μL HFTP and **B-3** + 12eq **C-1** (λ_{ex} = 560 nm). In toluene. 20 °C.

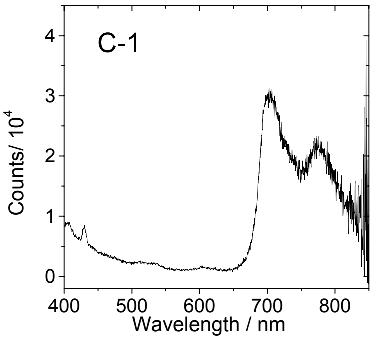

Fig. S55 The emssion spectra of **C-1**. c = 1.0 × 10^{-5} M in toluene (λ_{ex} = 331 nm). 20 °C.

4.0 Transient absorption details

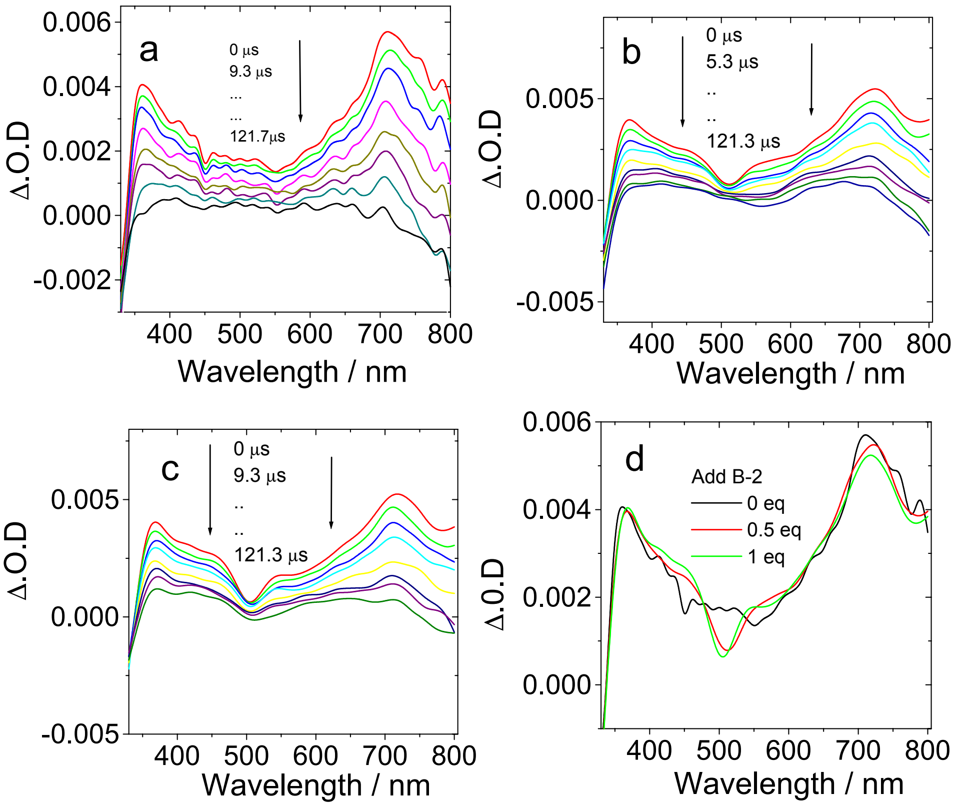

Fig. S56 Nanosecond time-resolved transient absorption difference spectra with different delay times of compound **C-1** with adding compound **B-1-Me**. (a) 0 eq **B-1-Me**, (b) 0.5 eq **B-1-Me**, (c) 1 eq **B-1-Me**. (d) In different concentration of **B-1-Me** with delay times at 0 μs. C = 1.0×10^{-5} M, 20 °C. In toluene after pulsed excitation at 495 nm under N_2.

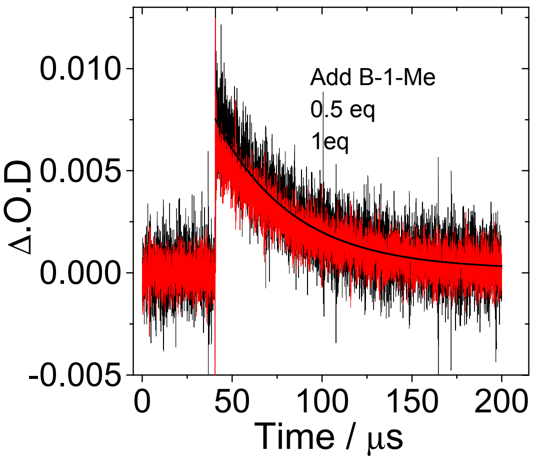

Fig. S57 Nanosecond time-resolved transient absorption difference spectra of compound **C-1** with adding compound **B-1-Me**. Decay trace at 720 nm. In toluene after pulsed excitation at 495 nm under N_2. 0eq: 42.8 μs, 0.5eq: 42.8 μs, 1 eq: 46.3 μs.

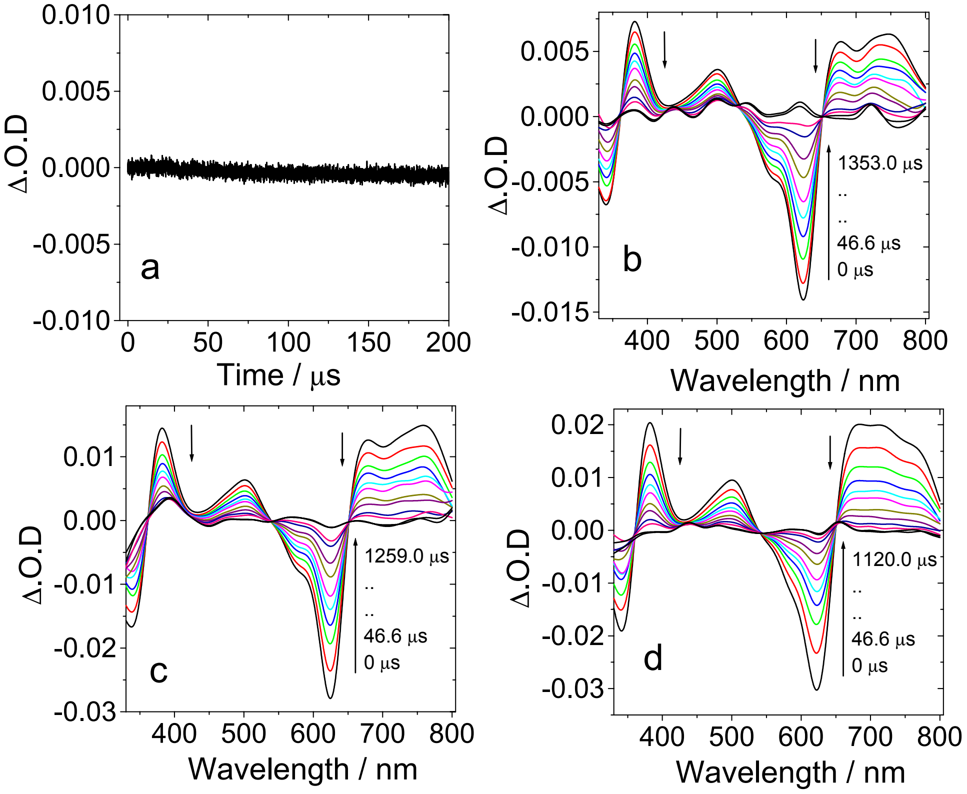

Fig. S58 Nanosecond time-resolved transient absorption difference spectra with different delay times of compound **C-1** with adding compound **B-2**. (a) 0 eq **B-2**, (b) 0.25 eq **B-2**, (c) 0.5 eq **B-2**, (d) 1 eq **B-2**. C = 1.0×10^{-5} M, 20 °C. In toluene after pulsed excitation at 620 nm under N_2.

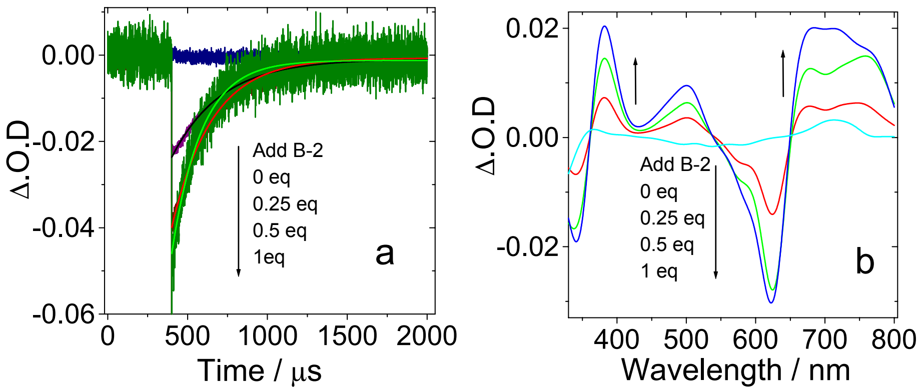

Fig. S59 Nanosecond time-resolved transient absorption difference spectra of compound **C-1** with adding compound **B-2**. (a) Decay trace at 630 nm, (b) with delay times at 0 μs. C = 1.0×10^{-5} M, 20 °C. In toluene after pulsed excitation at 620 nm under N_2.

0 eq: - μs, 0.25eq: 312.4 μs, 0.5 eq: 250.7 μs, 1 eq: 185.5 μs.

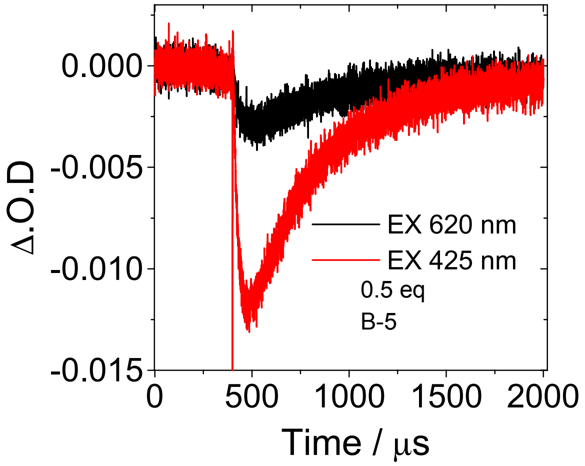

Fig. S60 Nanosecond time-resolved transient absorption decay trace at 630 nm of mixture solution of **C-1** and 0.5eq **B-2-Me**. 20 °C. c = 1.0×10^{-5} M, In toluene after pulsed excitation at 425nm and 620 nm under N_2.

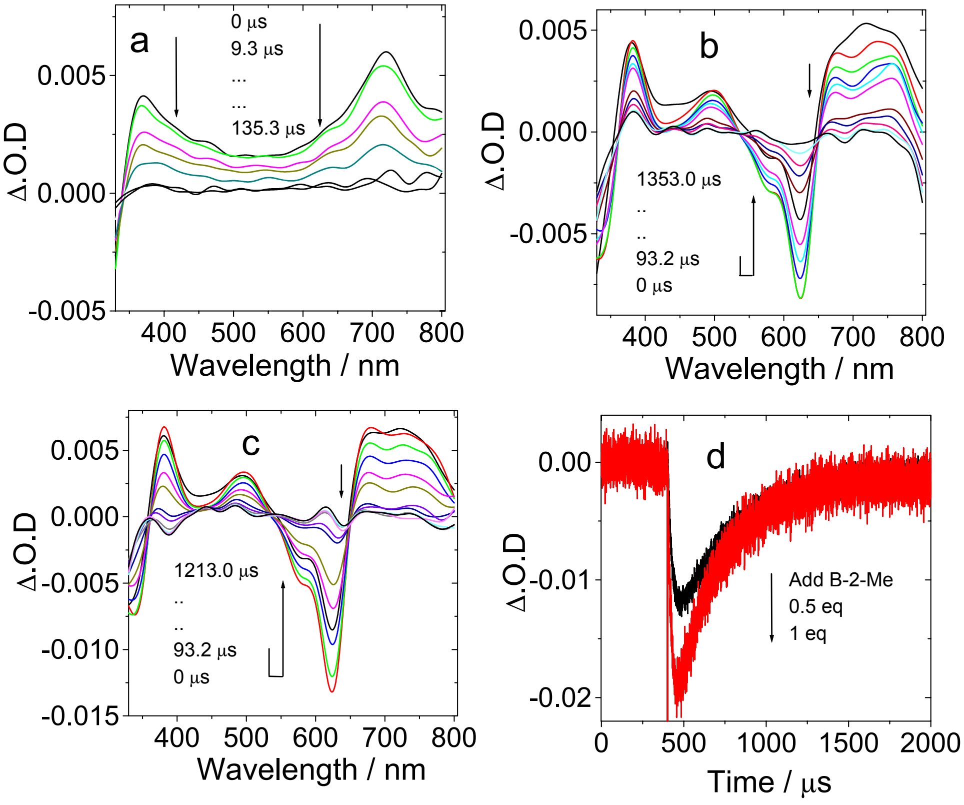

Fig. S61 Nanosecond time-resolved transient absorption difference spectra with different delay times of compound **C-1** with adding compound **B-2-Me**. (a) 0 eq **B-2-Me**, (b) 0.5 eq **B-2-Me**, (c) 1 eq **B-2-Me**, (d) Decay trace at 630 nm. c = 1.0×10^{-5} M, 20 °C. In toluene after pulsed excitation at 425 nm under N_2.

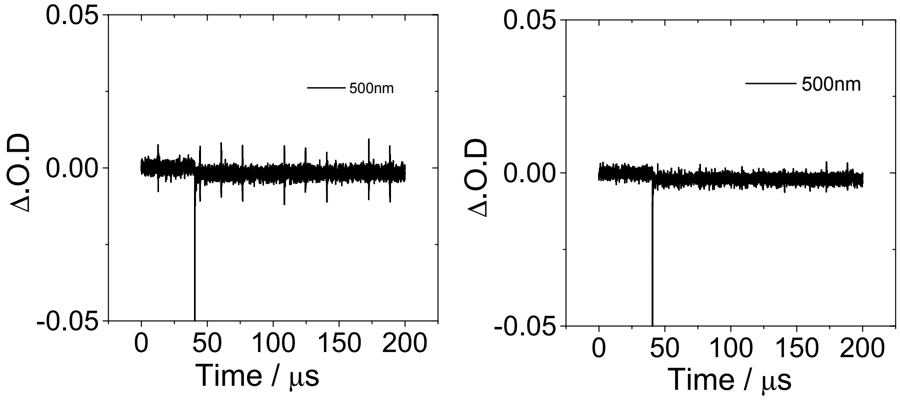

Fig. S62 Nanosecond time-resolved transient absorption decay trace at 500 nm: (a) **B-1**, (b) **B-1-Me**. 20 °C. c = 1.0×10^{-5} M, In toluene after pulsed excitation at 495 nm under N_2.

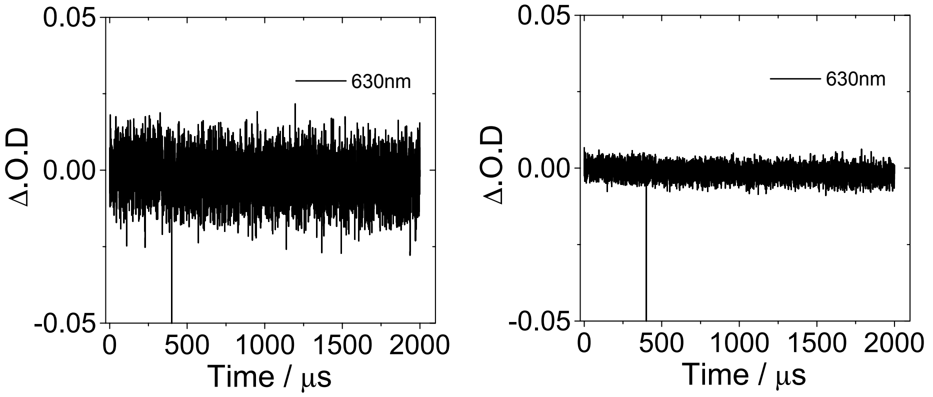

Fig. S63 Nanosecond time-resolved transient absorption decay trace at 630 nm: (a) **B-2**, (b) **B-2-Me**. 20 °C. c = 1.0×10^{-5} M, In toluene after pulsed excitation at 620 nm under N_2.

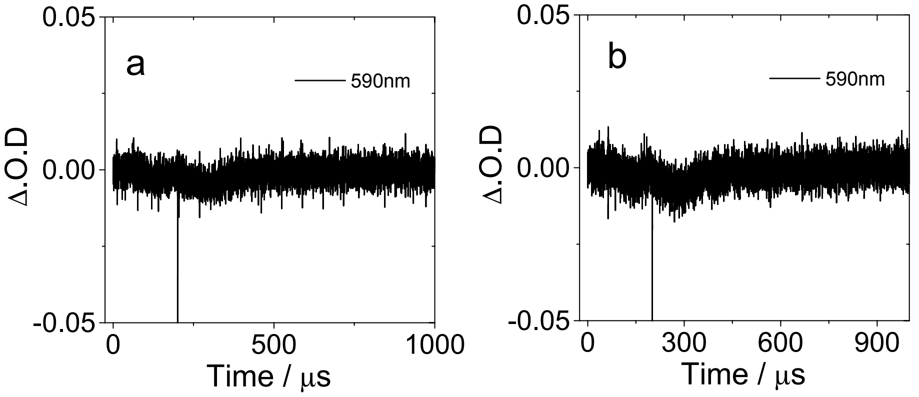

Fig. S64 Nanosecond time-resolved transient absorption decay trace at 593 nm: (a) **B-3**, (b) **B-3-Me**. 20 °C. c = 1.0×10^{-5} M, In toluene after pulsed excitation at 590 nm under N_2.

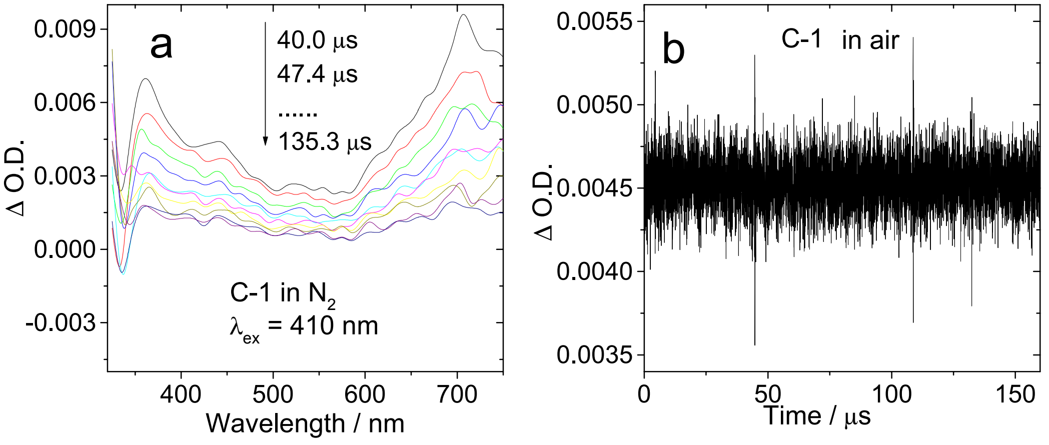

Fig. S65. Nanosecond time-resolved transient absorption difference spectra and decay curves of compounds. (a) **C-1** under nitrogen. (b) Decay curve of **C-1** at 720 nm in air, after pulsed excitation at 410 nm. the absorption for **C-1** at 410 nm is 0.096 in toluene, 20 °C.

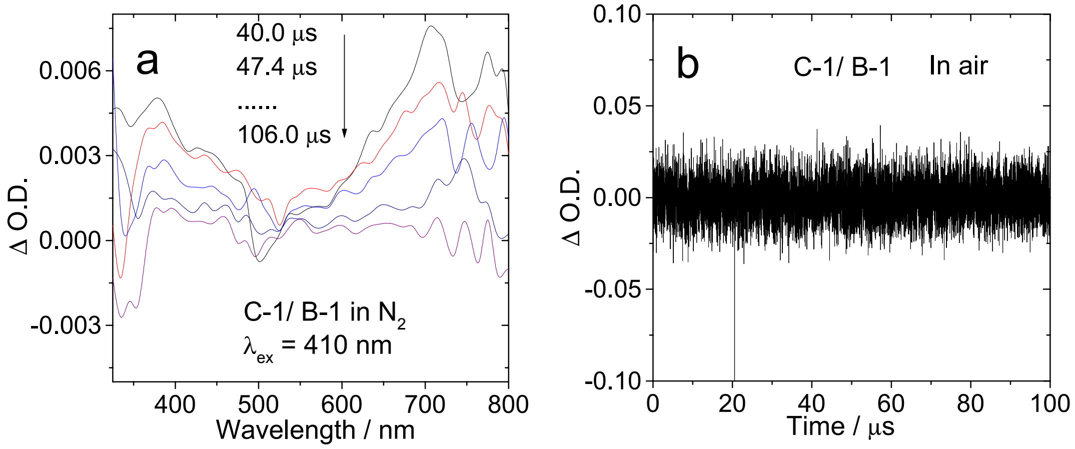

Fig. S66. Nanosecond time-resolved transient absorption difference spectra and decay curves of compounds. (a) **C-1/B-1**(**$c_{c\text{-}1}$:$c_{B\text{-}1}$**= 1:1) under nitrogen. (b) Decay curve of **C-1/B-1**(**$c_{c\text{-}1}$:$c_{B\text{-}1}$**= 1:1) at 720 nm in air, after pulsed excitation at 410 nm. the absorption for **C-1/B-1**(**$c_{c\text{-}1}$:$c_{B\text{-}1}$**= 1:1) at 410 nm is 0.096 in toluene, 20 °C.

5.0 The detailed data of electrochemistry.

Calculation of the free energy changes of the electron transfer

The free energy changes of the electron transfer process (charge seperation, CS), can be calculated with the Weller equation (eq. 1 and eq.2).

$$\Delta G^{0}{}_{\mathrm{CS}} = e[E_{OX} - E_{RED}] - E_{00} + \Delta G_{\mathrm{S}} \qquad \text{(Eq. 1)}$$

$$\Delta G_{\mathrm{S}} = -\frac{e^2}{4\pi\varepsilon_{\mathrm{S}}\varepsilon_0 R_{\mathrm{CC}}} - \frac{e^2}{8\pi\varepsilon_0}\left(\frac{1}{R_D} + \frac{1}{R_{\mathrm{A}}}\right)\left(\frac{1}{\varepsilon_{REF}} - \frac{1}{\varepsilon_S}\right) \qquad \text{(Eq. 2)}$$

Where ΔG_S is the static Coulombic energy, which is desribed by eq. 2. e = electronic charge, E_{OX} = half-wave potential for mone-electron oxidation of the electron-donor unit, E_{RED} = half-wave potential for one-electron reduction of the electron-acceptor unit; note herein the anodic and cathodic peak potentials were used because in some cases the oxidation is iiriversible therefore the formal potential $E_{1/2}$ cannot be derived; E_{00} = energy level approximated with the fluorescence emission wavelength (for the singlet excited state), or T_1 state energy of photosensitizers. ε_S = static dielectric constant of the solvent, RCC = (xx Å) center-to-center separation distance determined by DFT optimization of the geometry, R_D is the radius of the BODIPY-based donor, R_A is the radius of the electron acceptor, ε_{REF} is the static dielectric constant of the solvent used for the electrochemical studies, ε_0 permittivity of free space(ε_0 = $8.854187817\times10^{-12}$F/m). The solvents used in the calculation of free energy of the electron transfer is DCM (ε = 9.1, 20 °C) and Toluene (ε = 2.4, 20 °C).

For **B-1** and **C-1** in the solution,

Due to **B-1** and **C-1** can develop Hydrogen bonding in the aprotic solvent, we proposed that the center-to-center separation distance between **B-1** and **C-1** is close to the distance of covalent linkage of **B-1** and **C-1**. Here RCC = (40 Å), RA = 4.1 Å, RD = 4.1 Å, was abtained from the ground state optimized by TDDFT calculation.

In DCM,

Based on these parameters, for **B-1** and **C-1** in DCM, ΔG_S is calculated as -0.04 eV.

With reference **C-1** as electron acceptor, the $\Delta G(CS) = 1.11 - (-0.70) - 1.56 - 0.04 = 0.21$ eV;

With reference **B-1** as electron donor, the $\Delta G(CS) = 1.11 - (-0.70) - 2.45 - 0.04 = -0.68$ eV;

In Toluene, ΔG_S is calculated as 0.92 eV.

With reference **C-1** as electron acceptor, the $\Delta G(CS) = 1.11 - (-0.70) - 1.56 + 0.92 = 1.17$ eV;

With reference **B-1** as electron donor, the $\Delta G(CS) = 1.11 - (-0.70) - 2.45 + 0.92 = 0.28$ eV;

For **B-1-Me** and **C-1** in the solution,

Due to **B-1-Me** and **C-1** cannot develop Hydrogen bonding in the aprotic solvent, we proposed that the center-to-center separation distance between **B-1** and **C-1** is infinity. Here RCC = $\propto$. (Refer to JPCA)

In DCM, ΔG_S is calculated as 0.00 eV.

Based on these parameters, for **B-1** and **C-1** in DCM, ΔG_S is calculated as 0 eV.

With reference **C-1** as electron acceptor, the $\Delta G(CS) = 1.11 - (-0.70) - 1.56 - 0.00 = 0.25$ eV;

With reference **B-1-Me** as electron donor, the $\Delta G(CS) = 1.11 - (-0.70) - 2.45 - 0.00 = -0.64$ eV;

In Toluene, ΔG_S is calculated as 1.08 eV.

With reference **C-1** as electron acceptor, the $\Delta G(CS) = 1.11 - (-0.70) - 1.56 + 1.08 = 1.33$ eV;

With reference **B-1-Me** as electron donor, the $\Delta G(CS) = 1.11 - (-0.70) - 2.45 + 1.08 = 0.44$ eV;

For **B-2** and **C-1** in the solution,

Due to **B-2** and **C-1**can develop Hydrogen bonding in the aprotic solvent, we proposed that the center-to-center separation distance between **B-2** and **C-1** is close to the distance of covalent linkage of **B-2** and **C-1**. Here RCC = (43.9 Å), RA = 4.1 Å, RD = 11.1 Å, was obtained from the ground state optimized by TDDFT calculation.

In DCM,

Based on these parameters, for **B-2** and **C-1** in DCM, ΔG_S is calculated as -0.036 eV.

With reference **C-1** as electron acceptor, the $\Delta G(CS) = 0.84 - (-1.04) - 1.56 - 0.036 = 0.28$ eV;

With reference **B-2** as electron donor, the ΔG(CS) = 0.84 – (–1.04) – 1.93-0.036 = -0.09 eV;

In Toluene, ΔG_S is calculated as 0.60 eV.

With reference **C-1** as electron acceptor, the ΔG(CS) = 0.84 – (–1.04) – 1.56+060 = 0.92 eV;

With reference **B-2** as electron donor, the ΔG(CS) = 0.84 – (–1.04) – 1.93+0.60 = 0.55 eV;

For **B-2-Me** and **C-1** in the solution,

Due to **B-2-Me** and **C-1** cannot develop Hydrogen bonding in the aprotic solvent, we proposed that the center-to-center separation distance between **B-2-Me** and **C-1** is infinity. Here RCC = ∝. (Refer to JPCA)

In DCM, ΔG_S is calculated as 0.00 eV.

Based on these parameters, for **B-2-Me** and **C-1** in DCM, ΔG_S is calculated as 0.00 eV.

With reference **C-1** as electron acceptor, the ΔG(CS) = 0.84 – (–1.04) – 1.56-0.00 = 0.32 eV;

With reference **B-2-Me** as electron donor, the ΔG(CS) = 0.84 – (–1.04) – 1.93-0.00 = -0.05 eV;

In Toluene, ΔG_S is calculated as 0.74 eV.

With reference **C-1** as electron acceptor, the ΔG(CS) = 0.84 – (–1.04) – 1.56+0.74 = 1.06 eV;

With reference **B-2-Me** as electron donor, the ΔG(CS) = 0.84 – (–1.04) – 1.93+0.74 = 0.69 eV;

For **B-3** and **C-1** in the solution,

Due to **B-3** and **C-1**can develop Hydrogen bonding in the aprotic solvent, we proposed that the center-to-center separation distance between **B-3** and **C-1** is close to the distance of covalent linkage of **B-3** and **C-1**. Here RCC = (49.5 Å), RA = 4.1 Å, RD = 13.5 Å, was obtained from the ground state optimized by TDDFT calculation.

In DCM,

Based on these parameters, for **B-3** and **C-1** in DCM, ΔG_S is calculated as -0.032 eV.

With reference **C-1** as electron acceptor, the ΔG(CS) = 0.74 – (–1.21) – 1.56-0.032 = 0.36 eV;

With reference **B-3** as electron donor, the ΔG(CS) =0.74 – (–1.21)– 2.09-0.032 = -0.17 eV;

In Toluene, ΔG_S is calculated as 0.58 eV.

With reference **C-1** as electron acceptor, the ΔG(CS) = 0.74 – (–1.21) – 1.56+0.58 = 0.97 eV;

With reference **B-3** as electron donor, the ΔG(CS) = 0.74 – (–1.21) – 2.09+0.58 = 0.44 eV;

For **B-3-Me** and **C-1** in the solution,

Due to **B-3-Me** and **C-1** cannot develop Hydrogen bonding in the aprotic solvent, we proposed that the center-to-center separation distance between **B-3-Me** and **C-1** is infinity. Here RCC = ∝. (Refer to JPCA)

In DCM, ΔG_S is calculated as 0.00 eV.

Based on these parameters, for **B-3-Me** and **C-1** in DCM, ΔG_S is calculated as 0.00 eV.

With reference **C-1** as electron acceptor, the ΔG(CS) = 0.74 – (–1.22)– 1.56-0.00 = 0.40 eV;

With reference **B-3-Me** as electron donor, the ΔG(CS) = 0.74 – (–1.22) – 2.09-0.00 = -0.13 eV;

In Toluene, ΔG_S is calculated as 0.70 eV.

With reference **C-1** as electron acceptor, the ΔG(CS) = 0.74 – (–1.22) – 1.56+0.74 = 1.14 eV;

With reference **B-3-Me** as electron donor, the ΔG(CS) = 0.74 – (–1.22) – 2.09+0.74 = 0.61 eV.

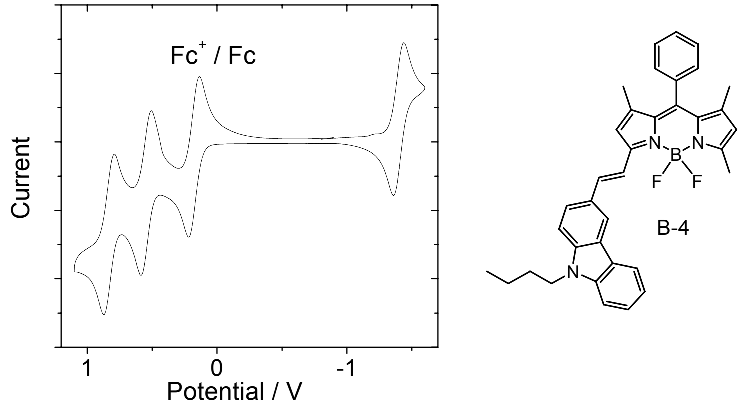

Fig. S67 Cyclic voltammogram of the compound **B-4**. Ferrocene (Fc) was used as internal reference ($E_{1/2}$ = +0.38 V (Fc^+/Fc) vs. SCE). In deaerated DCM solutions containing 0.5 mM photosensitizers alone, or with the ferrocene, 0.10 M Bu_4NPF_6 as supporting electrolyte, Ag/$AgNO_3$ reference electrode, Scan rates: 0.05 V/s.

6.0 The detailed data of fluorenscence lifetime.

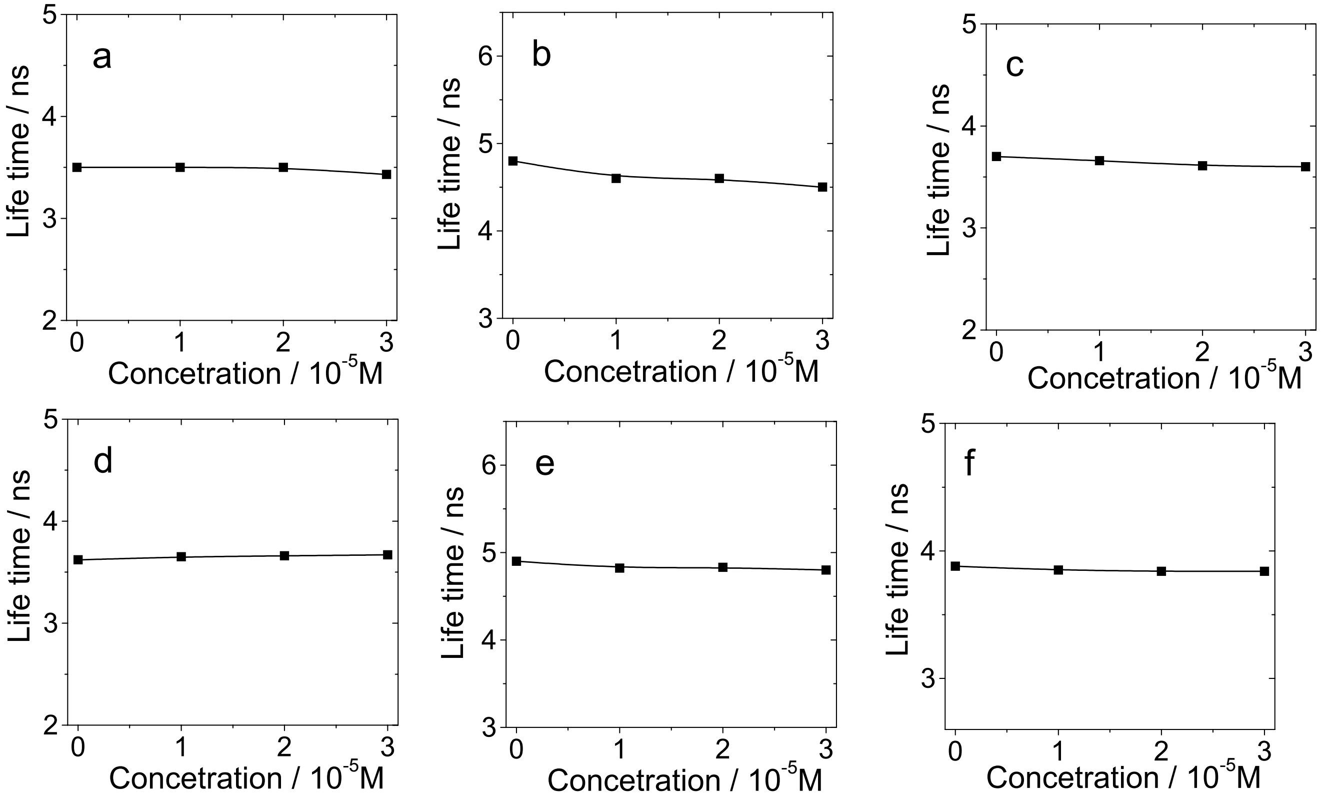

Fig. S68. Dependence of τ on the concentration of **C-1**: (a) **B-1** solution (1.0 × 10^{-5} M) in toulene, measurement wavelength 505 nm,(b) **B-2** solution (1.0 × 10^{-5} M) in toulene measurement wavelength 610 nm, (c) **B-3** solution (1.0 × 10^{-5} M) in toulene measurement wavelength 645nm. (d) **B-1-Me** solution (1.0 × 10^{-5} M) in toulene, measurement wavelength 505 nm,(e) **B-2-Me** solution (1.0 × 10^{-5} M) in toulene measurement wavelength 610 nm, (f) **B-3-Me** solution (1.0 × 10^{-5} M) in toulene measurement wavelength 645 nm.λ_{ex} = 475 nm. 20 °C.

7.0 Molecular dynamics (MD) simulations.

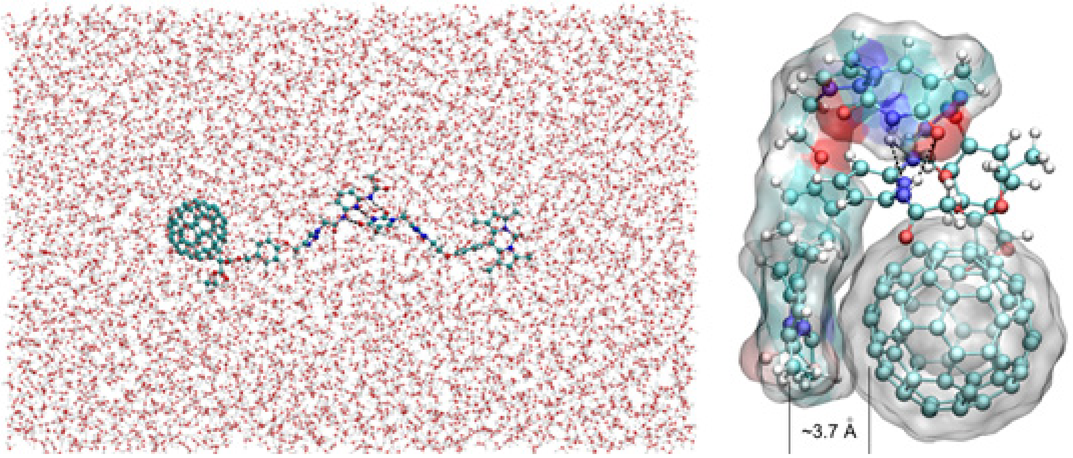

Fig. S69 Conformations of **B-1**-**C-1** complex in aqueous solution. Left: the starting extended conformation used in MD simulations. Right: Conformation taken from MD simulation at 9.5 ns, showing the hydrogen bonds (<3 Å as indicated using black dotted lines) between **B-1** and **C-1** motif, and π-π interactions between B-1 and fullerene motif of **C-1**. The white surfaces represent the solvent (water molecules) accessible surface areas for **B-1** and fullerene motif of **C-1** structures. Water molecules are not shown in the conformation at 9.5 ns for clarity.

The possible conformation of complex including hydrogen acceptor and donor was explored by molecular dynamics (MD) simulations performed in aqueous solution. It was found that a relative stable complex can be formed in less than 10 ns, indicating that the recognition of hydrogen donor and acceptor is rather feasible in aqueous solution. Note that three pairs of hydrogen bonds in the initial conformation are largely preserved in the compact conformation, and the π-π interactions between **B-1** and fullerene motif of **C-1** molecules further contribute the stability of such complex.

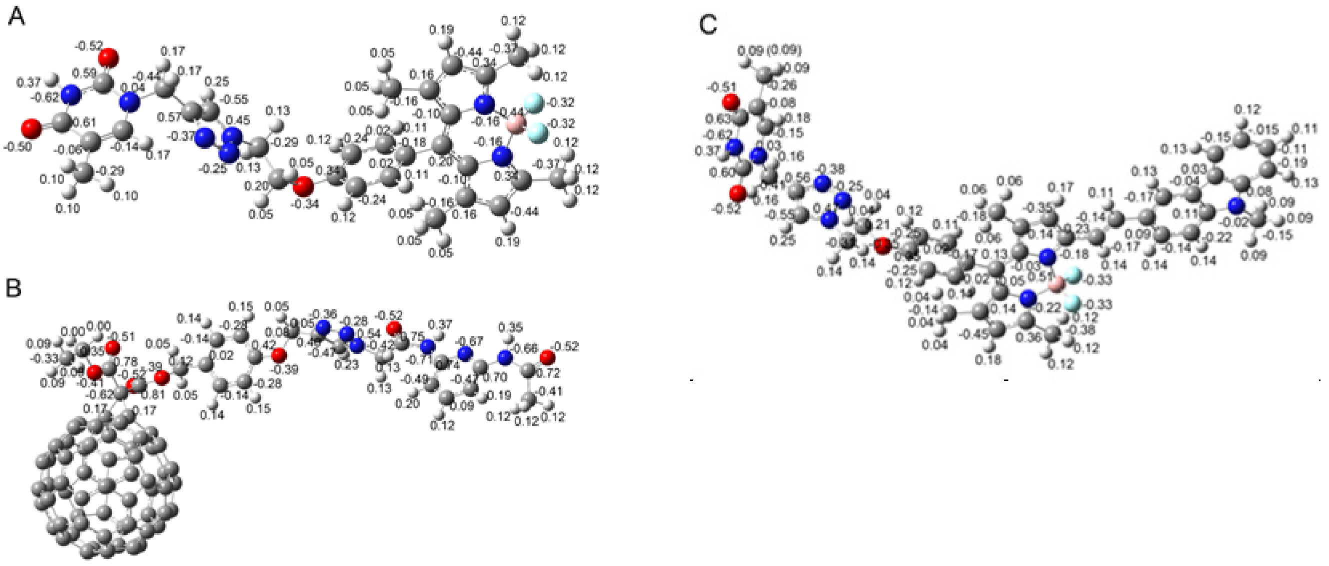

Fig. S70 Atomic partial charges calculated for (a) **B-1**, (b) **C-1**, and (c) **B-3** molecules by DFT method and used in the MD simulations.

8.0 The detailed data of Triplet-Triplet annihilation upconversion.

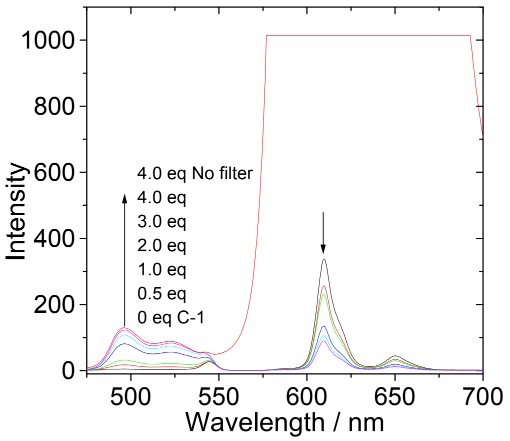

Fig. S71 TTA upconversions of **C-1** with H-bonding modules of **B-3**. **1-CBPEA** was used as triplet acceptor. The emission of **B-3** and **1-CBPEA** with increasing **C-1** added. Excited with 589 nm laser (4.8 mW, power density is 65 mW cm $^{-2}$) and a band-pass filter (470 nm – 570 nm) was used to suppress the scattered laser. c[Photosensitizers] = 1.0×10^{-5} M. c [1-CBPEA] = 1.2×10^{-4} M in deaerated toluene. 20 °C.

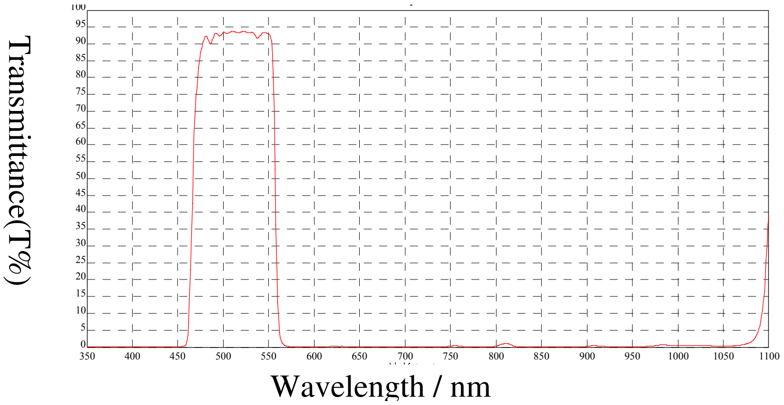

Fig. S72 The spectra of Transmittance as Y-axis and Wavelength as X-axis on the band-pass filter (470 nm-570 nm). The data was supplied by Giai photnics Co., Ltd (ShenZhen, China).
